# Generation and Analysis of Pyroptosis-Based and Immune-Based Signatures for Kidney Renal Clear Cell Carcinoma Patients, and Cell Experiment

**DOI:** 10.3389/fgene.2022.809794

**Published:** 2022-02-24

**Authors:** Gaoteng Lin, Qingfu Feng, Fangfang Zhan, Fan Yang, Yuanjie Niu, Gang Li

**Affiliations:** ^1^ Department of Urology, Tianjin Institute of Urology, The Second Hospital of Tianjin Medical University, Tianjin, China; ^2^ Department of Neurology, The Affiliated Hospital of Putian University, Putian, China

**Keywords:** kidney renal clear cell carcinoma, pyroptosis, ssGSEA, prognostic indices, immunotherapy, nomogram

## Abstract

**Background:** Pyroptosis is a programmed cell death caused by inflammasomes, which is closely related to immune responses and tumor progression. The present study aimed to construct dual prognostic indices based on pyroptosis-associated and immune-associated genes and to investigate the impact of the biological signatures of these genes on Kidney Renal Clear Cell Carcinoma (KIRC).

**Materials and Methods:** All the KIRC samples from the Cancer Genome Atlas (TCGA) were randomly and equally divided into the training and testing datasets. Cox and Least Absolute Shrinkage and Selection Operator (LASSO) regression analysis were used to screen crucial pyroptosis-associated genes (PAGs), and a pyroptosis-associated genes prognostic index (PAGsPI) was constructed. Immune-associated genes (IAGs) related to PAGs were identified, and then screened through Cox and LASSO regression analyses, and an immune-associated genes prognostic index (IAGsPI) was developed. These two prognostic indices were verified by using the testing and the Gene Expression Omnibus (GEO) datasets and an independent cohort. The patients’ response to immunotherapy was analyzed. A nomogram was constructed and calibrated. qRT-PCR was used to detect the expression of PAGs and IAGs in the tumor tissues and normal tissues. Functional experiment was carried out.

**Results:** 86 PAGs and 1,774 differentially expressed genes (DEGs) were obtained. After intersecting PAGs with DEGs, 22 differentially expressed PAGs (DEPAGs) were included in Cox and LASSO regression analyses, identifying 5 crucial PAGs. The PAGsPI was generated. Patients in the high-PAGsPI group had a poor prognosis. 82 differentially expressed IAGs (DEIAGs) were highly correlated with DEPAGs. 7 key IAGs were screened out, and an IAGsPI was generated. Patients in the high-IAGsPI group had a poor prognosis. PAGsPI and IAGsPI were verified to be robust and reliable. The results revealed patients in low-PAGsPI group and high-IAGsPI group may be more sensitive to immunotherapy. The calibrated nomogram was proved to be reliable. An independent cohort study also proved that PAGsPI and IAGsPI performed well in prognosis prediction. We found that the expression of AIM2 may affect proliferation of KIRC cells.

**Conclusion:** PAGsPI and IAGsPI could be regarded as potential biomarkers for predicting the prognosis of patients with KIRC.

## 1 Introduction

Renal cell carcinoma (RCC) is the third largest malignant tumor in urinary system, affecting more than 400,000 people worldwide and causing more than 170,000 deaths every year (Bray et al., 2018). In addition, the newly diagnosed cases of RCC in men are twice as many as those in women (Bray et al., 2018). It is worth noting that the most common solid renal cell carcinomas include clear-cell, papillary and chromophobe, out of which Kidney Renal Clear Cell Carcinoma (KIRC) accounts for about 70%–75% of the cases and is associated with poor prognosis and high mortality (Shuch et al., 2015). Surgery is still the most effective patient management strategy in the early stages of KIRC. Moreover, inactivation of the Von Hippel Lindau (VHL) leads to the dysfunction of hypoxia-inducible Factors (HIFs), which is the core molecular basis of the development and progress of KIRC (The Cancer Genome Atlas Research Network, 2013). Some patients with advanced KIRC can benefit from agents targeting downstream pathways, such as bevacizumab, pazopanib and sunitinib (The Cancer Genome Atlas Research Network, 2013). Previous studies have also demonstrated that a combination of targeted therapy and immune checkpoint inhibitor (ICI) led to a higher overall survival rate of patients with metastatic KIRC due to prolonged response (Sánchez et al., 2017). However, there are still a large number of patients with advanced KIRC who have no response to immunotherapy.

Cell death is as important as cell proliferation and differentiation. It is a necessary life process to ensure the normal development of multicellular organisms. Pyroptosis is a kind of programmed cell death (PCD), which was discovered as early as 1992 (Xia et al., 2019; Ruan et al., 2020). However, in 2015, it was found that gasdermin D (GSDMD) is the effector of pyroptosis, which is a breakthrough in the research of pyroptosis (Shi et al., 2015). It has been reported that pyroptotic cell death with morphological characteristics of apoptosis and necrosis was accompanied by the release of proinflammatory mediators, thus inducing inflammatory responses (Ruan et al., 2020). Notably, proper inflammatory responses will promote tissue repair and defense, accompanied by angiogenesis (Bergsbaken et al., 2009; Frangogiannis 2012). Nonetheless, excessive activation or inappropriate pyroptosis can be detrimental, leading to an increased risk of inflammatory diseases and cancer (Fang et al., 2020). Additionally, existing studies show that pyroptosis is closely related to various malignant tumors, including hepatocellular carcinoma, breast cancer, gastric cancer, lung cancer and other cancers that have not been studied (Xia et al., 2019). Some compounds inducing pyroptosis signaling pathway have also been approved for cancer therapy (Zheng and Li, 2020). In addition, cell pyroptosis can be regulated by epigenetics. DNA methylation, histone modification, nucleosome remodeling, and RNA-mediated targeting have been found to be involved in regulating crucial processes of tumorigenesis (Dawson and Kouzarides, 2012). The development of tagmentation-based whole-genome bisulfite sequencing made it possible to depict the complete picture of the methylome (Wang et al., 2013). Then, more and more studies focused on the influence of molecular methylation on diseases. Methyltransferase 4, N6-Adenosine (METTL4) is a candidate DNA m6A methyltransferase *in vivo*, and it has been proved that it can catalyze U2 m6A in fly *in vitro*, providing a novel insight into the enzymatic activity of METTL4 (Gu et al., 2020). Bromodomain Adjacent To Zinc Finger Domain 2A (BAZ2A) (TIP5) is overexpressed in prostate cancer, which is closely related to a molecular subtype displaying a CpG island methylator phenotype (CIMP) (Gu et al., 2015). They also found that high BAZ2A levels can predict the recurrence of tumor, which can be used as a useful biomarker to monitor tumor progression. A study has shown that environmental exposure can cause epigenetic alternation by combining DNA methylation, histone modification, and gene expression analyses, which may be linked to phenotype development (Bauer et al., 2016). And recently, Diao et al. (2020) have found hypothermia protects neurons against ischemia/reperfusion-induced pyroptosis via m6A-mediated activation of PTEN and the PI3K/Akt/GSK-3β signaling pathway, and Meng et al. (2022) have demonstrated that METTL14 suppresses pyroptosis and diabetic cardiomyopathy by downregulating TINCR lncRNA. Their studies showed the effect of epigenetics on pyroptosis. Then, chromatin state is not only related to cell differentiation, but also related to cell death (Benard et al., 2014; Blanco et al., 2021). Histone modifications in the promoter region of apoptosis gene reflect the transcriptional status of the gene, which can show whether tumor cells are sensitive to various anti-tumor treatments. However, the association between histone modifications and pyroptosis needs further study. As a novel form of cell death, biological mechanisms of pyroptosis are worthy of investigating.

The degree of infiltration of various immune cells varies with the type of tumor, which has a complex impact on tumor progression (Fridman et al., 2012). Some studies have demonstrated pyroptosis plays an important role in cancer immunity. (Deets and Vance, 2021). Notably, the cleavage and activation of gasdermin-E mediated by granzyme B released by natural killer (NK) cells and CD8^+^ T lymphocytes can induce pyroptosis of tumor cells (Zhang et al., 2020). It was also reported that upregulation of GSDME in tumor cells increased the infiltration of NK cells and antigen-specific CD8^+^ T lymphocytes, enhanced the secretion of antitumor effectors in tumor-infiltrating lymphocytes (TILs), and promoted tumor-associated macrophage-mediated phagocytosis of tumor cells (Tsuchiya, 2021). A previous study also reported that granzyme A can be produced by NK cells and CD 8 + T lymphocytes to cleave gasdermin-B in tumor cells, thus leading to pyroptosis (Zhou et al., 2020). The relationship between pyroptosis and the tumor microenvironment needs to be further studied. However, the effect of pyroptosis on KIRC remains unclear. Given that immune responses occur during the pyroptosis of tumor cells, the present study aimed to develop dual prognostic indices based on pyroptosis-associated and immune-associated genes in patients with KIRC. Additionally, this study attempts to further investigate the impact of the biological signatures of these genes on KIRC and provide novel insights for the treatment, especially immunotherapy.

## 2 Materials and Methods

### 2.1 Preparation of Data on the Kidney Renal Clear Cell Carcinoma Samples

The mRNA-seq profiles of 70 normal tissues and 541 tumor tissues, and clinical characteristics of the KIRC samples were downloaded from the Cancer Genome Atlas (TCGA) (https://portal.gdc.cancer.gov/). By searching for relevant studies on pyroptosis via PubMed, genes involved in the processes of pyroptosis are considered pyroptosis-associated genes (PAGs), including pyroptosis effector genes and relevant upstream and downstream regulation genes (Supplementary Table S1). In order to identify the function of these mediators, Gene Ontology (GO) and Kyoto Encyclopedia of Genes and Genomes (KEGG) enrichment analyses were performed on them via online web Metascape (http://metascape.org/gp/index.html#/main/step1). And the immune-associated genes (IAGs) were extracted from InnateDB (https://www.innatedb.com/) (Breuer et al., 2013). Then, all the KIRC samples from the TCGA were randomly divided into two equal subgroups with a ratio of 1:1. One group was taken as the training dataset to construct the prognostic indices, and the other was considered the testing dataset to validate these models. Another dataset with the characteristics of KIRC prognosis was obtained from the Gene Expression Omnibus (GEO) (GSE29609) (https://www.ncbi.nlm.nih.gov/geo/query/acc.cgi?acc=GSE29609) (Edeline et al., 2012), to verify the performance of the dual prognostic models developed in the TCGA training cohort. By deleting missing and unknown values, the clinical dataset was prepared for further analysis.

### 2.2 Development of a Prognostic Index Based on Pyroptosis-Associated Genes

The mRNA-seq data of KIRC samples in the training dataset were analyzed to get differentially expressed genes (DEGs), using the “edgeR” package in R (version 4.0.5) (Love et al., 2014), based on the following criteria: | log2-fold change (FC) | > 1 and a false discovery rate (FDR) < 0.05. First, the DEGs were intersected with PAGs to get the differentially expressed pyroptosis-associated genes (DEPAGs). Thereafter, DEPAGs were screened through univariate Cox regression analysis. Next, in the univariate Cox regression analysis, the DEPAGs with *p* < 0.1 were further subjected to the Least Absolute Shrinkage and Selection Operator (LASSO) regression analysis (Kukreja et al., 2006), to avoid overfitting and generate crucial DEPAGs. Then, the crucial DEPAGs were included in multivariate Cox regression analysis to identify the independent DEPAGs that influence prognosis and calculate their corresponding regression coefficient in the model. Finally, the pyroptosis-associated genes prognostic index (PAGsPI) was generated based on these crucial DEPAGs filtered by LASSO regression analysis, by multiplying the expression of these crucial DEPAGs by their corresponding regression coefficient generated by multivariate Cox regression analysis, then obtaining the sum. Additionally, patients in the training dataset were divided into the high-PAGsPI and low-PAGsPI groups based on the median value of PAGsPI. Then, Kaplan–Meier (KM) survival analysis was performed on the independent DEPAGs and PAGsPI to evaluate their prognostic values. Moreover, the receiver operating characteristics (ROC) curve was drawn to assess the ability of PAGsPI to predict the overall survival rate of 1, 3, and 5 years in patients with KIRC.

### 2.3 Transcriptional and Potential Regulatory Analyses

It is very important to identify how to transcribe and regulate independent prognostic DEPAGs in pyroptosis. Therefore, in this study, the transcription factors of independent prognostic DEPAGs were researched from JASPAR (http://jaspar.genereg.net/) (Fornes et al., 2020), using NetworkAnalyst (https://www.networkanalyst.ca/) (Zhou et al., 2019). In addition, data of interaction between miRNA and mRNA and the data of interaction between lncRNA and miRNA were obtained from RNAInter (http://www.rna-society.org/rnainter/home.html) (Lin et al., 2020), to develop the lncRNA-miRNA-mRNA regulatory network (ceRNA network) for independent DEPAGs. Graphic visualization was performed in the Cytoscape software (version 3.7.2).

### 2.4 The Association of the Expression of Differentially Expressed PAGs With Clinical TNM Staging

The association between the expression of independent prognostic DEPAGs and clinical TNM stages was analyzed to investigate the potential impact of pyroptosis on tumor progression.

### 2.5 Analysis of Different Types of Immune Cell Infiltration Based on the Pyroptosis-Associated Genes Prognostic Index Levels

The immune and stromal scores of KIRC patients were downloaded from ESTIMATE (Estimation of STromal and Immune cells in MAlignant Tumor tissues using Expression data, https://bioinformatics.mdanderson.org/estimate/) (Yoshihara et al., 2013), to explore differences of tumor-infiltrating immune cells between high-PAGsPI group and low-PAGsPI group. Immune score refers to the infiltration level of immune cells in tumor tissue while stromal score represents level of infiltrating stromal cells. After that, a single sample gene set enrichment analysis (ssGSEA) was performed to calculate the score of each type of immune cell, so as to further investigate the infiltration differences and the potential immune-related pathways. To further investigate the relationship between pyroptosis and immunity, we explored the difference of the seven steps of the tumor-immune cycle scores and the infiltration of immune cells between high-PAGs group and low-PAGs group by online web Tracking Tumor Immunophenotype (TIP) (http://biocc.hrbmu.edu.cn/TIP/index.jsp) (Xu et al., 2018). The cancer-immunity cycle depicts the processes from antigen release to tumor killing, which mainly includes seven steps: 1) release of cancer cell antigens, 2) cancer antigen presentation, 3) initiation and activation, 4) transmitting immune cells to tumors, 5) immune cells infiltrating into tumors, 6) T cells recognizing cancer cells, 7) T cell killing cancer cells, which are closely related to immunotherapy of tumor. Finally, we also analyzed the association between expression of independent DEPAGs and immune cells infiltration by online web Tumor IMmune Estimation Resource (TIMER) (https://cistrome.shinyapps.io/timer/) (Li et al., 2017).

### 2.6 Identification of Immune-Associated Genes Linked to Pyroptosis and Generation of a Prognostic Index

The differentially expressed immune-associated genes (DEIAGs) were obtained by intersecting the differentially expressed genes with IAGs. Then, we calculated a pairwise correlation between each DEIAG and each DEPAG by Pearson correlation analysis. We selected the DEIAGs with good correlation coefficient with DEPAGs as DEIAGs related to DEPAGs based on the rigorous criteria of | Pearson correlation coefficient | ≥ 0.5 and *p* < 0.05. If DEIAGs have an opposite correlation with different DEPAGs, and they meet the criteria at the same time, they would be discarded to ensure the same direction correlation. These DEIAGs were selected for further analysis. Similarly, univariate Cox regression analysis was used to screen the prognostic IAGs, and LASSO regression analysis was used to further screen and produce key IAGs. After that, the multivariate Cox regression analysis was used to identify independent prognostic IAGs, and the regression coefficient for each key IAGs was calculated. Subsequently, the immune-associated genes prognostic index (IAGsPI) was constructed by multiplying the expression of the key IAGs filtered by LASSO regression analysis by their corresponding coefficient, and getting the sum. In addition, based on the median value of IAGsPI, patients in the training dataset were divided into high-IAGsPI group and low-IAGsPI group. Then, the prognostic values of independent IAGs and IAGsPI were evaluated through KM survival analysis. The ROC curve was also used to estimate the ability of IAGsPI to predict the overall survival rate of 1, 3, and 5 years in patients with KIRC.

### 2.7 Correlation Between the Expression of Differentially Expressed IAGs and Clinical TNM Staging

The correlation between the expression of DEIAGs and clinical TNM stages was analyzed to explore their effect on tumor behavior.

### 2.8 Internal and External Validation for Pyroptosis-Associated Genes Prognostic Index and Immune-Associated Genes Prognostic Index

The robustness and reliability of PAGsPI and IAGsPI were assessed in the testing dataset and the GEO dataset and an independent cohort (as shown below). PAGsPI and IAGsPI were generated in the testing dataset and GEO dataset, respectively, based on the crucial PAGs and key IAGs determined in the training dataset. Additionally, KM survival analysis and the ROC curve were utilized to analyze their prognosis and predictive performance.

### 2.9 Development and Calibration of a Nomogram for the Prediction of Prognosis

Combined with clinical characteristics, Cox regression analysis was conducted on age, gender, tumor stage, grade, T stage, M stage, PAGsPI and IAGsPI, and the risk factors affecting prognosis were screened. Thereafter, based on these factors, a nomogram was developed to predict 1-year, 3-year and 5-year prognosis of patients with KIRC. The calibration curves and concordance index (C-index) were used to evaluate the model performance.

### 2.10 Evaluation of Response to Immunotherapy

It was reported that only subsets of patients with malignant tumors responded to immunotherapy (Riley et al., 2019). Therefore, it is important to evaluate whether patients will respond to immunotherapy. In this study, the differences of CTLA4, GAL9, LAG3, PD1 and PDL1 expression between the PAGsPI and IAGsPI subgroups were analyzed at first. Tumor immune dysfunction and exclusion (TIDE) has advantages in assessing tumor immune escape by two mechanisms, and has been considered as a promising immunotherapy biomarker to predict patients’ immunotherapy response (Jiang et al., 2018). In this section, we used four biomarkers to assess patients’ response to immunotherapy: TIDE, MSI, T cell dysfunction and T cell exclusion. Then, relevant data were obtained from the website of TIDE (http://tide.dfci.harvard.edu/) (Jiang et al., 2018) after uploading a normalized expression matrix.

### 2.11 Functional Enrichment Analysis of Differentially Expressed Genes in the Pyroptosis-Associated Genes Prognostic Index and Immune-Associated Genes Prognostic Index Subgroups

GO and KEGG enrichment analyses were used to analyze differentially expressed genes, between the PAGsPI subgroups, to investigate the biological signatures, and potential regulatory pathways for tumor progression. The same analysis was carried out for IAGsPI subgroups.

### 2.12 Validation of the Prognostic Powers for Pyroptosis-Associated Genes Prognostic Index and Immune-Associated Genes Prognostic Index by an Independent Cohort

From February 2019 to March 2020, a total of 27 pairs of KIRC tumor tissues were collected from the Second Hospital of Tianjin Medical University as the independent cohort. All patients were informed and signed informed consents. The samples used in the study were approved by the Institutional Review Boards of the Second Hospital of Tianjin Medical University. All tissues were frozen at −80°C. Grinding the tissue first, the TRIzol reagent (Invitrogen, China) was used to extract total RNA from KIRC tissues, following the manufacturer’s protocol. In addition, cDNA was reverse transcribed from RNA using the RevertAid First Strand cDNA Synthesis Kit (Thermo Scientific; United States), according to the manufacturer’s guidelines. All the RNAs and cDNAs were stored at −80°C. Moreover, the cDNA was subjected to quantitative reverse transcription–polymerase chain reaction (qRT–PCR) using the FastStart Universal SYBR Green Master (ROX, Roche; United States). GAPDH was selected as the internal control, and the relative expressions of PAGs and IAGs were calculated by 2^−ΔΔCT^ method. The primer sequences used in this study were listed in Supplementary Table S4.

### 2.13 Influence of the Significant Pyroptosis-Associated Genes on KIRC Proliferation

#### 2.13.1 Selecting and Culturing KIRC Cell Lines

Two human KIRC cell lines, CAKI-1 and ACHN, were obtained from the American Type Culture Collection. The cells were cultured in RPMI 1640 medium supplemented with 10% fetal bovine serum and 1% penicillin/streptomycin at 37°C in 5% CO_2_ incubator.

#### 2.13.2 Cell Transfection

The small interfering RNA (siRNA) of negative control and AIM2 were designed and synthesized by GenePharma (Shanghai, China). A total of 8 × 105 CAKI-1 and ACHN cells were seeded in 6-well plates. Twenty-four hours later, the cells were transfected with 100 nM siRNA of negative control (5′-UUC​UCC​GAA​CGU​GUC​ACG​UTT-3′) and AIM2 (5′-GCC​UGG​AUA​ACA​UCA​CUG​ATT-3′) using LipofectamineTM 2000 (Invitrogen) in terms of the manufacturer’s protocol. After incubation for 48 h, the cells were collected for qRT–PCR and MTT assays.

#### 2.13.3 MTT Assay

Cell viability was tested using the 3-(4,5-dimethylthiazol-2-yl)-2,5-diphenyl-2H-tetrazolium bromide (MTT) assay according to the manufacturer’s instructions. After transfection for 48 h, 2.0 × 103 cells per well were seeded into 96-well plates and incubated for 24 h, 48 h, 72 h, and 96 h at 37°C. Then, 30 ul MTT solution was added to the cells and incubated at 37°C for another 2 h in the dark. Subsequently, 150 ul dimethyl sulfoxide (DMSO) was added into each well to dissolve formazan crystals following sucking out the MTT solution of per well. The absorbance of these wells was measured at 490 nm. All measurements were conducted in sextuplicate.

#### 2.13.4 Clone Formation Assay

2.0 × 103 cells in each group were seeded into 6-well plate. Twenty-four hours later, the cells were transfected with AIM2 siRNA and negative control siRNA, and cultured for 1 week. After colony was formed, it was fixed with 4% paraformaldehyde for 30 min, and then slowly washed with phosphate-buffered saline (PBS) buffer twice. Then, the plate was stained with crystal violet solution for 30 min, and washed again with PBS and dried in air. The number of clones was calculated by ImageJ software.

#### 2.14 Statistical Analysis

All statistical analyses were performed in the R software (version 4.0.5) and GraphPad Prism (version: 8.0.1). In addition, differential plots of group comparisons were drawn in the GraphPad Prism and R software. Wilcox test or T test was used to compare continuous variables, and Pearson correlation analysis was used to assess the correlation among genes. Moreover, Cox and LASSO regression analyses were performed by using “survival” and “glmnet” packages in the R software. Evaluation of survival was conducted through KM survival analysis in the R software. The ROC curve was drawn using the “survival ROC” package in R software. *p* < 0.05 was considered to be statistically significant.

## 3 Results

### 3.1 Obtaining Differentially Expressed PAGs and Constructing a Prognostic Index

As shown in Supplementary Table S1, a total of 86 PAGs were obtained from previous studies.

**FIGURE 1:**
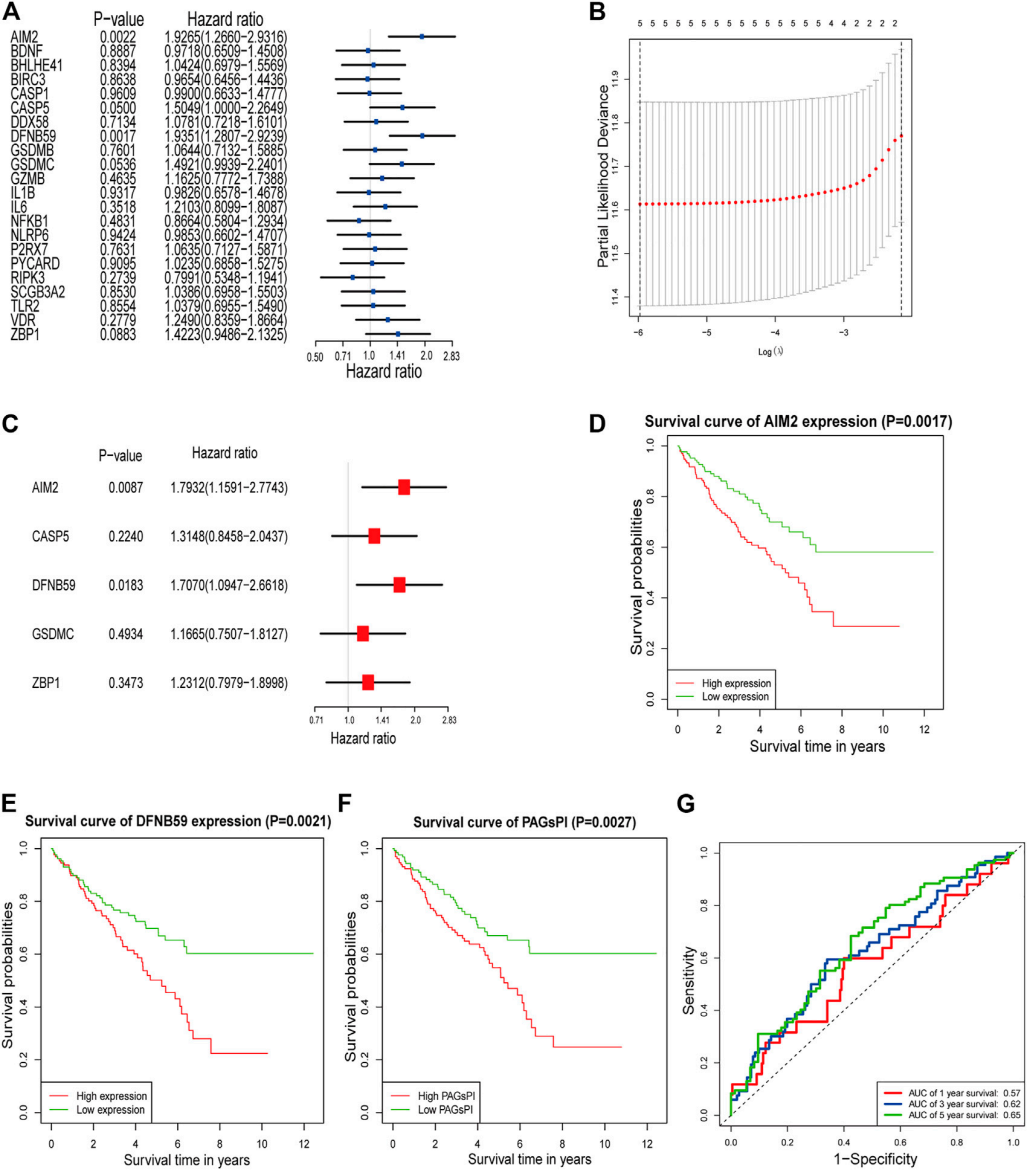
Identification of PAGs linked to overall survival and construction of PAGsPI. **(A)** Univariate Cox regression analysis results of the 22 DEPAGs. **(B)** The results of LASSO regression analysis, identifying minimum criteria. **(C)** The results of multivariate Cox regression analysis, determining independent prognostic factors and calculating their coefficient. **(D–F)** KM survival analysis of AIM2, DFNB59 and PAGsPI. **(G)** ROC curve of PAGsPI for predicting 1-year, 3-year and 5-year survival.

The result of GO analysis demonstrated that these PAGs were involved in GO:0070269: pyroptosis, GO:0002683: negative regulation of immune system process and other biological processes (Supplementary Figure S1). The statistics and analysis results of clinical characteristics of the training and testing datasets were given in Supplementary Table S2. In the training dataset, differentially expressed analysis in KIRC samples (35 normal samples vs 271 tumor samples) identified 1,774 DEGs, including 543 downregulated genes and 1,231 upregulated genes (Supplementary Figure S2A). Then, the DEGs were intersected with PAGs, and 22 DEPAGs were obtained (Supplementary Figure S2B). Out of 22 DEPAGs, 5 DEPAGs with *p* < 0.1 in the univariate Cox regression analysis were selected for further analysis (Figure 1A). In order to minimize the risk of over-fitting, the 5 DEPAGs were filtered by LASSO regression analysis and the crucial DEPAGs were generated (Figure 1B). A total of 5 DEPAGs were regarded as crucial DEPAGs. Multivariate Cox regression analysis was carried out, in which 2 DEPAGs were identified as independent prognostic factors, and corresponding regression coefficient of 5 crucial DEPAGs were calculated. Absent In Melanoma 2 (AIM2) (HR: 1.7932; 95% CI: 1.1591–2.7743; *p* = 0.0087) and Pejvakin (DFNB59) (HR: 1.7070; 95% CI: 1.0947–2.6618; *p* = 0.0183) were the independent PAGs which affect overall survival rate (Figure 1C). Moreover, the pyroptosis-associated genes prognostic index (PAGsPI) was constructed based on the 5 crucial DEPAGs as follows: PAGsPI = (0.584 × expression of AIM2) + (0.2736 × expression of CASP5) + (0.5347× expression of DFNB59) + (0.154 × expression of GSDMC) + (0.208 × expression of ZBP1). The KIRC samples were divided into the high-PAGsPI group and low-PAGsPI group based on the median value of PAGsPI, which was 0.86 in this case. There were 133 patients in the high-PAGsPI group while 134 individuals in the low-PAGsPI group. The prognostic values of two independent PAGs and PAGsPI were further investigated by KM survival analysis. The results showed that the prognosis of patients with high expression of AIM2 and DFNB59 was worse than that of patients with low expression (Figures 1D,E). Moreover, the mortality of patients in high-PAGsPI group was higher than that of patients in low-PAGsPI category (Figure 1F). In the training dataset, the area under the PAGsPI ROC curve used for 1-year, 3-year, and 5-year survival rate prediction was 0.57, 0.62, and 0.65, respectively (Figure 1G), demonstrating a moderate prediction ability.

### 3.2 Relevant Transcription Factors and the ceRNA Network for the 2 Independent Pyroptosis-Associated Genes

**FIGURE 2:**
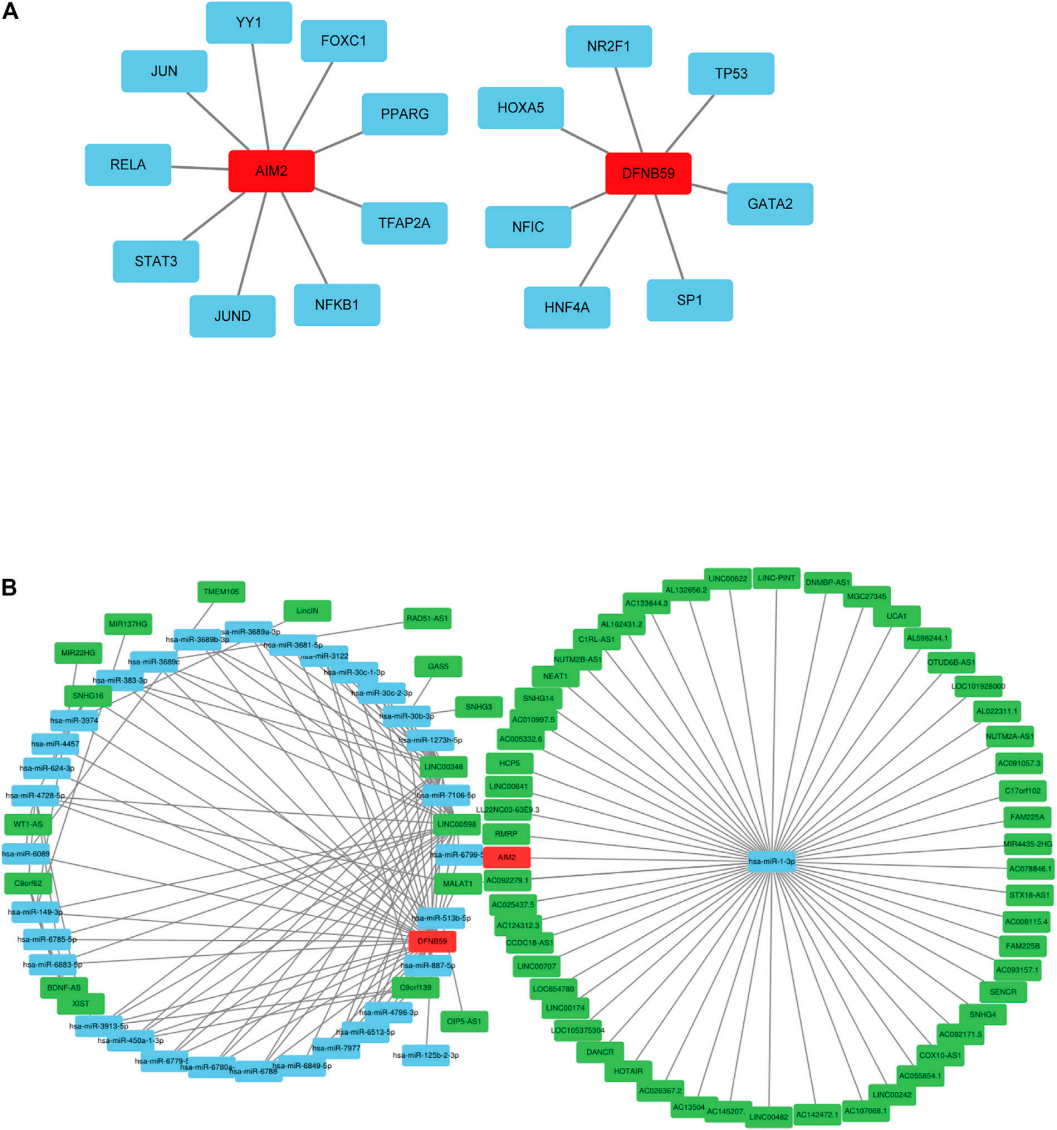
Potential regulation of the independent prognostic PAGs. **(A)** Transcription factors of AIM2 and DFNB59. The red squares represent AIM2 and DFNB59, while the blue squares indicate their transcription factors. **(B)** The lncRNA-miRNA-mRNA (ceRNA) network for AIM2 and DFNB59. The green squares indicate lncRNAs, blue squares represent miRNAs and red squares depict mRNAs.

Analysis of related databases revealed that 9 transcription factors for AIM2 and 7 transcription factors for DFNB59 (Figure 2A). In this study, the potential lncRNA-miRNA-mRNA (ceRNA) regulatory pathway of the 2 independent PAGs was investigated. A ceRNA network composed of 73 lncRNAs, 33 miRNAs and 2 mRNAs was determined (Figure 2B). These findings provide a deeper understanding of the potential regulatory mechanisms of pyroptotic tumor cells.

### 3.3 The Expression Levels of Independent Pyroptosis-Associated Genes in Different TNM Stages

**FIGURE 3:**
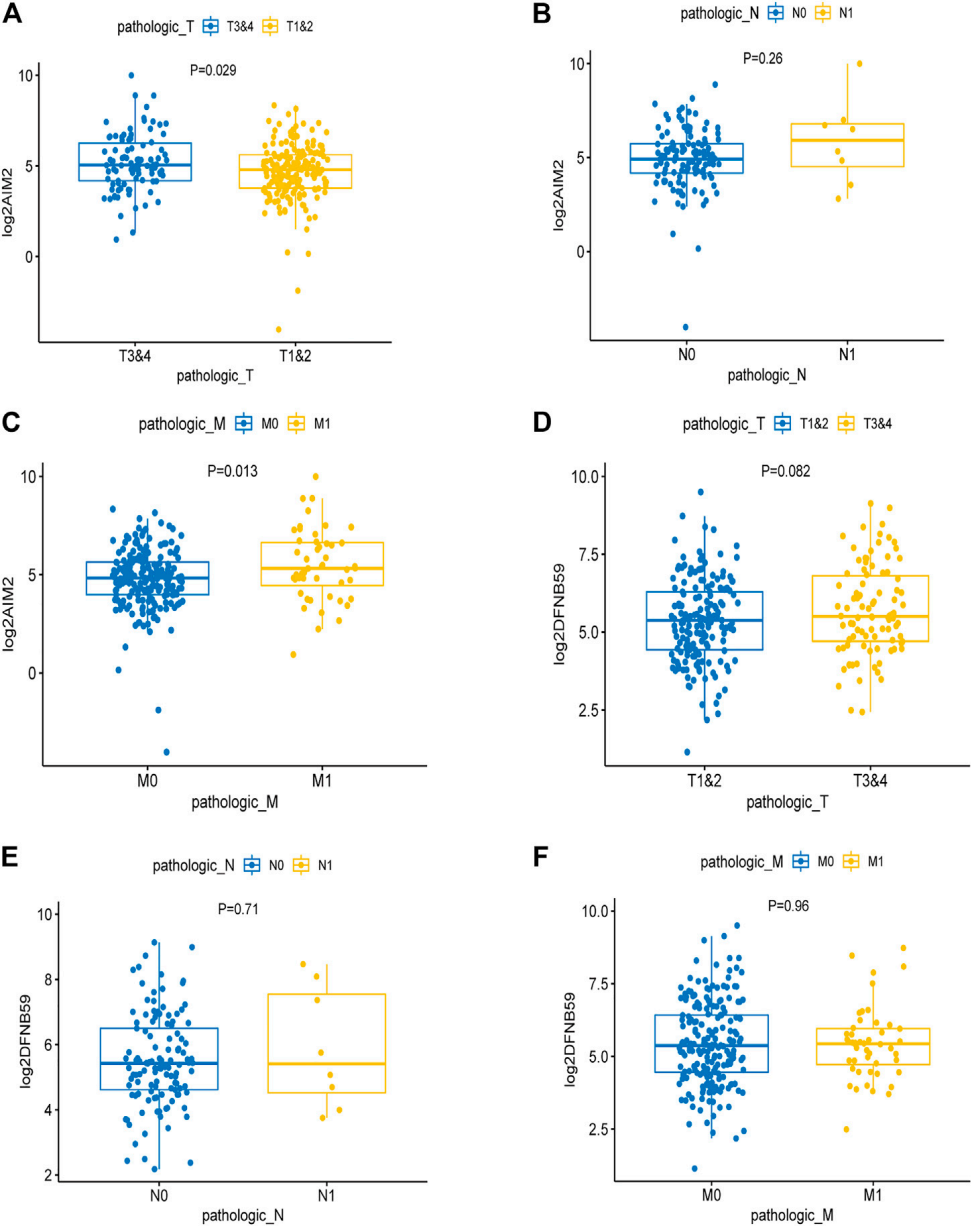
The effect of the expression of independent PAGs on tumor progression. **(A,C)** The expression of AIM2 in the advanced T and M1 stages was higher than that in the early T and M0 stages. **(B)** There was no difference in the expression of AIM2 in different N stage. **(D–F)** The expression of DFNB59 in different TNM stages, with no statistical significance.

Analysis of the expression levels of AIM2 and DFNB59 in different TNM stages revealed that high expression of AIM2 was closely related to the advanced T and M stage (Figures 3A,C), which indicates that it may facilitate the development and distant metastasis of tumors. High expression of AIM2 may not promote lymph node metastasis of tumor (Figure 3B). However, there were no different expressions of DFNB59 in different TNM stages (Figures 3D–F). In view of the fact that it is not clear whether lymph node metastasis occurs in many patients with KIRC, it was impossible to clearly show the effect of the expression of AIM2 and DFNB59 on the lymph node metastasis of tumor cells.

### 3.4 Tumor-Infiltrating Immune Cells in the Pyroptosis-Associated Genes Prognostic Index Subgroups

**FIGURE 4:**
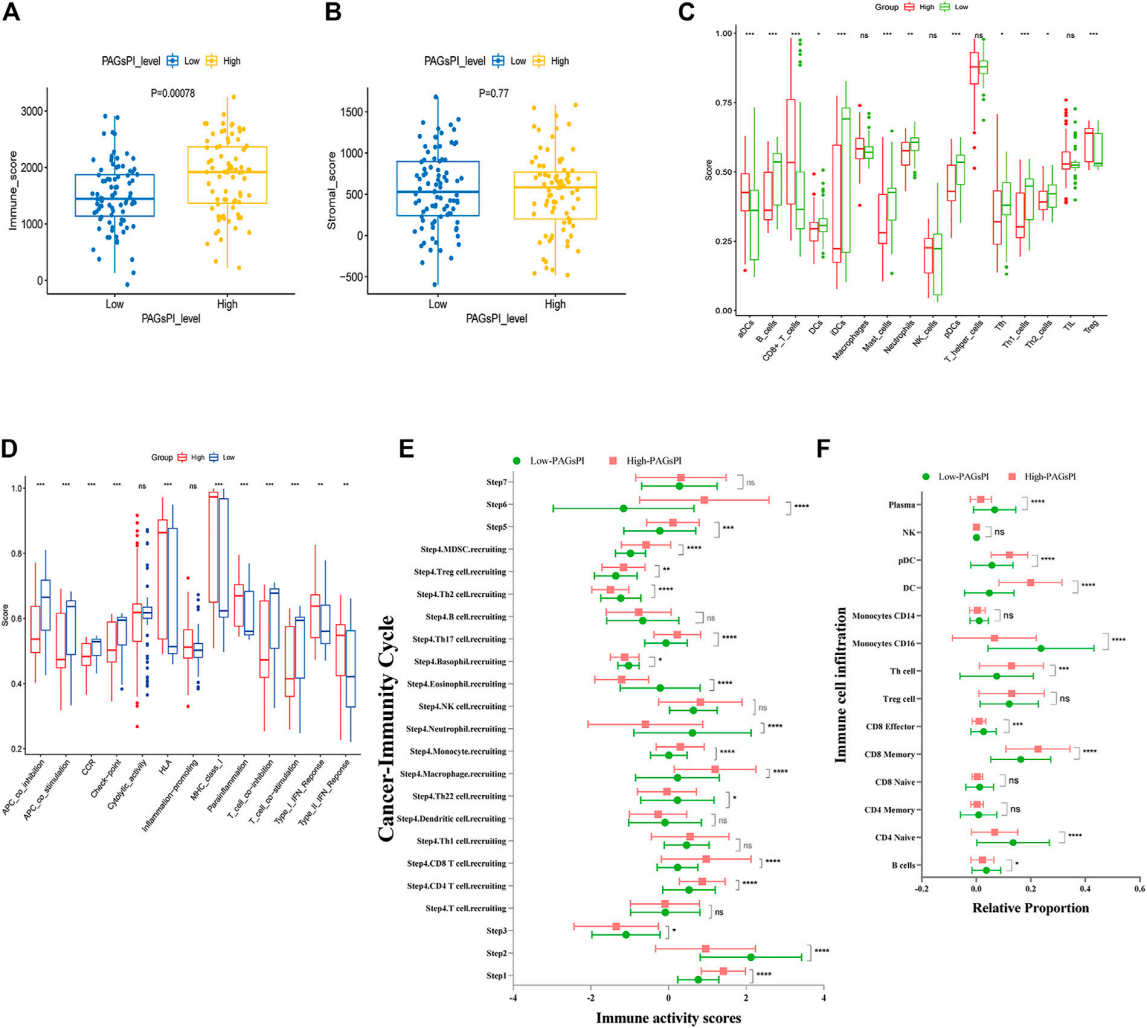
Immune analysis of the PAGsPI subgroup. **(A)** Comparison of the immune score in the PAGsPI subgroup by ESTIMATE. **(B)** Comparison of the stromal score in the PAGsPI subgroup by ESTIMATE. **(C)** Analyzing the types of immune cell infiltration in the PAGsPI subgroup by ssGSEA. **(D)** Immune-associated pathways by ssGSEA. **(E)** Profiling the status of anti-cancer immunity across seven-step Cancer-Immunity Cycle by TIP. **(F)** Analyzing the types of immune cell infiltration in the PAGsPI subgroup by TIP.

At first, the differences of tumor-infiltrating immune cells between PAGsPI subgroups were assessed by immune score and stromal score. The findings revealed that the immune score in the high-PAGsPI group was higher than that in the low-PAGsPI category (Figure 4A). The higher the immune score is, the more immune cells infiltrate. There was no significant difference in stromal scores between PAGsPI subgroups (Figure 4B). These results indicated that there was more infiltration of immune cell in the high-PAGsPI group. The study further investigated the type of infiltrating immune cells by ssGSEA. The results showed that the number of active dendritic cells, CD8^+^ T cells and regulatory T cells in high-PAGsPI group was higher than that in low-PAGsPI group (Figure 4C). The infiltration of B cells, mast cells, neutrophils and T helper cells was higher in the low-PAGsPI group. Additionally, the immune-related pathways in the high-PAGsPI group included the Human Leukocyte Antigen (HLA), MHC class I, para-inflammation, Type I IFN response and Type II IFN response (Figure 4D). And in the low-PAGsPI group, antigen presenting cell (APC) co-inhibition, APC co-stimulation, chemokine receptors (CCR), check point, T cell co-inhibition and T cell co-stimulation were involved in immune regulation. By the analysis of cancer-immunity cycle, we analyzed the difference of the seven steps of the tumor-immune cycle scores and the infiltration of immune cells between high-PAGsPI group and low-PAGsPI group. The result showed that apart from the step 7, other steps were statistically different between high-PAGsPI group and low-PAGsPI group (Figure 4E). Patients in high-PAGsPI group may recruit more CD8^+^ T cells and CD4^+^ T cells to infiltrate. Then, by the analysis of immune cells infiltration, we found in the subtype of CD8^+^ T cells, CD8^+^ effectors were less recruited in the high-PAGsPI group while CD8^+^ memory cells were more recruited (Figure 4F), which was related to prognosis and immunotherapy responsiveness. These results showed patients in the high-PAGsPI group may have poorer ability to anti-tumor than those in the low-PAGsPI group. Finally, we found the expression of AIM2 was associated with the infiltration of B cells, CD8^+^ T cells and CD4^+^ T cells etc. The expression of DFNB59 was associated with the infiltration of B cells, CD8^+^ T cells, CD4^+^ T cells and Dendritic cells (Supplementary Figure S3).

### 3.5 Differentially Expressed IAGs Correlated With Pyroptosis and Developing a Prognostic Index

**FIGURE 5:**
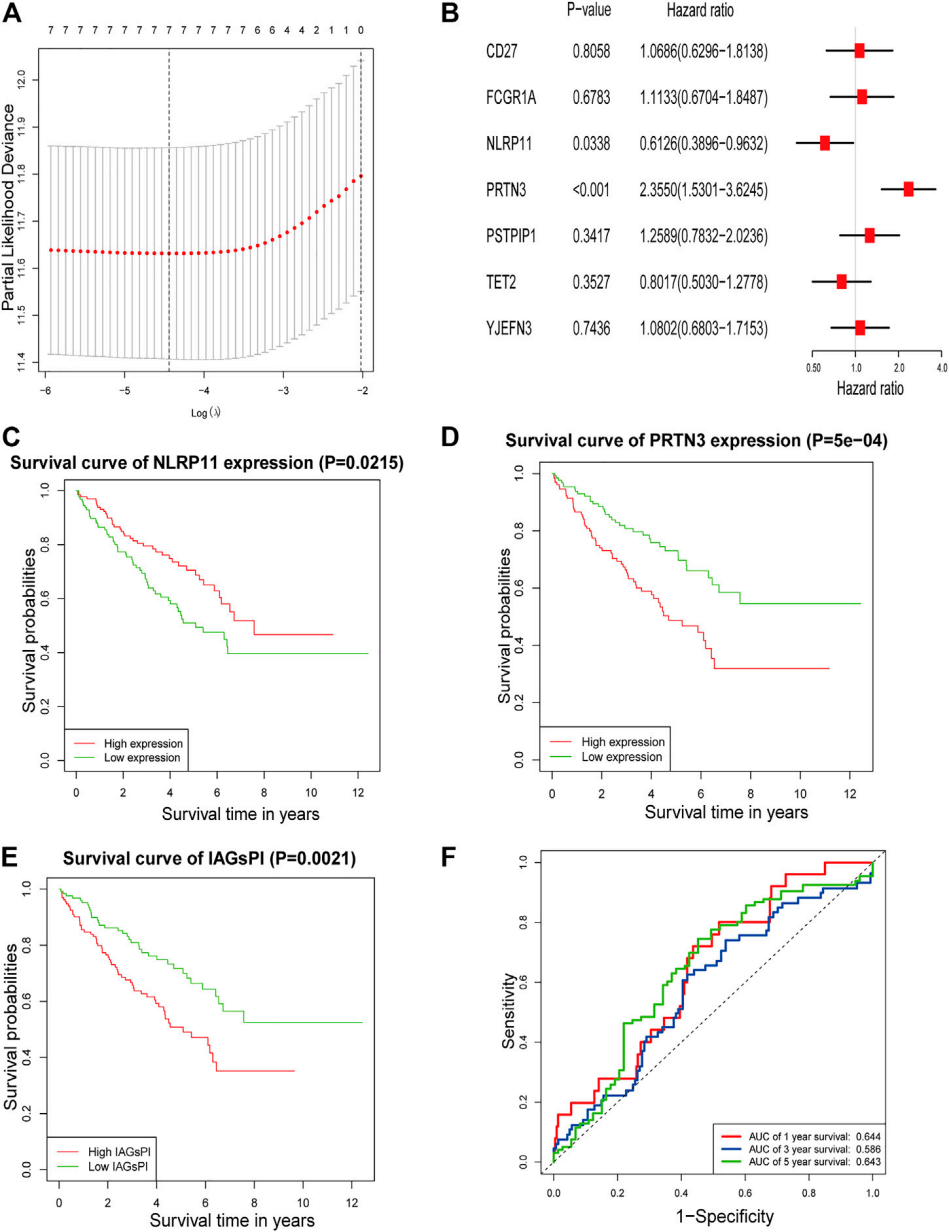
Screening IAGs that were highly related to PAGs and development of a IAGsPI. **(A)** The results of LASSO regression analysis, identifying minimum criteria. **(B)** The results of multivariate Cox regression analysis on 7 IAGs were used to identify independent prognostic immune genes, and the corresponding coefficients of the 7 IAGs were calculated. **(C–E)** KM survival analysis for NLRP11, PRTN3 and IAGsPI, respectively. **(F)** Prediction of ROC curve based on IAGsPI’s 1-year, 3-year and 5-year survival rate.

After the intersection of DEGs and IAGs, 116 DEIAGs were obtained. We calculated a pairwise correlation between each DEIAG and each DEPAG. Based on | Pearson correlation coefficient | ≥ 0.5 and *p* < 0.05, Pearson correlation analysis showed a total of 82 DEIAGs were highly correlated with DEPAGs (Supplementary Table S3). Among these 82 DEIAGs, 7 DEIAGs with *p* < 0.1 in the univariate Cox regression analysis were chosen for further analysis. These 7 DEIAGs were further subjected to LASSO regression analysis, and 7 DEIAGs were the key IAGs (Figure 5A). Then, multivariate Cox regression analysis was performed on the 7 IAGs, and 2 IAGs that were independent prognostic factors were determined, and the corresponding coefficients of the 7 IAGs were calculated (Figure 5B). NLR Family Pyrin Domain Containing 11 (NLRP11) (HR: 0.6126; 95% CI: 0.3896–0.9632; *p* = 0.0338) and Proteinase 3 (PRTN3) (HR: 2.355; 95% CI: 1.5301–3.6245; *p* < 0.001) were the independent risk immune genes that affect the prognosis. Subsequently, the immune-associated genes prognostic index (IAGsPI) based on the 7 IAGs was developed as follows: IAGsPI = (0.06638 × expression of CD27) + (0.10734 × expression of FCGR1A) − (0.49005 × expression of NLRP11) + (0.85653 × expression of PRTN3) + (0.23027 × expression of PSTPIP1) − (0.22103 × expression of TET2) + (0.07718 × expression of YJEFN3). The median value of IAGsPI was 0.94, which is the cutoff value to divide the KIRC samples into the high-IAGsPI group and low-IAGsPI group. There were 133 samples in the high-IAGsPI group and 134 in the low-IAGsPI group. The analysis of KM survival rate of 2 independent IAGs which influenced the prognosis showed that the mortality of patients with high expression of NLRP11 decreased (Figure 5C), while the prognosis of patients with high expression of PRTN3 was poor (Figure 5D). In addition, KM survival analysis showed that the prognosis of patients in the high-IAGsPI group was worse than those in the low-IAGsPI group (Figure 5E). In addition, the area of 1-year, 3-year and 5-year survival rates prediction under the ROC curve of IAGsPI were 0.644, 0.586 and 0.643, respectively (Figure 5F), which indicates the prediction performance was reliable.

### 3.6 The Expression of IAGs at Different TNM Stages

The effect of 2 independent IAGs on tumor behavior was investigated by analyzing their expressions in different TNM stages. The results showed that NLRP11 had no obvious difference in different TNM stages (Supplementary Figures S4A–C). There was no significant difference in the expression of PRTN 3 in different TNM stages (Supplementary Figures S4D–F). Furthermore, it is not clear whether many patients with KIRC had lymph node metastasis or not, and it was impossible to determine the impact of the expression of the 5 IAGs on tumor lymph node metastasis.

### 3.7 Reliability of Pyroptosis-associated Genes Prognostic Index and Immune-Associated Genes Prognostic Index

**FIGURE 6:**
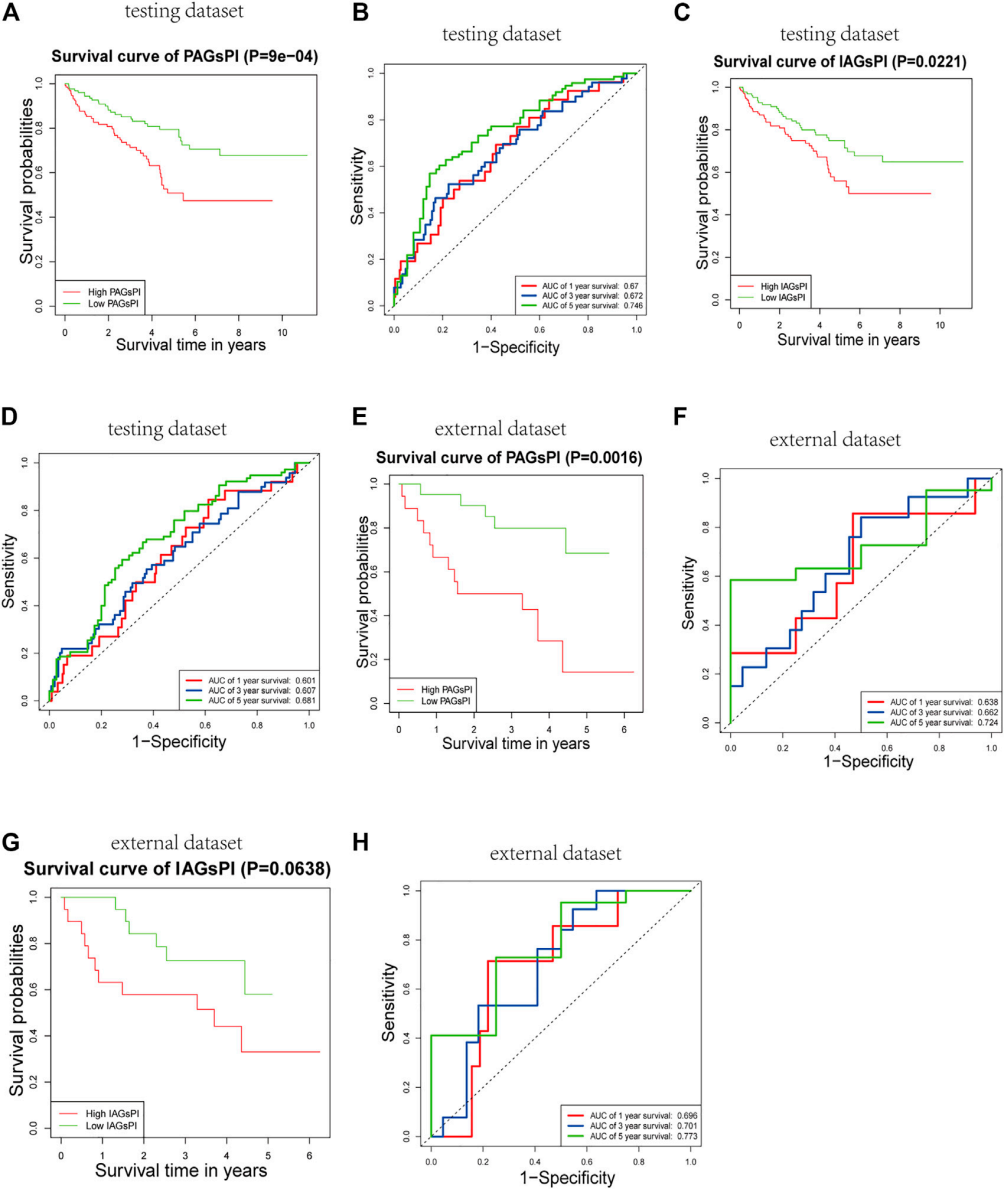
Internal and external validation of the prognostic indices. **(A,B)** Results of KM survival analysis and the ROC curve for PAGsPI, in the testing dataset. **(C,D)** Results of KM survival analysis and the ROC curve for IAGsPI, in the testing dataset. **(E,F)** Results of KM survival analysis and the ROC curve of PAGsPI, in the external dataset. **(G,H)** Results of KM survival analysis and the ROC curve for IAGsPI, in the external dataset.

The reliability and robustness of PAGsPI and IAGsPI were validated by using internal dataset (testing dataset) and external dataset. In the testing dataset, PAGsPI based on the 5 crucial PAGs was constructed as follows: PAGsPI = (0.3748 × expression of AIM2) + (0.3224 × expression of CASP5) + (0.287× expression of DFNB59) + (0.1434 × expression of GSDMC) + (0.4089 × expression of ZBP1). Evaluation of the prognostic value of PAGsPI through KM survival analysis revealed that the overall survival of KIRC patients was poor in the high-PAGsPI group (Figure 6A). Additionally, the area under the ROC curve of PAGsPI used to predict the overall survival rate of 1 year, 3 and 5 years were 0.67, 0.672, and 0.746, respectively (Figure 6B). IAGsPI was developed based on 7 key IAGs as follows: IAGsPI = (0.23533 × expression of CD27) + (0.69234 × expression of FCGR1A) − (0.05839 × expression of NLRP11) + (0.23795 × expression of PRTN3) + (0.03034 × expression of PSTPIP1) − (0.25031 × expression of TET2) + (0.25066 × expression of YJEFN3). The KM survival analysis of IAGsPI showed that the overall survival of patients in the high-IAGsPI group was unfavorable (Figure 6C). Moreover, the area under the ROC curve of IAGsPI for predicting the overall survival rate of 1 year, 3 and 5 years were 0.601, 0.607, and 0.681, respectively (Figure 6D). Similarly, in the external dataset, PAGsPI was constructed based on 5 crucial PAGs, while IAGsPI was developed based on 7 IAGs. In the external dataset, the KM survival analysis results of PAGsPI showed that patients in high-PAGsPI group had higher mortality (Figure 6E). The area under the ROC curve of PAGsPI for the 1-, 3- and 5-year overall survival prediction was 0.638, 0.662, and 0.724, respectively (Figure 6F). However, there was no difference in the survival rate of IAGsPI subgroups in the external dataset (Figure 6G). The area under the ROC curve of IAGsPI for the 1-, 3- and 5-year overall survival prediction was 0.696, 0.701 and 0.773, respectively (Figure 6H). These results showed that the PAGsPI and IAGsPI developed using the training dataset were reliable and robust models.

### 3.8 Combining Clinical Characteristics to Construct a Prognostic Nomogram

**FIGURE 7:**
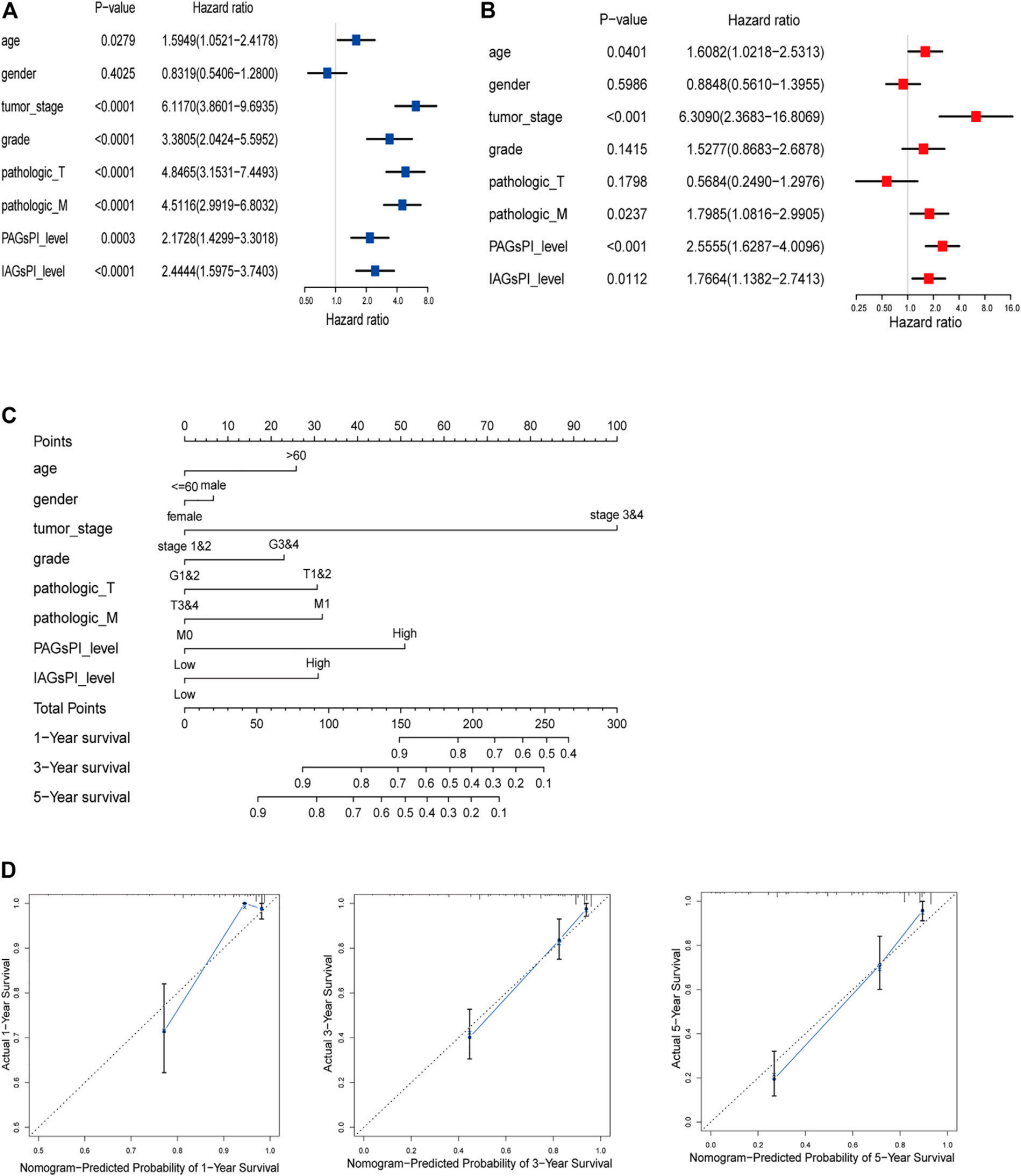
Construction of a predictive nomogram. **(A,B)** Univariate and multivariate Cox regression analyses results of age, gender, tumor stage, grade, T stage, M stage, PAGsPI and IAGsPI. **(C)** Nomogram for predicting the prognosis of 1 year, 3 and 5 years. The higher the overall score, the worse the prognosis. **(D)** The calibration curves of 1-, 3- and 5-year prognostic prediction for the nomogram. The more the blue solid line and the black dotted line coincide, the more accurate the prediction was.

Clinical characteristics are also key factors affecting prognosis. Herein, univariate and multivariate Cox regression analyses were performed on age, gender, tumor stage, grade, T stage, M stage, PAGsPI and IAGsPI, to identify the independent risk factors affecting prognosis (Figures 7A,B). The multivariate Cox regression analysis showed that age, tumor stage, M stage, PAGsPI and IAGsPI were important risk factors for the prognosis of patients with KIRC. Based on the above analyses, age, gender, tumor stage, grade, T stage, M stage, PAGsPI and IAGsPI were selected to construct a nomogram, which was convenient to comprehensively evaluate the 1-year, 3-year and 5-year prognosis of patients with KIRC (Figure 7C). The calibration curves for the nomogram of 1-year, 3-year and 5-year prognostic prediction were also drawn (Figure 7D). Moreover, the C-index for the nomogram was 0.814 (95% CI: 0.773–0.86). The results showed that the predictive ability of the nomogram was acceptable.

### 3.9 Response to Immunotherapy in Patients With Kidney Renal Clear Cell Carcinoma

**FIGURE 8:**
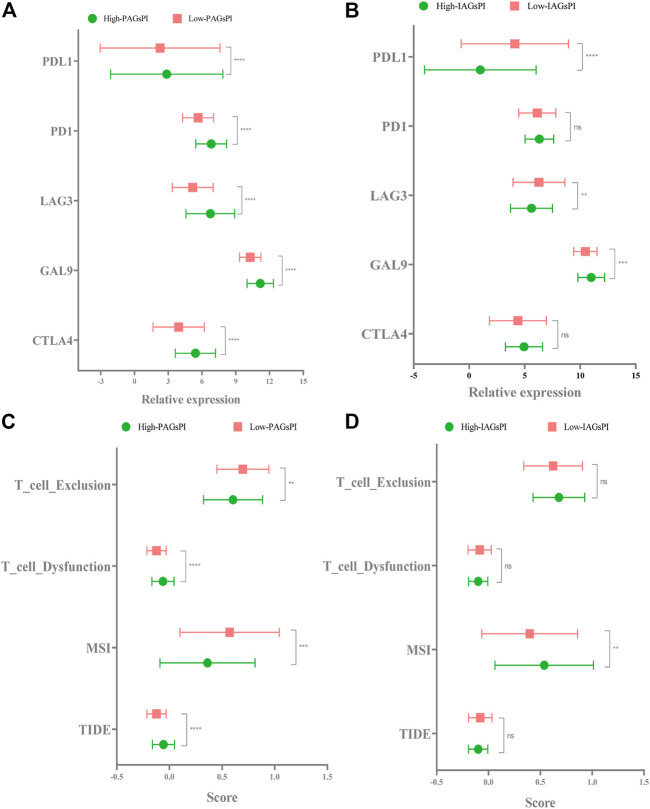
Response to immunotherapy. **(A,B)** In the training dataset, the expression of CTLA4, GAL9, LAG3, PD1 and PDL1 in the PAGsPI and IAGsPI subgroups were compared. **(C,D)** The TIDE, MSI, T cell dysfunction and T cell exclusion scores were compared in the PAGsPI and IAGsPI subgroup.

Previous studies have elucidated the correlation between pyroptosis and the expression of immune checkpoints (Shao et al., 2021). In view of the fact that the prognosis of patients who respond to immunotherapy can be improved, we compared the expression of CTLA4, GAL9, LAG3, PD1 and PDL1 in the PAGsPI and IAGsPI subgroups. In the training dataset, the high expressions of CTLA4, GAL9, LAG3, PD1 and PDL1 were observed in the high-PAGsPI group (Figure 8A). The findings also showed that there was an increase in the expression of GAL9 and a decrease in the expression of LAG3 and PDL1 in the high-IAGsPI group (Figure 8B). TIDE is a novel machine learning method based on immune gene signature to predict the response to immunotherapy, and was considered as a biomarker with more accurate prediction ability than PD1 expression or other immunotherapy prediction markers (Jiang et al., 2018). High TIDE and T cell dysfunction scores indicate that T cells with dysfunction are highly infiltrated or their recruitment is inhibited, which were closely associated with poor response to immunotherapy and prognosis. High MSI and T cell exclusion scores represented the opposite outcomes. Comparing the TIDE, MSI, T cell dysfunction and T cell exclusion scores of PAGsPI subgroup, the results demonstrated that TIDE and T cell dysfunction scores of high-PAGsPI group were higher, while the MSI and T cell exclusion scores were lower (Figure 8C). And we could find there was only MSI score increased in the high-IAGsPI group (Figure 8D). Based on these findings, we speculated patients in high-PAGsPI group were in immunosuppressive status, and may be less sensitive to immunotherapy. However, patients in high-IAGsPI group may have better response to immunotherapy, especially in anti-GAL9 therapy.

### 3.10 Biological Signatures of Differentially Expressed Genes in the Pyroptosis-Associated Genes Prognostic Index and Immune-Associated Genes Prognostic Index Subgroups

The “edgeR” package in R was used to perform the analysis of DEGs on the training dataset (high-PAGsPI vs. low-PAGsPI samples and high-IAGsPI vs low-IAGsPI samples), in order to obtain the DEGs in the PAGsPI and IAGsPI subgroups. GO analysis of DEGs in the PAGsPI subgroup revealed that these genes were not only involved in pyroptosis and immune regulation, but also related to receptor activity (Supplementary Figure S5A). In addition, KEGG analysis showed that the DEGs may modulate cytokine-cytokine receptor interaction and antigen processing and presentation (Supplementary Figure S5B). GO analysis of DEGs in the IAGsPI subgroup showed that they were mainly involved in regulatory T cell differentiation (Supplementary Figure S5C). Nonetheless, KEGG analysis did not reveal any related regulatory pathways for DEGs in the IAGsPI subgroup.

### 3.11 Validation for Pyroptosis-Associated Genes Prognostic Index and Immune-Associated Genes Prognostic Index Through an Independent Cohort

**FIGURE 9:**
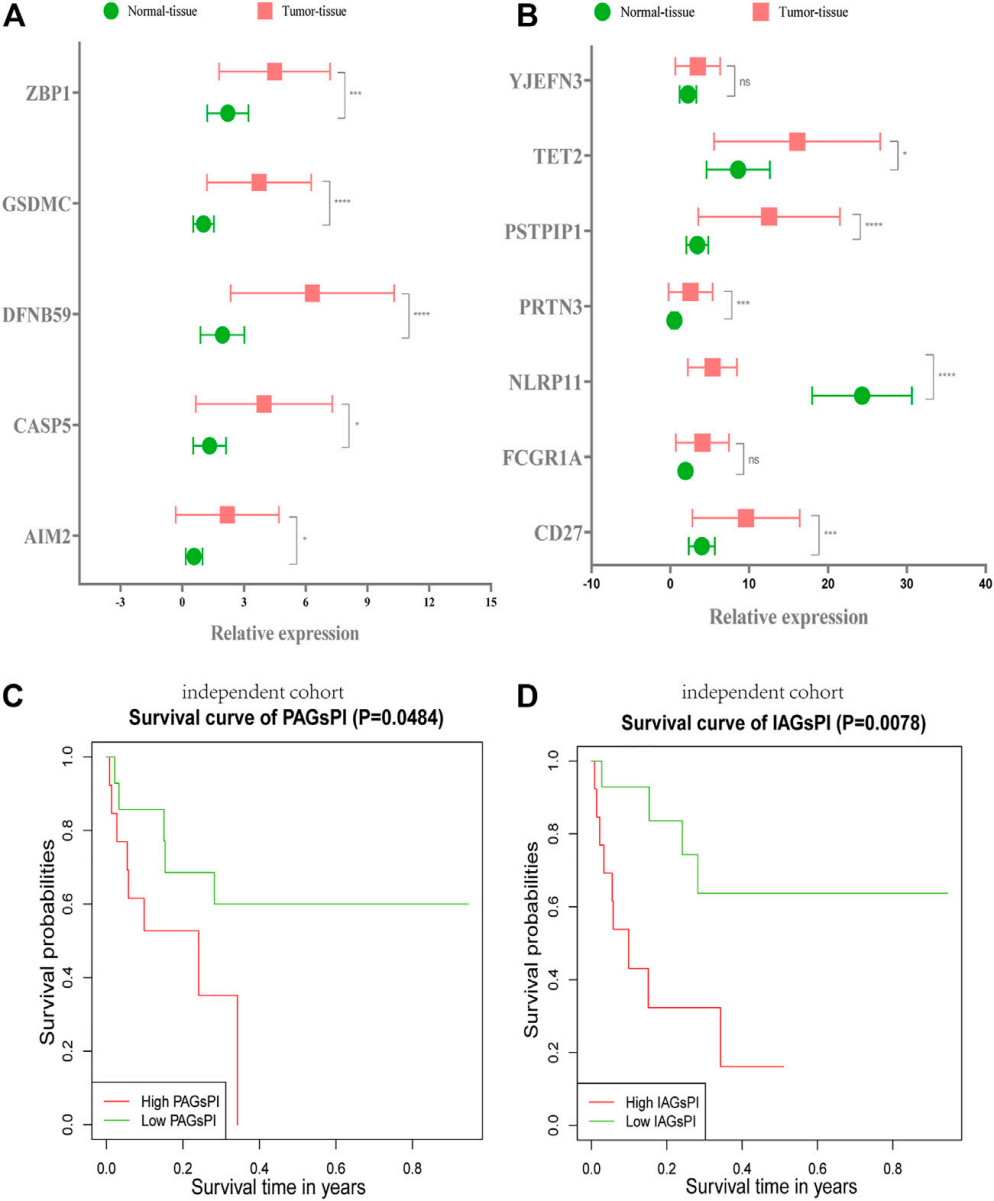
Validation of PAGsPI and IAGsPI in the independent cohort. **(A,B)** The expression level of 5 crucial PAGs and 7 key IAGs in the normal and KIRC tumor tissues. The data were shown as the means ± SD. *p < 0.05, **p < 0.01, and ***p < 0.001. **(C,D)** In the independent cohort, KM survival analysis for PAGsPI and IAGsPI.

The RNA expression levels of 5 crucial PAGs and 7 key IAGs in normal and KIRC tumor tissues were quantified through qRT–PCR, as presented in Figures 9A,B. These results were consistent with results of our analysis on the public dataset. Combined with the corresponding follow-up data, the prognosis ability of PAGsPI and IAGsPI was verified by KM survival analysis. In the independent cohort, the results displayed that the overall survival rate of patients in the high-PAGsPI group and the high-IAGsPI group was unsatisfactory (Figures 9C,D).

### 3.12 Knockdown of AIM2 Inhibits Tumor Proliferation

**FIGURE 10:**
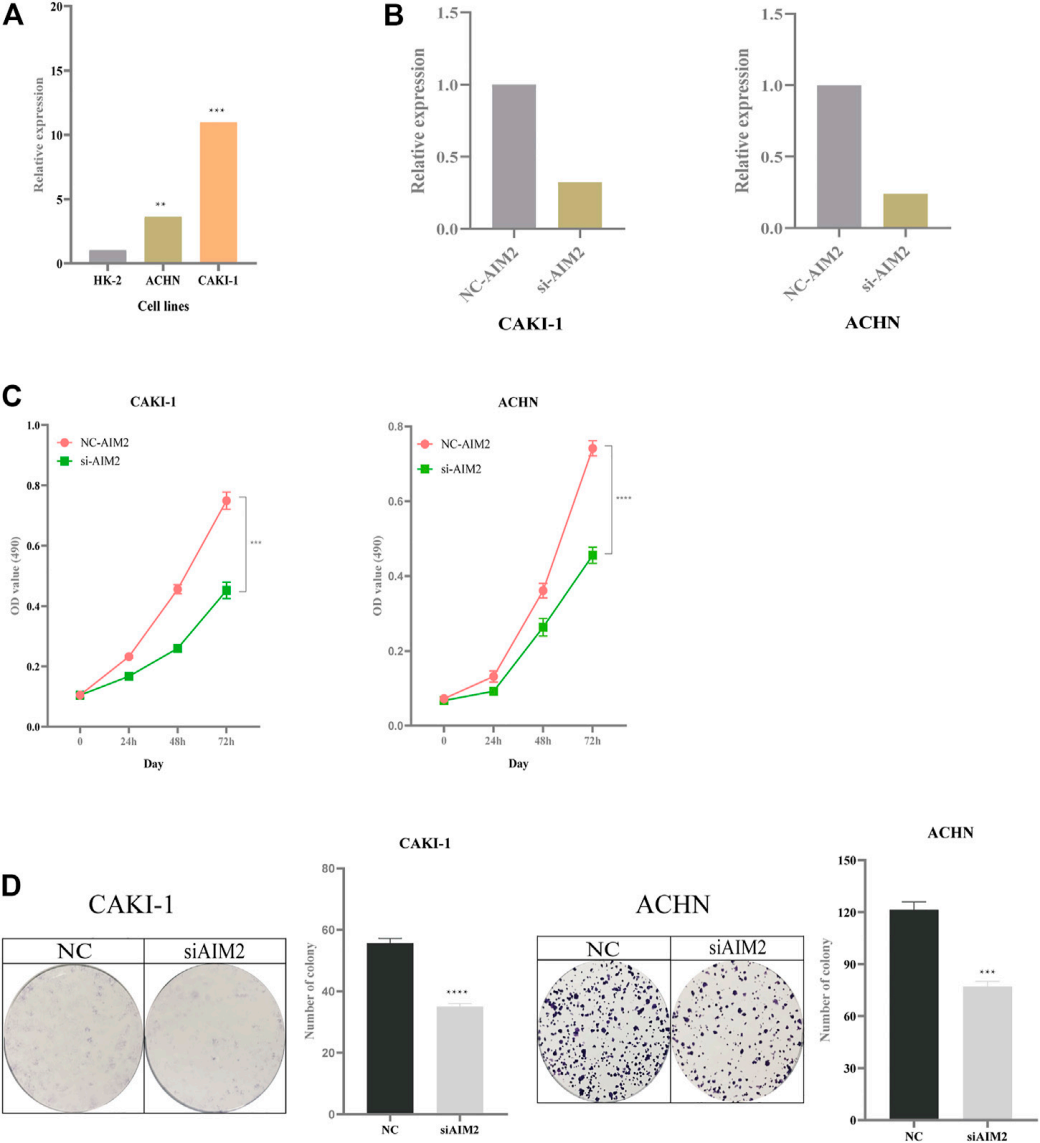
Impact of expression of AIM2 on tumor proliferation. **(A)** The expression of AIM2 in normal and tumor cell lines. **(B)** In ACHN and CAKI-1 cells, the efficiency of knockdown of AIM2 with siRNA. **(C)** MTT assays was performed in ACHN and CAKI-1 cells transfected with AIM2 siRNA and negative control siRNA. Tumor cell transfected with siRNAs were six replicates, respectively. **(D)** Colony formation assays in ACHN and CAKI-1 cells transfected with AIM2 siRNA and negative control siRNA. Tumor cell transfected with siRNAs were three replicates, respectively. The data were shown as the means ± SD. *p*-values were calculated by t-test. *p < 0.05; **p < 0.01; ***p < 0.001; ****p < 0.0001.

Compared with normal kidney cell HK-2, the expression of AIM2 increased in the CAKI-1 (increased by 10-fold) and ACHN cell lines (increased by 3.5-fold) (Figure 10A). The knockdown efficiency in CAKI-1 cell transfected with AIM2 siRNA was 68%, while that in ACHN cell transfected with AIM2 siRNA was 75% (Figure 10B). MTT assay revealed the cell viability in CAKI-1 and ACHN cell lines transfected with AIM2 siRNA was lower than that of cells transfected with negative control siRNA (Figure 10C). At 72 h, compared with cells transfected with negative control siRNA, the viability of CAKI-1 cells and ACHN cells transfected with AIM2 siRNA decreased by 39.7% (*p* < 0.001) and 38.6% (*p* < 0.0001), respectively. The knockdown of AIM2 suppressed the formation of colony in the CAKI-1 and ACHN cell lines (Figure 10D). The clone number of knockdown cells was less than that of negative control cells. On the 7th day, compared with the cells transfected with negative control siRNA, the clone number of CAKI-1 and ACHN cells transfected with AIM2 siRNA was reduced by 37.1% (*p* < 0.0001) and 36.5% (*p* = 0.0002), respectively. These results suggested that the expression levels of AIM2 may influence the proliferation of CAKI-1 and ACHN cells.

## 4 Discussion

Pyroptosis is an inflammasome-mediated form of programmed cell death that has received tremendous attention for its distinct antitumor mechanisms which are different from those of apoptosis (Xia et al., 2019; Ju et al., 2021). It was also shown that the transcription factor, p53 promoted the pyroptosis of non-small-cell lung cancer cells, suppressing tumor growth (Zhang T et al., 2019). Moreover, the antitumor effect of chemotherapeutic drugs can be exerted by inducing tumor-cell pyroptosis. According to a previous study, chemotherapy drugs paclitaxel and cisplatin were beneficial to patients with lung cancer since they could activate caspase-3/GSDME to promote pyroptosis of cancer cells (Zhang C-C et al., 2019). Furthermore, pyroptosis was characterized by the release of the inflammatory cytokines, IL-1β and IL-18 (Fang et al., 2020). On the one hand, IL-1β and IL-18 were correlated with tumor growth, invasion, angiogenesis and metastasis (Krelin et al., 2007; Ju et al., 2021). On the other hand, they have been proven to be able to regulate the functions of immune cells, such as cytotoxic T cells, neutrophils and NK cells, to exert their antitumor effects (Haabeth et al., 2016; Kaplanski, 2018; Ju et al., 2021). In bladder cancer, pyroptosis has been proven to affect the remodeling of tumor microenvironment (TME) and activation of pyroptosis attributes to infiltration of immune cells and to enhance the efficiency of immunotherapy (Chen et al., 2021a). These studies on pyroptosis in cancer cells provide novel insights for cancer cell death, especially in anti-apoptosis tumors, and tumor-immune response.

The hub pyroptosis-associated molecules related to the prognosis of KIRC patients remain unclear. In this study, Cox and LASSO regression analysis identified 2 PAGs which can be considered to be independent prognostic factors, and constructed and validated PAGsPI. Patients with high expression of AIM2 and DFNB59 had a poor prognosis. AIM2 can be activated by endogenous or pathogen-derived double-stranded DNA (dsDNA), which induces cell pyroptosis by the canonical inflammasome pathway, in which caspase-1 was the important effector (Fang et al., 2020). In cervical cancer cells, SIRT1 can suppress pyroptosis by interfering with the transcription of AIM2 (So et al., 2018). The expression of AIM2 was linked with the status of TME. AIM2 exerts an immunosuppressive effect within the melanoma microenvironment (Fukuda et al., 2021). It can regulate the production of IL-1β and IL-18 to influence T reg cell accumulation and tumor growth. In addition, T cell reactivity against AIM2 has also been found in melanoma, indicating that AIM2-derived peptides can serve as relevant targets for immunomonitoring (Zhu et al., 2021). In renal cancer, AIM2 can induce the transformation of anti-inflammatory M2 to pro-inflammatory M1 by enhancing the inflammasome pathway, thus inhibiting tumor progression (Chai et al., 2021). And in our study, we also clarified the relationship between the expression of AIM2 and infiltration of immune cells like B cells, CD8^+^ T cells, CD4^+^ T cells, macrophage, neutrophil and dendritic cells. Therefore, AIM2 not only can induce pyroptosis but also affect the status of TME to modulate tumor behaviors. DFNB59 (also known as PJVK) belongs to the Gasdermin (GSDM) family and plays an important role in modulating cell pyroptosis (Broz et al., 2020). However, the mechanism by which DFNB59 induces cell pyroptosis remains to be explored (Broz et al., 2020). PAGsPI can be used to assess the impact of crucial PAGs expression on the prognosis of patients with KIRC. In this study, we compared the infiltration of immune cells and the related immune pathways in PAGsPI subgroup. The results revealed that the infiltration of CD8^+^ T cells and regulatory T cells in high-PAGsPI group was higher. The results also showed that T cell co-stimulation signaling pathway existed in the low-PAGsPI group. These findings were consistent with previous studies, which have demonstrated the characteristics of KIRC were abundant infiltration of immune cells, especially in T cells (Díaz-Montero et al., 2020). Therefore, inducing tumor cell pyroptosis and enhancing immune killing may enhance the effects of antitumor mechanisms. In view of the important role of the tumor microenvironment in pyroptosis of tumor cells, the biological signatures of IAGs highly associated with PAGs were investigated to elucidate their impact on prognosis. The immune genes of CD27, FCGR1A, NLRP11, PRTN3, PSTPIP1, TET2 and YJEFN3 were considered to be important molecules closely linked to prognosis, and the IAGsPI developed based on them was an important prognosis model for pyroptosis. NLRP11 and PRTN3 were identified as important independent immune genes that affect overall survival. Yu et al. (2017) have found variant of NLRP11 was closely correlated with HBV-associated hepatocellular carcinoma, in which the specific mechanism needs further study. High expression of PRTN3 has been proved to be associated with poor prognosis in pancreatic cancer patients (Hu et al., 2019). The dual prognostic indices which were validated were capable of comprehensively and accurately assessing the prognosis of patients with KIRC in terms of pyroptosis and immunity. Additionally, immunotherapy has been shown to have numerous advantages in cancer treatment; However, only a fraction of patients can benefit from it (Yang, 2015). CD8^+^ T effectors were the major subtype of CD8^+^ T cells to exert anti-tumor. CD8^+^ T effectors can be used to estimate the patients’ response to immunotherapy (Lees, 2020). The immune checkpoint inhibitor score (IMS) scoring system based on CD8^+^ T effector and immune checkpoint signatures can better predict the response to immunotherapy and can estimate the prognosis of patients with bladder cancer (Chen et al., 2021b). Thus, the infiltration level of CD8^+^ T effectors may partly determine the final events of the tumor. Although the infiltration of CD 8 + T cells was more in the high PAGsPI group, and the expression of CTLA 4, LAG 3, GAL 9, PD 1 and PDL 1 were increased, the infiltration level of CD8^+^ T effectors was lower and the TIDE and T cell dysfunction scores were higher, which indicated that CD8^+^ T cells had weak anti-tumor effect. Therefore, patients in the high-PAGsPI group may be less sensitive to immunotherapy. In addition, although there was no statistical significance in TIDE and T cell dysfunction scores in IAGsPI subgroup, we can observe the scores were lower in the high-IAGsPI. Combined with MSI score, we may speculate that patients in high-IAGsPI group could obtain a better immunotherapy effect. Therefore, immunotherapy may play a 1 + 1 greater than 2 effect for patients in both the low-PAGsPI and high-IAGsPI groups. The nomogram can comprehensively predict the prognosis of patients with KIRC from the aspects of pyroptosis, immune and clinical characteristics. The validation of PAGsPI and IAGsPI in the independent cohort indicated that these models had better prediction performance. Here, we found expression of AIM2 may affect the proliferation of tumor cells, but the specific regulation pathway needs further study. However, Chai et al. (2018) have found the expression of AIM2 decreased in the 786-O and OSRC-2 cells, which may exert antitumor role in renal cell carcinoma. In addition, they also found that the expression of AIM2 increased in the ACHN cell. Thus, the expression of AIM2 in different cells may have different effects on tumor cells.

While the present study uncovered some insightful findings, it had a number of limitations. First, the pyroptosis-associated genes were retrieved from current studies on pyroptosis. With the development of research of cell death forms, more molecules associated with pyroptosis will be discovered. Second, given that it is difficult to establish the presence of lymph node metastasis in many patients, the effect of the expression of AIM2, DFNB59, NLRP11 and PRTN3 on T staging was not fully investigated. Therefore, this factor was not included in the nomogram model. Third, it is not clear how AIM2 and DFNB59 regulate pyroptosis of KIRC cells. Therefore, further basic experiments involving these molecules are required to investigate the downstream molecular mechanisms.

## 5 Conclusion

In this study, 5 crucial PAGs were identified and PAGsPI was developed and validated, and their expression levels in different TNM stages were analyzed. Moreover, the infiltration of immune cells in the PAGsPI subgroup was investigated. 7 key IAGs highly correlated with the pyroptosis genes were identified due to immune response during cell pyroptosis. Additionally, IAGsPI was developed and its prognostic value validated. The response of patients in different subgroups of PAGsPI and IAGsPI to immunotherapy was also assessed. Furthermore, the anti-pyroptotic regulatory pathways leading to tumor progression were explored through functional analysis. A nomogram can also be used to predict prognosis. Finally, qRT-PCR was used to quantify the expression of the 5 crucial PAGs and 7 key IAGs in normal and KIRC tumor tissues. The prognostic values of PAGsPI and IAGsPI were further validated in an independent cohort. These findings not only provide a novel understanding of the development and progression of tumors, but also provide a potential new antitumor strategy for pyroptosis. PAGsPI and IAGsPI could be regarded as potential biomarkers to predict prognosis of patients with KIRC.

## Data Availability

The original contributions presented in the study are included in the article/Supplementary Material, further inquiries can be directed to the corresponding authors.

## Abbreviations

DEGs, differentially expressed genes; DEIAGs, differentially expressed immune-associated genes; DEPAGs, differentially expressed pyroptosis-associated genes; GO, Gene Ontology; IAGs, immune-associated genes; IAGsPI, immune-associated genes prognostic index; C-index, the concordance index; KEGG, Kyoto Encyclopedia of Genes and Genomes; KIRC, kidney renal clear cell carcinoma; LASSO, the Least Absolute Shrinkage and Selection Operator; PAGs: pyroptosis-associated genes; PAGsPI, pyroptosis-associated genes prognostic index; qRT–PCR, Quantitative reverse transcription–polymerase chain reaction; ROC, the receiver operating characteristic; ssGSEA, single sample gene set enrichment analysis; TIDE, Tumor Immune Dysfunction and Exclusion; TIMER, Tumor IMmune Estimation Resource; TIP, Tracking Tumor Immunophenotype.
