# Supplementary material for: Generation and Analysis of Pyroptosis-Based and Immune-Based Signatures for Kidney Renal Clear Cell Carcinoma Patients, and Cell Experiment
Source: Front Genet. 2022 Feb 24;13:809794. doi: 10.3389/fgene.2022.809794 (PMC8908022; doi:10.3389/fgene.2022.809794)
Supplement: Supplementary file 1 [file DataSheet4.DOCX]

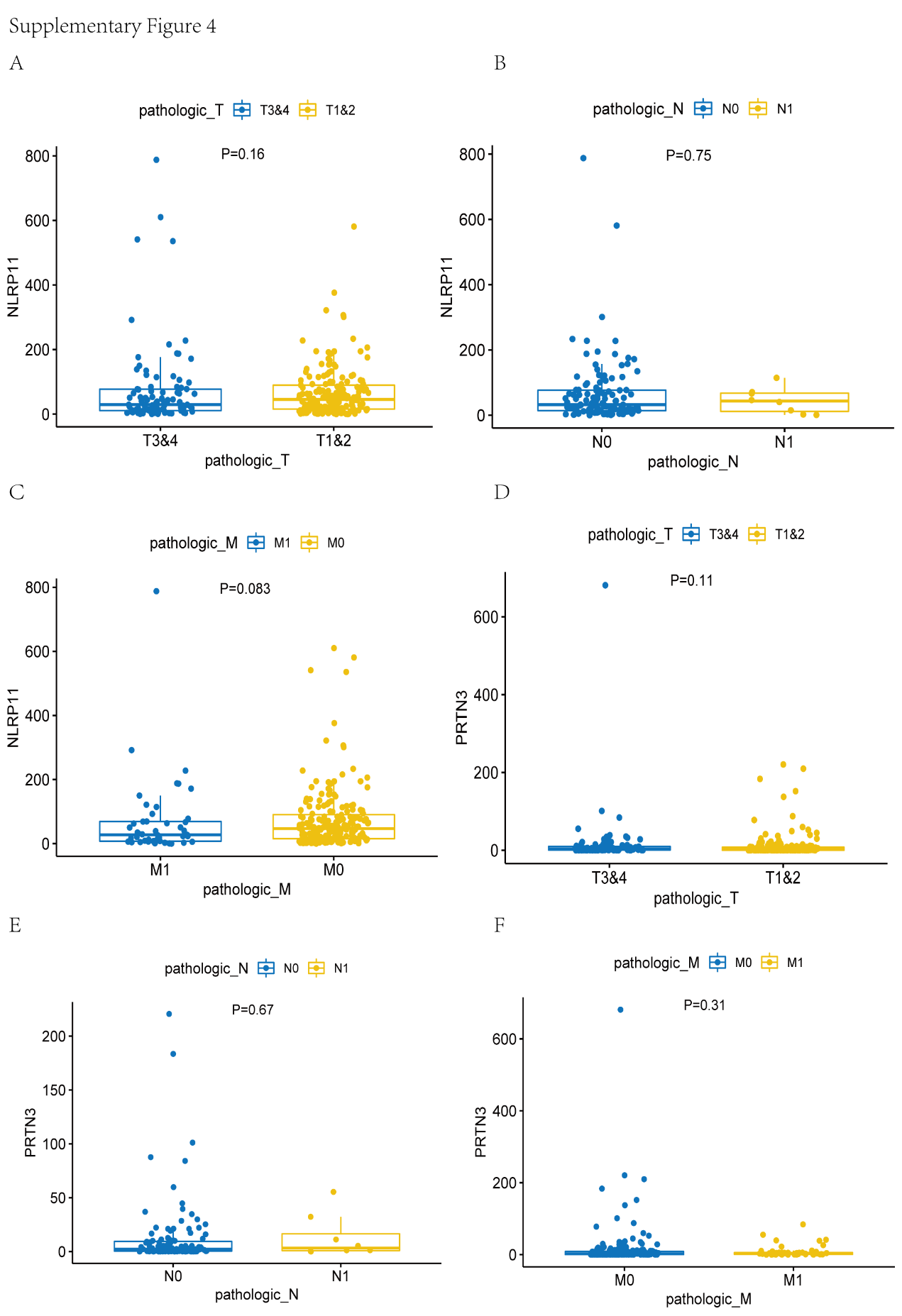


Supplementary Figure 4: The expression of the 2 independent IAGs in different TNM staging. (A-C) There were no expressed differences for NLRP11 in different TNM staging. (D-F) There were no expressed differences for PRTN3 in different TNM staging.
