# Supplementary material for: Generation and Analysis of Pyroptosis-Based and Immune-Based Signatures for Kidney Renal Clear Cell Carcinoma Patients, and Cell Experiment
Source: Front Genet. 2022 Feb 24;13:809794. doi: 10.3389/fgene.2022.809794 (PMC8908022; doi:10.3389/fgene.2022.809794)
Supplement: Supplementary file 2 [file Table1.DOCX]

Supplementary Table 1: Pyroptosis-associated genes

Genes Full-names Ref

AIM2 Absent in melanoma 2 1

CASP1 cysteine-aspartic acid protease-1 1

CASP3 cysteine-aspartic acid protease-3 2

CASP4 cysteine-aspartic acid protease-4 1

CASP5 cysteine-aspartic acid protease-5 1

CASP6 cysteine-aspartic acid protease-6 3

CASP8 cysteine-aspartic acid protease-8 3

GPX4 glutathione peroxidase 4 4

GSDMA gasdermin A 5

GSDMB gasdermin B 5

GSDMC gasdermin C 5

GSDMD gasdermin D 5

GSDME gasdermin E 5

PJVK Pejvakin 5

IL18 interleukin 18 1

IL1B interleukin 1 beta 1

IL6 interleukin 6 6

NLRC4 NLR family CARD domain containing 4 7

NLRP1 NLR family pyrin domain containing 1 7

NLRP3 NLR family pyrin domain containing 3 7

NLRP6 NLR family pyrin domain containing 6 7

NLRP9 NLR family pyrin domain containing 9 7

NLRP12 NLR family pyrin domain containing 12 8

NEK7 NIMA-related kinase-7 1

NOD1 nucleotide binding oligomerization domain containing 1 7

NOD2 nucleotide binding oligomerization domain containing 2 7

PLCG1 phospholipase C gamma 1 4

PYCARD PYD and CARD domain containing 7

MYD88 Myeloid Differentiation Primary Response 88 7

TNF tumor necrosis factor 7

HMGB1 High Mobility Group Box 1 9

MEFV MEFV Innate Immuity Regulator, Pyrin 1

ZBP1 Z-DNA Binding Protein 1 3

NAIP NLR Family Apoptosis Inhibitory Protein 7

IFI16 Interferon Gamma Inducible Protein 16 10

RIG-I/DDX58 DExD/H-Box Helicase 58 1

STING stimulator of interferon response CGAMP interactor 1 1

GZMA Granzyme A 11

GZMB Granzyme B 12

TAK1/ MAP3K7 Mitogen-Activated Protein Kinase Kinase Kinase 7 7

RIPK1 Receptor Interacting Serine/Threonine Kinase 1 7

RIPK3 Receptor Interacting Serine/Threonine Kinase 3 7

FADD Fas Associated Via Death Domain 7

P2X7 purinergic receptor P2X 7 7

DPP8 Dipeptidyl Peptidase 8 13

DPP9 Dipeptidyl Peptidase 9 13

SCGB3A2 Secretoglobin Family 3A Member 2 14

SDC1 Syndecan 1 14

TOM20 tanslocase of outer mitochondrial membrane 20 15

BAX BCL2 Associated X, Apoptosis Regulator 7, 15

PELP1 Proline, Glutamate and Leucine Rich Protein 1 16

CARD8 Caspase Recruitment Domain Family Member 8 13

PD-L1 Programmed Death Ligand 1 17

STAT3 Signal Transducer And Activator Of Transcription 3 18

PANX1 Pannexin 1 7

SIRT1 Sirtuin 1 19

NFKB1/NF-kB Nuclear Factor Kappa B Subunit 1 7

P53 Tumor Protein P53 20

APAF1 Apoptotic Peptidase Activating Factor 1 21

LXRbeta/NR1H2 Nuclear Receptor Subfamily 1 Group H Member 2 22

JNK/MAPK8 Mitogen-Activated Protein Kinase 8 23

PKR Protein kinase R 24

CIAP1/BIRC2 Baculoviral IAP Repeat Containing 2 25

CIAP2/ BIRC3 Baculoviral IAP Repeat Containing 3 25

TLR2 toll like receptor 2 26

TLR4 toll like receptor 4 26

DEC2/ BHLHE41 Basic Helix-Loop-Helix Family Member E41 27

TXNIP Thioredoxin Interacting Protein 28

IRF2 interferon regulatory factor 2 29

IRF1 interferon regulatory factor 1 30

CD73/NT5E 5'-Nucleotidase Ecto 31

PI3K phosphatidylinositol 3-kinase, putative 31

AKT AKT serine/threonine kinase 1 31

FOXO1 forkhead box O1 31

P38-MAPK/MAPK14 Mitogen-Activated Protein Kinase 14 32

VDR Vitamin D Receptor 33

HDAC6 histone deacetylase 6 34

SYK spleen associated tyrosine kinase 35

BDNF Brain-derived neurotrophic factor 36

KLF2 Kruppel-like family of transcription factor 2 36

HK1 Hexokinase 1 36

RP105/CD180 CD180 Molecule 37

NLRX1 NLR family member X1 38

ASK apoptosis signal-regulating kinase 7

TRAF6 TNF receptor associated factor 6 7

BAK BCL2 Antagonist/Killer 7

References

1. Man SM, Karki R, Kanneganti T-D. Molecular mechanisms and functions of pyroptosis, inflammatory caspases and inflammasomes in infectious diseases. Immunol Rev. 2017;277(1):61-75.

2. Jiang M, Qi L, Li L, Li Y. The caspase-3/GSDME signal pathway as a switch between apoptosis and pyroptosis in cancer. Cell Death Discov. 2020;6:112.

3. Zheng M, Kanneganti T-D. The regulation of the ZBP1-NLRP3 inflammasome and its implications in pyroptosis, apoptosis, and necroptosis (PANoptosis). Immunol Rev. 2020;297(1):26-38.

4. Kang R, Zeng L, Zhu S, Xie Y, Liu J, Wen Q, et al. Lipid Peroxidation Drives Gasdermin D-Mediated Pyroptosis in Lethal Polymicrobial Sepsis. Cell Host Microbe. 2018;24(1).

5. Feng S, Fox D, Man SM. Mechanisms of Gasdermin Family Members in Inflammasome Signaling and Cell Death. J Mol Biol. 2018;430(18 Pt B):3068-80.

6. Wu X-Y, Li K-T, Yang H-X, Yang B, Lu X, Zhao L-D, et al. Complement C1q synergizes with PTX3 in promoting NLRP3 inflammasome over-activation and pyroptosis in rheumatoid arthritis. J Autoimmun. 2020;106:102336.

7. Xue Y, Enosi Tuipulotu D, Tan WH, Kay C, Man SM. Emerging Activators and Regulators of Inflammasomes and Pyroptosis. Trends Immunol. 2019;40(11):1035-52.

8. Chen H, Deng Y, Gan X, Li Y, Huang W, Lu L, et al. NLRP12 collaborates with NLRP3 and NLRC4 to promote pyroptosis inducing ganglion cell death of acute glaucoma. Mol Neurodegener. 2020;15(1):26.

9. Broz P, Pelegrín P, Shao F. The gasdermins, a protein family executing cell death and inflammation. Nat Rev Immunol. 2020;20(3):143-57.

10. Song Y, Wu X, Xu Y, Zhu J, Li J, Zou Z, et al. HPV E7 inhibits cell pyroptosis by promoting TRIM21-mediated degradation and ubiquitination of the IFI16 inflammasome. Int J Biol Sci. 2020;16(15):2924-37.

11. Zhou Z, He H, Wang K, Shi X, Wang Y, Su Y, et al. Granzyme A from cytotoxic lymphocytes cleaves GSDMB to trigger pyroptosis in target cells. Science. 2020;368(6494).

12. Zhang Z, Zhang Y, Xia S, Kong Q, Li S, Liu X, et al. Gasdermin E suppresses tumour growth by activating anti-tumour immunity. Nature. 2020;579(7799):415-20.

13. Johnson DC, Taabazuing CY, Okondo MC, Chui AJ, Rao SD, Brown FC, et al. DPP8/DPP9 inhibitor-induced pyroptosis for treatment of acute myeloid leukemia. Nat Med. 2018;24(8):1151-6.

14. Yokoyama S, Nakayama S, Xu L, Pilon AL, Kimura S. Secretoglobin 3A2 eliminates human cancer cells through pyroptosis. Cell Death Discov. 2021;7(1):12.

15. Zhou B, Zhang J-Y, Liu X-S, Chen H-Z, Ai Y-L, Cheng K, et al. Tom20 senses iron-activated ROS signaling to promote melanoma cell pyroptosis. Cell Res. 2018;28(12):1171-85.

16. Wang L, Li K, Lin X, Yao Z, Wang S, Xiong X, et al. Metformin induces human esophageal carcinoma cell pyroptosis by targeting the miR-497/PELP1 axis. Cancer Lett. 2019;450:22-31.

17. Hou J, Zhao R, Xia W, Chang C-W, You Y, Hsu J-M, et al. PD-L1-mediated gasdermin C expression switches apoptosis to pyroptosis in cancer cells and facilitates tumour necrosis. Nat Cell Biol. 2020;22(10):1264-75.

18. Yao R, Chen Y, Hao H, Guo Z, Cheng X, Ma Y, et al. Pathogenic effects of inhibition of mTORC1/STAT3 axis facilitates Staphylococcus aureus-induced pyroptosis in human macrophages. Cell Commun Signal. 2020;18(1):187.

19. Zheng Z, Bian Y, Zhang Y, Ren G, Li G. Metformin activates AMPK/SIRT1/NF-κB pathway and induces mitochondrial dysfunction to drive caspase3/GSDME-mediated cancer cell pyroptosis. Cell Cycle. 2020;19(10):1089-104.

20. Zhang T, Li Y, Zhu R, Song P, Wei Y, Liang T, et al. Transcription Factor p53 Suppresses Tumor Growth by Prompting Pyroptosis in Non-Small-Cell Lung Cancer. Oxid Med Cell Longev. 2019;2019:8746895.

21. Xu W, Che Y, Zhang Q, Huang H, Ding C, Wang Y, et al. Apaf-1 Pyroptosome Senses Mitochondrial Permeability Transition. Cell Metab. 2021;33(2).

22. Derangère V, Chevriaux A, Courtaut F, Bruchard M, Berger H, Chalmin F, et al. Liver X receptor β activation induces pyroptosis of human and murine colon cancer cells. Cell Death Differ. 2014;21(12):1914-24.

23. Yu J, Li S, Qi J, Chen Z, Wu Y, Guo J, et al. Cleavage of GSDME by caspase-3 determines lobaplatin-induced pyroptosis in colon cancer cells. Cell Death Dis. 2019;10(3):193.

24. Hett EC, Slater LH, Mark KG, Kawate T, Monks BG, Stutz A, et al. Chemical genetics reveals a kinase-independent role for protein kinase R in pyroptosis. Nat Chem Biol. 2013;9(6):398-405.

25. Chen Z, Xu G, Wu D, Wu S, Gong L, Li Z, et al. Lobaplatin induces pyroptosis through regulating cIAP1/2, Ripoptosome and ROS in nasopharyngeal carcinoma. Biochem Pharmacol. 2020;177:114023.

26. Tian L, Yan J, Li K, Zhang W, Lin B, Lai W, et al. Ozone exposure promotes pyroptosis in rat lungs via the TLR2/4-NF-κB-NLRP3 signaling pathway. Toxicology. 2021;450:152668.

27. Oka S, Li X, Sato F, Zhang F, Tewari N, Kim I-S, et al. A deficiency of Dec2 triggers periodontal inflammation and pyroptosis. J Periodontal Res. 2021;56(3):492-500.

28. Heo MJ, Kim TH, You JS, Blaya D, Sancho-Bru P, Kim SG. Alcohol dysregulates miR-148a in hepatocytes through FoxO1, facilitating pyroptosis via TXNIP overexpression. Gut. 2019;68(4):708-20.

29. Kayagaki N, Lee BL, Stowe IB, Kornfeld OS, O'Rourke K, Mirrashidi KM, et al. IRF2 transcriptionally induces expression for pyroptosis. Sci Signal. 2019;12(582).

30. Karki R, Sharma BR, Lee E, Banoth B, Malireddi RKS, Samir P, et al. Interferon regulatory factor 1 regulates PANoptosis to prevent colorectal cancer. JCI Insight. 2020;5(12).

31. Xu S, Wang J, Zhong J, Shao M, Jiang J, Song J, et al. CD73 alleviates GSDMD-mediated microglia pyroptosis in spinal cord injury through PI3K/AKT/Foxo1 signaling. Clin Transl Med. 2021;11(1):e269.

32. Chen S, Zuo Y, Huang L, Sherchan P, Zhang J, Yu Z, et al. The MC receptor agonist RO27-3225 inhibits NLRP1-dependent neuronal pyroptosis via the ASK1/JNK/p38 MAPK pathway in a mouse model of intracerebral haemorrhage. Br J Pharmacol. 2019;176(9):1341-56.

33. Jiang S, Zhang H, Li X, Yi B, Huang L, Hu Z, et al. Vitamin D/VDR attenuate cisplatin-induced AKI by down-regulating NLRP3/Caspase-1/GSDMD pyroptosis pathway. J Steroid Biochem Mol Biol. 2021;206:105789.

34. Xu S, Chen H, Ni H, Dai Q. Targeting HDAC6 attenuates nicotine-induced macrophage pyroptosis via NF-κB/NLRP3 pathway. Atherosclerosis. 2021;317:1-9.

35. Li Y, Song W, Tong Y, Zhang X, Zhao J, Gao X, et al. Isoliquiritin ameliorates depression by suppressing NLRP3-mediated pyroptosis via miRNA-27a/SYK/NF-κB axis. J Neuroinflammation. 2021;18(1):1.

36. Jin H, Zhu Y, Wang X-D, Luo E-F, Li Y-P, Wang B-L, et al. BDNF corrects NLRP3 inflammasome-induced pyroptosis and glucose metabolism reprogramming through KLF2/HK1 pathway in vascular endothelial cells. Cell Signal. 2021;78:109843.

37. Guo X, Hu S, Liu J-J, Huang L, Zhong P, Fan Z-X, et al. Piperine protects against pyroptosis in myocardial ischaemia/reperfusion injury by regulating the miR-383/RP105/AKT signalling pathway. J Cell Mol Med. 2021;25(1):244-58.

38. Zhu X, Wu T, Chi Y, Ge Y, Jiao Y, Zhu F, et al. MicroRNA-195 suppresses enterovirus A71-induced pyroptosis in human neuroblastoma cells through targeting NLRX1. Virus Res. 2021;292:198245.
