# Supplementary material for: Generation and Analysis of Pyroptosis-Based and Immune-Based Signatures for Kidney Renal Clear Cell Carcinoma Patients, and Cell Experiment
Source: Front Genet. 2022 Feb 24;13:809794. doi: 10.3389/fgene.2022.809794 (PMC8908022; doi:10.3389/fgene.2022.809794)
Supplement: Supplementary file 4 [file DataSheet5.DOCX]

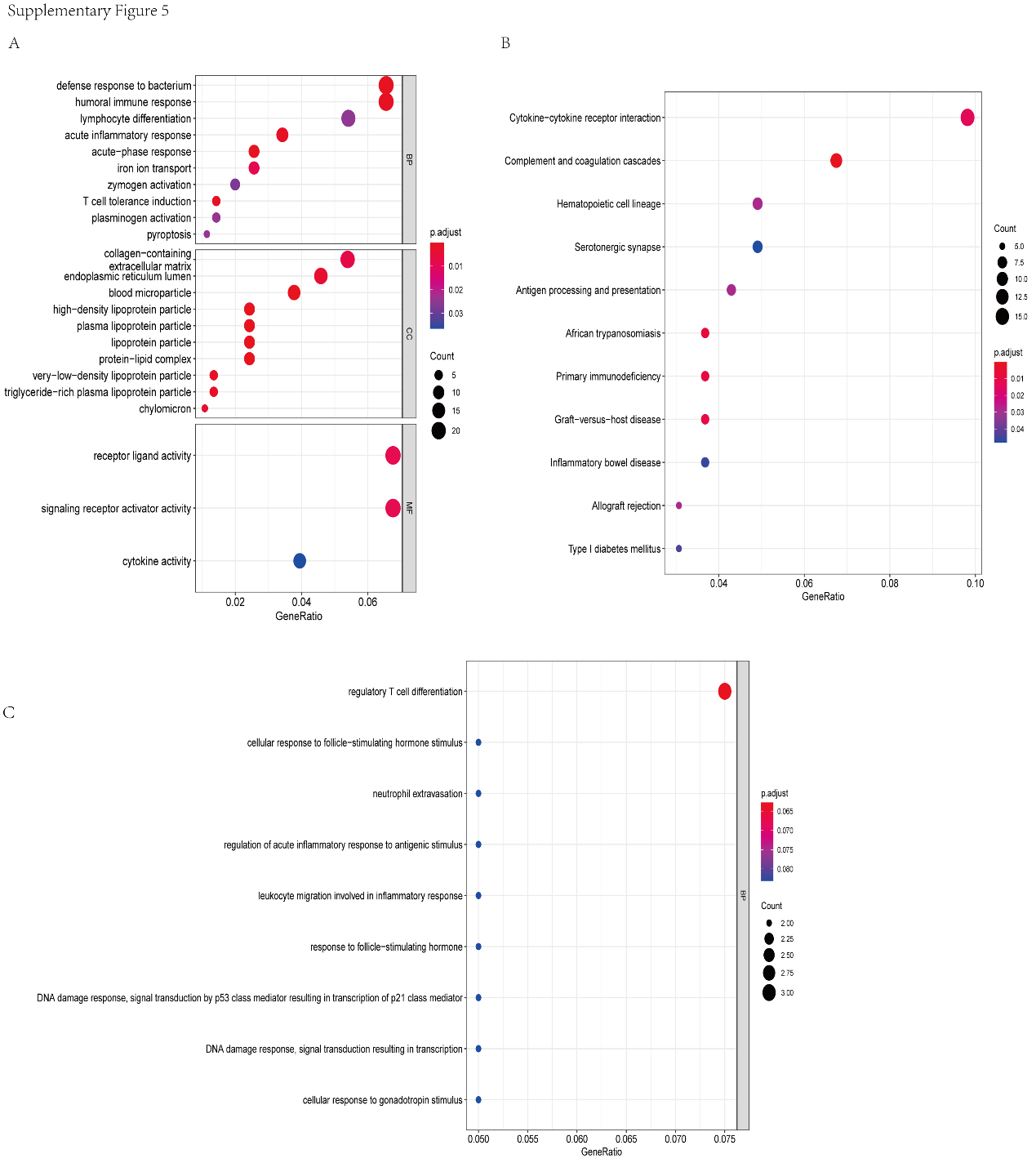


Supplementary Figure 5: GO and KEGG analyses. (A) GO analysis for DEGs between PAGsPI subgroups, shown by dot plot. (B) KEGG analysis for DEGs between PAGsPI subgroups, illustrated by dot plot. (C) GO analysis for DEGs between IAGsPI subgroups, displayed by dot plot.
