# Supplementary material for: Generation and Analysis of Pyroptosis-Based and Immune-Based Signatures for Kidney Renal Clear Cell Carcinoma Patients, and Cell Experiment
Source: Front Genet. 2022 Feb 24;13:809794. doi: 10.3389/fgene.2022.809794 (PMC8908022; doi:10.3389/fgene.2022.809794)
Supplement: Supplementary file 5 [file Table2.DOCX]

Supplementary Table 2: Clinical characteristics of training cohort and testing cohort

characteristics training cohort testing cohort χ^2^ whole TCGA cohort

age

<=60/>60 127/144 143/127 P = 0.33 270/271

gender

male/female 176/95 179/91 P = 0.41 355/186

stage

ⅰ/ⅱ/ⅲ/ⅳ/not report 133/34/52/50/2 141/24/74/33/1 P = 0.996 274/58/126/83/3

grade

G1/G2/G3/G4/GX 5/114/111/38/1 9/120/99/48/4 P = 0.94 14/234/210/78/5

pathologic_T

T1/T2/T3/T4 135/42/87/7 145/29/92/4 P = 0.998 280/71/179/31

pathologic_N

N0/N1/NX 123/8/140 120/8/142 P = 0.93 243/16/282

pathologic_M

M0/M1/MX 206/48/17 224/30/14 P = 0.69 430/78/31
