# Supplementary material for: Generation and Analysis of Pyroptosis-Based and Immune-Based Signatures for Kidney Renal Clear Cell Carcinoma Patients, and Cell Experiment
Source: Front Genet. 2022 Feb 24;13:809794. doi: 10.3389/fgene.2022.809794 (PMC8908022; doi:10.3389/fgene.2022.809794)
Supplement: Supplementary file 6 [file Table3.DOCX]

Supplementary Table 3: Immune genes correlated with pyroptosis genes

Pyroptosis genes Immune genes Pearson Correlation P value

GZMB HIF1AN 0.57 5.94e^-25^

GZMB PQBP1 0.53 2.26e^-21^

GZMB NLRP11 0.21 0.00057

GZMB PRKDC 0.43 6.26e^-14^

GZMB GLI1 0.13 0.03622

GZMB DEFA6 0.16 0.00933

NFKB1 PDCD1LG2 0.64 1.06e^-32^

NFKB1 PRKDC -0.12 0.04133

NFKB1 HIF1AN 0.46 9.33e^-16^

NFKB1 PQBP1 0.34 8.72e^-09^

NFKB1 VENTX -0.41 3.17e^-12^

NFKB1 LTB4R -0.49 1.21e^-17^

NFKB1 YJEFN3 -0.29 1.05e^-06^

NFKB1 LAT -0.36 1.47e^-09^

NFKB1 F11 -0.25 3.30e^-05^

NFKB1 USP2 -0.25 2.72e^-05^

NFKB1 F2RL3 -0.37 3.76e^-10^

NFKB1 CTNNAL1 -0.49 1.55e^-17^

NFKB1 KITLG -0.4 7.54e^-12^

NFKB1 CD1D -0.54 3.27e^-22^

NFKB1 VEGFA -0.51 2.82e^-19^

NFKB1 MAP3K14 -0.51 1.08e^-19^

NFKB1 TRADD -0.59 2.08e^-26^

NFKB1 OAS1 -0.5 1.66e^-18^

NFKB1 ANXA4 -0.53 4.08e^-21^

NFKB1 TLR3 -0.52 4.33e^-20^

NFKB1 PELI2 -0.54 1.74e^-21^

NFKB1 TRIM9 -0.47 1.80e^-16^

NFKB1 SELE -0.28 2.26e^-06^

NFKB1 C7 -0.28 2.00e^-06^

NFKB1 CXCL12 -0.43 2.26e^-13^

NFKB1 TLR6 -0.42 5.81e^-13^

NFKB1 IFNG -0.19 0.00143

NFKB1 SIGLEC10 -0.28 3.46e^-06^

NFKB1 SLAMF8 -0.34 5.5e^-09^

NFKB1 PTPRC -0.42 6.55e^-13^

NFKB1 TRAT1 -0.34 8.36e^-09^

NFKB1 WDR62 -0.38 1.26e^-10^

NFKB1 CCNA2 -0.47 1.83e^-16^

NFKB1 BIRC5 -0.37 4.48e^-10^

NFKB1 PLK1 -0.31 2.10e^-07^

NFKB1 C3 -0.36 8.62e^-10^

NFKB1 UCHL1 -0.21 0.00067

NFKB1 IFI6 -0.3 6.13e^-07^

NFKB1 CARD16 -0.47 2.21e^-16^

NFKB1 SAMHD1 -0.45 3.75e^-15^

NFKB1 IFI27 -0.4 4.17e^-12^

NFKB1 LILRA2 -0.41 3.22e^-12^

NFKB1 FCGR2A -0.49 6.91e^-18^

NFKB1 SIGLEC9 -0.48 7.31e^-17^

NFKB1 CD53 -0.45 6.62e^-15^

NFKB1 NCKAP1L -0.44 2.00e^-14^

NFKB1 SIGLEC7 -0.32 9.59e^-08^

NFKB1 SIGLEC8 -0.37 2.95e^-10^

NFKB1 RUNX3 -0.44 4.64e^-14^

NFKB1 CD37 -0.43 9.66e^-14^

NFKB1 SPI1 -0.48 3.78e^-17^

NFKB1 BTN3A1 -0.5 2.98e^-18^

NFKB1 IL2 -0.32 7.31e^-08^

NFKB1 ISG20 -0.42 4.85e^-13^

NFKB1 TNFRSF18 -0.32 7.16e^-08^

NFKB1 TREM1 -0.2 0.0012

NFKB1 APOBEC3G -0.13 0.02873

NFKB1 UBD -0.21 0.00044

NFKB1 CXCL11 -0.27 7.68e^-06^

NFKB1 CD27 -0.13 0.03501

NFKB1 LY9 -0.35 1.81e^-09^

NFKB1 SLAMF7 -0.32 8.65e^-08^

NFKB1 IRF4 -0.13 0.03508

NFKB1 SYP -0.22 0.000196

NFKB1 LGALS4 -0.15 0.011544

NFKB1 CTSG 0.15 0.012346

NFKB1 BST2 -0.36 1.04e^-09^

NFKB1 IRF6 -0.31 1.77e^-07^

NFKB1 TP73 -0.33 3.17e^-08^

NFKB1 FFAR2 -0.28 2.48e^-06^

NFKB1 NLRP7 -0.13 0.030165

NFKB1 ELANE 0.59 4.55e^-27^

NFKB1 LY86 0.57 4.84e^-25^

NFKB1 FLI1 0.64 1.93e^-32^

NFKB1 LEP 0.68 9.11e^-39^

NFKB1 ACAP1 0.5 1.23e^-18^

NFKB1 TNIP3 0.64 2.25e^-32^

NFKB1 PPARG 0.75 3.11e^-49^

NFKB1 TRIM36 0.56 1.01e^-23^

NFKB1 C5AR1 0.59 3.49e^-27^

NFKB1 LGALS9 0.28 3.21e^-06^

NFKB1 GLI1 0.27 5.26e^-06^

NFKB1 TNFRSF13B 0.62 1.75e^-30^

NFKB1 PRKCE 0.61 5.6e^-29^

NFKB1 DEFA6 0.53 4.89e^-21^

NFKB1 TET2 0.43 1.46e^-13^

NFKB1 F2RL2 0.29 1.11e^-06^

NFKB1 CD300E 0.39 3.57e^-11^

NFKB1 TRIM60 0.26 1.33e^-05^

NFKB1 PROCR 0.39 4.57e^-11^

NFKB1 NLRP2 0.39 1.96e^-11^

NFKB1 CAMK2A 0.29 9.17e^-07^

NFKB1 EFTUD2 0.26 1.81e^-05^

NFKB1 IL10 0.13 0.03261

DDX58 F2RL2 0.46 9.75e^-16^

DDX58 TET2 0.46 7.31e^-16^

DDX58 DEFA6 0.4 1.05e^-11^

DDX58 PRKCE 0.37 5.51e^-10^

DDX58 TNFRSF13B 0.54 2.38e^-22^

DDX58 GLI1 0.23 0.00011

DDX58 LGALS9 0.26 1.09e^-05^

DDX58 C5AR1 0.37 5.64e^-10^

DDX58 TRIM36 0.32 8.16e^-08^

DDX58 PPARG 0.39 5.17e^-11^

DDX58 TNIP3 0.44 1.84e^-14^

DDX58 ACAP1 0.42 8.85e^-13^

DDX58 LEP 0.47 1.62e^-16^

DDX58 FLI1 0.63 6.84e^-31^

DDX58 LY86 0.55 1.43e^-22^

DDX58 ELANE 0.27 7.02e^-06^

DDX58 PDCD1LG2 0.18 0.00246

DDX58 PRKDC -0.12 0.04371

DDX58 PQBP1 0.17 0.00631

DDX58 VENTX -0.3 5.6e^-07^

DDX58 LTB4R -0.37 2.49e^-10^

DDX58 YJEFN3 -0.27 7.19e^-06^

DDX58 LAT -0.28 2.06e^-06^

DDX58 F11 -0.16 0.00702

DDX58 USP2 -0.17 0.00441

DDX58 F2RL3 -0.28 3.87e^-06^

DDX58 CTNNAL1 -0.36 7.5e^-10^

DDX58 KITLG -0.3 6.19e^-07^

DDX58 CD1D -0.36 1.28e^-09^

DDX58 VEGFA -0.37 4.48e^-10^

DDX58 MAP3K14 -0.3 6.92e^-07^

DDX58 TRADD -0.39 2.12e^-11^

DDX58 OAS1 -0.23 0.00013

DDX58 ANXA4 -0.3 6.12e^-07^

DDX58 TLR3 -0.24 6.38e^-05^

DDX58 PELI2 -0.36 7.63e^-10^

DDX58 TRIM9 -0.31 1.59e^-07^

DDX58 SELE -0.23 0.00018

DDX58 C7 -0.24 6.09e^-05^

DDX58 CXCL12 -0.27 8.57e^-06^

DDX58 TLR6 -0.18 0.00226

DDX58 SIGLEC10 -0.15 0.01568

DDX58 SLAMF8 -0.2 0.00115

DDX58 PTPRC -0.19 0.00144

DDX58 TRAT1 -0.15 0.01226

DDX58 WDR62 -0.26 1.09e^-05^

DDX58 CCNA2 -0.33 4.14e^-08^

DDX58 BIRC5 -0.29 1.11e^-06^

DDX58 PLK1 -0.25 2.69e^-05^

DDX58 C3 -0.23 0.00016

DDX58 UCHL1 -0.16 0.00868

DDX58 IFI6 -0.17 0.00555

DDX58 CARD16 -0.31 1.67e^-07^

DDX58 SAMHD1 -0.26 1.41e^-05^

DDX58 IFI27 -0.24 6.43e^-05^

DDX58 LILRA2 -0.17 0.00413

DDX58 FCGR2A -0.31 2.92e^-07^

DDX58 SIGLEC9 -0.28 3.06e^-06^

DDX58 CD53 -0.22 0.00025

DDX58 NCKAP1L -0.23 0.00011

DDX58 SIGLEC7 -0.16 0.00984

DDX58 SIGLEC8 -0.18 0.00314

DDX58 RUNX3 -0.23 0.00012

DDX58 CD37 -0.25 2.5e^-05^

DDX58 SPI1 -0.3 7.23e^-07^

DDX58 BTN3A1 -0.25 2.59e^-05^

DDX58 IL2 -0.19 0.0021

DDX58 ISG20 -0.29 1.03e^-06^

DDX58 TNFRSF18 -0.25 2.9e^-05^

DDX58 TREM1 -0.15 0.0142

DDX58 CTLA4 0.14 0.01771

DDX58 LY9 -0.19 0.00217

DDX58 SLAMF7 -0.17 0.00422

DDX58 SYP -0.19 0.00129

DDX58 BST2 -0.25 3.1e^-05^

DDX58 IRF6 -0.26 1.11e^-05^

DDX58 TP73 -0.21 0.00045

DDX58 FFAR2 -0.18 0.00251

DDX58 CD300E 0.62 2.19e^-30^

DDX58 TRIM60 0.49 1.24e^-17^

DDX58 PROCR 0.29 9.16e^-07^

DDX58 NLRP2 0.27 7.01e^-06^

DDX58 CAMK2A 0.35 4.29e^-09^

DDX58 IL10 0.28 3.33e^-06^

CASP5 LGALS9 0.5 1.12e^-18^

CASP5 C5AR1 0.42 3.34e^-13^

CASP5 TRIM36 0.53 4.54e^-21^

CASP5 PPARG 0.34 1.1e^-08^

CASP5 TNIP3 0.39 4.98e^-11^

CASP5 ACAP1 0.42 6.39e^-13^

CASP5 LEP 0.55 9.1e^-23^

CASP5 FLI1 0.5 1.16e^-18^

CASP5 LY86 0.5 6.56e^-19^

CASP5 ELANE 0.34 5.58e^-09^

CASP5 PDCD1LG2 0.25 3.33e^-05^

CASP5 PRKDC -0.14 0.02526

CASP5 HIF1AN 0.13 0.02852

CASP5 VENTX -0.26 1.3e^-05^

CASP5 LTB4R -0.34 1.4e^-08^

CASP5 YJEFN3 -0.25 4.28e^-05^

CASP5 LAT -0.23 0.00011

CASP5 F11 -0.22 0.00035

CASP5 USP2 -0.22 0.00025

CASP5 F2RL3 -0.31 1.6e^-07^

CASP5 CTNNAL1 -0.32 1.16e^-07^

CASP5 KITLG -0.24 6.07e^-05^

CASP5 CD1D -0.37 2.55e^-10^

CASP5 VEGFA -0.41 1.52e^-12^

CASP5 MAP3K14 -0.37 4.09e^-10^

CASP5 TRADD -0.44 2.65e^-14^

CASP5 OAS1 -0.31 1.43e^-07^

CASP5 ANXA4 -0.41 2.07e^-12^

CASP5 TLR3 -0.38 1.18e^-10^

CASP5 PELI2 -0.39 1.92e^-11^

CASP5 TRIM9 -0.34 9.68e^-09^

CASP5 SELE -0.17 0.00597

CASP5 C7 -0.17 0.00396

CASP5 CXCL12 -0.29 1.34e^-06^

CASP5 FCGR1A 0.37 4.94e^-10^

CASP5 IFNG 0.14 0.02603

CASP5 WDR62 -0.19 0.00222

CASP5 CCNA2 -0.17 0.00493

CASP5 BIRC5 -0.13 0.0297

CASP5 C3 -0.13 0.02725

CASP5 IFI6 -0.19 0.00129

CASP5 CARD16 -0.28 2.63e^-06^

CASP5 SAMHD1 -0.18 0.00255

CASP5 IFI27 -0.27 5e^-06^

CASP5 LILRA2 -0.22 0.00026

CASP5 FCGR2A -0.19 0.0018

CASP5 SIGLEC9 -0.15 0.01297

CASP5 NCKAP1L -0.12 0.04253

CASP5 RUNX3 -0.24 8.16e^-05^

CASP5 CD37 -0.19 0.00177

CASP5 SPI1 -0.18 0.00233

CASP5 BTN3A1 -0.29 8.43e^-07^

CASP5 IL2 -0.18 0.00238

CASP5 ISG20 -0.25 4.47e^-05^

CASP5 TNFRSF18 -0.2 0.00112

CASP5 CTLA4 0.14 0.02171

CASP5 CXCL9 0.19 0.00142

CASP5 LY9 -0.14 0.02036

CASP5 IRF4 0.29 8.69e^-07^

CASP5 NOX4 0.14 0.01936

CASP5 LGALS4 -0.14 0.02467

CASP5 BST2 -0.24 4.59e^-05^

CASP5 IRF6 -0.26 1.19e^-05^

CASP5 TP73 -0.14 0.02088

CASP5 FFAR2 -0.19 0.00134

CASP5 GLI1 0.35 2.17e^-09^

CASP5 TNFRSF13B 0.44 3.31e^-14^

CASP5 PRKCE 0.27 7.64e^-06^

CASP5 DEFA6 0.35 3.59e^-09^

CASP5 TET2 0.36 1.67e^-09^

CASP5 F2RL2 0.27 4.69e^-06^

CASP5 CD300E 0.34 1.03e^-08^

CASP5 TRIM60 0.51 1.18e^-19^

CASP5 PROCR 0.41 1.36e^-12^

CASP5 NLRP2 0.21 0.00037

CASP5 CAMK2A 0.28 4.13e^-06^

VDR IRF6 0.61 2.04e^-29^

VDR TP73 0.31 2.82e^-07^

VDR FFAR2 0.26 1.03e^-05^

VDR NLRP7 0.14 0.0241

VDR BST2 0.32 6.77e^-08^

VDR SYP 0.17 0.00416

VDR NOX4 -0.17 0.00472

VDR SLAMF7 0.36 1.39e^-09^

VDR LY9 0.34 1.53e^-08^

VDR CD27 0.21 0.00063

VDR CXCL9 0.15 0.01571

VDR CXCL11 0.24 6.95e^-05^

VDR UBD 0.21 0.00051

VDR TREM1 0.19 0.0017

VDR MMP12 0.17 0.0068

VDR TNFRSF18 0.27 8.89e^-06^

VDR ISG20 0.38 1.52e^-10^

VDR IL2 0.3 3.31e^-07^

VDR BTN3A1 0.43 2.3e^-13^

VDR SPI1 0.47 2.63e^-16^

VDR CD37 0.43 1.21e^-13^

VDR RUNX3 0.43 2.31e^-13^

VDR SIGLEC8 0.36 7.92e^-10^

VDR SIGLEC7 0.36 6.21e^-10^

VDR NCKAP1L 0.43 1.15e^-13^

VDR CD53 0.44 3.95e^-14^

VDR SIGLEC9 0.48 8.87e^-17^

VDR FCGR2A 0.47 2.18e^-16^

VDR LILRA2 0.35 2.22e^-09^

VDR IFI27 0.37 4e^-10^

VDR SAMHD1 0.4 1.27e^-11^

VDR CARD16 0.38 1.15e^-10^

VDR IFI6 0.28 3.85e^-06^

VDR UCHL1 0.24 8.09e^-05^

VDR C3 0.3 5.85e^-07^

VDR PLK1 0.28 2.06e^-06^

VDR BIRC5 0.38 1.52e^-10^

VDR CCNA2 0.46 1.72e^-15^

VDR WDR62 0.38 1.65e^-10^

VDR TRAT1 0.36 1.13e^-09^

VDR PTPRC 0.41 2.32e^-12^

VDR SLAMF8 0.4 1.46e^-11^

VDR SIGLEC10 0.33 4.2e^-08^

VDR IFNG 0.26 2.13e^-05^

VDR TLR6 0.39 3.62e^-11^

VDR FCGR1A 0.16 0.00723

VDR CXCL12 0.42 7.86e^-13^

VDR C7 0.21 0.0005

VDR SELE 0.21 0.00047

VDR TRIM9 0.33 2.33e^-08^

VDR PELI2 0.48 2.68e^-17^

VDR TLR3 0.4 6.75e^-12^

VDR ANXA4 0.35 3.87e^-09^

VDR OAS1 0.45 6.54e^-15^

VDR TRADD 0.5 2.77e^-18^

VDR MAP3K14 0.44 4.11e^-14^

VDR VEGFA 0.3 4.09e^-07^

VDR CD1D 0.37 2.42e^-10^

VDR KITLG 0.53 5.89e^-21^

VDR CTNNAL1 0.54 4.06e^-22^

VDR F2RL3 0.23 0.00016

VDR USP2 0.24 8.75e^-05^

VDR F11 0.22 0.00035

VDR LAT 0.31 2.04e^-07^

VDR YJEFN3 0.27 4.73e^-06^

VDR LTB4R 0.36 8.09e^-10^

VDR VENTX 0.35 2.67e^-09^

VDR NLRP11 -0.13 0.028

VDR CALCA 0.32 4.71e^-08^

VDR PQBP1 -0.27 4.67e^-06^

VDR HIF1AN -0.35 2.07e^-09^

VDR PDCD1LG2 -0.29 1.04e^-06^

VDR ELANE -0.3 3.85e^-07^

VDR LY86 -0.39 4.69e^-11^

VDR FLI1 -0.46 1.2e^-15^

VDR LEP -0.48 3.5e^-17^

VDR ACAP1 -0.33 3.16e^-08^

VDR TNIP3 -0.36 1.19e^-09^

VDR PPARG -0.41 1.29e^-12^

VDR TRIM36 -0.42 3.67e^-13^

VDR C5AR1 -0.38 1.6e^-10^

VDR LGALS9 -0.23 9.7e^-05^

VDR GLI1 -0.35 4.82e^-09^

VDR TNFRSF13B -0.47 3.24e^-16^

VDR PRKCE -0.41 1.25e^-12^

VDR DEFA6 -0.35 4.41e^-09^

VDR TET2 -0.35 4.67e^-09^

VDR F2RL2 -0.27 6.1e^-06^

VDR CD300E -0.28 1.96e^-06^

VDR TRIM60 -0.31 3.04e^-07^

VDR PROCR -0.31 1.86e^-07^

VDR NLRP2 -0.25 3.5e^-05^

VDR CAMK2A -0.22 0.00028

VDR EFTUD2 -0.18 0.00377

DFNB59 EFTUD2 0.28 2.29e^-06^

DFNB59 CAMK2A 0.57 1.56e^-24^

DFNB59 NLRP2 0.16 0.01

DFNB59 PROCR 0.22 0.00025

DFNB59 TRIM60 0.4 1.06e^-11^

DFNB59 CD300E 0.38 9.6e^-11^

DFNB59 F2RL2 0.28 2.06e^-06^

DFNB59 TET2 0.33 4.08e^-08^

DFNB59 DEFA6 0.42 8.47e^-13^

DFNB59 PRKCE 0.19 0.0018

DFNB59 TNFRSF13B 0.35 4.56e^-09^

DFNB59 GLI1 0.25 2.18e^-05^

DFNB59 LGALS9 0.51 3.76e^-19^

DFNB59 C5AR1 0.31 2.86e^-07^

DFNB59 TRIM36 0.21 0.00065

DFNB59 PPARG 0.47 3.55e^-16^

DFNB59 TNIP3 0.66 9.09e^-35^

DFNB59 ACAP1 0.7 1.14e^-40^

DFNB59 LEP 0.44 2.07e^-14^

DFNB59 FLI1 0.68 1.12e^-38^

DFNB59 LY86 0.72 3.07e^-44^

DFNB59 ELANE 0.29 7.82e^-07^

DFNB59 PDCD1LG2 0.26 1.31e^-05^

DFNB59 PRKDC -0.17 0.00546

DFNB59 PQBP1 0.14 0.0194

DFNB59 VENTX -0.13 0.03108

DFNB59 LTB4R -0.15 0.01557

DFNB59 F2RL3 -0.18 0.0026

DFNB59 CTNNAL1 -0.25 4.53e^-05^

DFNB59 KITLG -0.22 0.00019

DFNB59 CD1D -0.28 1.97e^-06^

DFNB59 VEGFA -0.21 0.0005

DFNB59 MAP3K14 -0.22 0.00021

DFNB59 TRADD -0.26 1.4e^-05^

DFNB59 OAS1 -0.25 2.48e^-05^

DFNB59 ANXA4 -0.28 2e^-06^

DFNB59 TLR3 -0.31 1.97e^-07^

DFNB59 PELI2 -0.24 9.28e^-05^

DFNB59 TRIM9 -0.24 5.24e^-05^

DFNB59 SELE -0.17 0.00465

DFNB59 C7 -0.13 0.034

DFNB59 CXCL12 -0.23 9.44e^-05^

DFNB59 FCGR1A 0.17 0.00464

DFNB59 TLR6 -0.17 0.00551

DFNB59 SIGLEC10 -0.17 0.00514

DFNB59 SLAMF8 -0.15 0.01354

DFNB59 PTPRC -0.25 4.42e^-05^

DFNB59 TRAT1 -0.19 0.00127

DFNB59 CCNA2 -0.23 0.00011

DFNB59 BIRC5 -0.15 0.01073

DFNB59 PLK1 -0.14 0.02164

DFNB59 C3 -0.21 0.00041

DFNB59 CARD16 -0.18 0.00227

DFNB59 SAMHD1 -0.24 8.47e^-05^

DFNB59 IFI27 -0.16 0.00779

DFNB59 LILRA2 -0.14 0.02164

DFNB59 FCGR2A -0.28 1.89e^-06^

DFNB59 SIGLEC9 -0.27 5.49e^-06^

DFNB59 CD53 -0.26 1.98e^-05^

DFNB59 NCKAP1L -0.26 1.75e^-05^

DFNB59 SIGLEC7 -0.18 0.00306

DFNB59 SIGLEC8 -0.24 9.3e^-05^

DFNB59 RUNX3 -0.23 0.00017

DFNB59 CD37 -0.14 0.0249

DFNB59 SPI1 -0.25 3.21e^-05^

DFNB59 BTN3A1 -0.27 5.1e^-06^

DFNB59 IL2 -0.14 0.01884

DFNB59 ISG20 -0.15 0.01541

DFNB59 CTLA4 0.35 1.88e^-09^

DFNB59 UBD -0.18 0.00232

DFNB59 CXCL11 -0.16 0.00985

DFNB59 SLAMF7 -0.18 0.00381

DFNB59 CTSG 0.39 1.83e^-11^

DFNB59 BST2 -0.13 0.02852

DFNB59 TP73 -0.12 0.04097

RIPK3 IL10 0.29 1.43e^-06^

RIPK3 EFTUD2 0.15 0.01411

RIPK3 CAMK2A 0.27 4.76e^-06^

RIPK3 NLRP2 0.17 0.00408

RIPK3 PROCR 0.22 0.00022

RIPK3 TRIM60 0.17 0.00473

RIPK3 CD300E 0.38 5.87e^-11^

RIPK3 F2RL2 0.28 2.08e^-06^

RIPK3 TET2 0.46 2.23e^-15^

RIPK3 DEFA6 0.42 2.97e^-13^

RIPK3 PRKCE 0.66 2.45e^-35^

RIPK3 TNFRSF13B 0.45 6.65e^-15^

RIPK3 GLI1 0.21 0.00045

RIPK3 LGALS9 0.26 1.46e^-05^

RIPK3 C5AR1 0.45 3.4e^-15^

RIPK3 TRIM36 0.29 1.33e^-06^

RIPK3 PPARG 0.51 2.95e^-19^

RIPK3 TNIP3 0.54 1.55e^-21^

RIPK3 ACAP1 0.45 7.63e^-15^

RIPK3 LEP 0.5 1.36e^-18^

RIPK3 FLI1 0.56 6.99e^-24^

RIPK3 LY86 0.48 5.26e^-17^

RIPK3 ELANE 0.4 4.6e^-12^

RIPK3 PDCD1LG2 0.35 3.49e^-09^

RIPK3 HIF1AN 0.32 4.58e^-08^

RIPK3 PQBP1 0.45 6.47e^-15^

RIPK3 VENTX -0.28 2.15e^-06^

RIPK3 LTB4R -0.34 9.3e^-09^

RIPK3 YJEFN3 -0.21 0.00054

RIPK3 LAT -0.23 9.58e^-05^

RIPK3 F11 -0.19 0.00148

RIPK3 USP2 -0.19 0.0013

RIPK3 F2RL3 -0.27 6.58e^-06^

RIPK3 CTNNAL1 -0.35 3.84e^-09^

RIPK3 KITLG -0.3 5.67e^-07^

RIPK3 CD1D -0.39 3.6e^-11^

RIPK3 VEGFA -0.36 6.4e^-10^

RIPK3 MAP3K14 -0.37 1.96e^-10^

RIPK3 TRADD -0.43 2.27e^-13^

RIPK3 OAS1 -0.37 5.76e^-10^

RIPK3 ANXA4 -0.39 1.8e^-11^

RIPK3 TLR3 -0.39 3.74e^-11^

RIPK3 PELI2 -0.39 3.25e^-11^

RIPK3 TRIM9 -0.35 2.6e^-09^

RIPK3 SELE -0.21 0.00068

RIPK3 C7 -0.2 0.00071

RIPK3 CXCL12 -0.31 1.43e^-07^

RIPK3 TLR6 -0.3 5.32e^-07^

RIPK3 IFNG -0.13 0.03731

RIPK3 SIGLEC10 -0.19 0.00142

RIPK3 SLAMF8 -0.22 0.00031

RIPK3 PTPRC -0.29 8.23e^-07^

RIPK3 TRAT1 -0.23 0.00014

RIPK3 WDR62 -0.26 1.48e^-05^

RIPK3 CCNA2 -0.34 1.26e^-08^

RIPK3 BIRC5 -0.26 1.01e^-05^

RIPK3 PLK1 -0.22 0.00022

RIPK3 C3 -0.27 9.44e^-06^

RIPK3 UCHL1 -0.14 0.01738

RIPK3 IFI6 -0.2 0.00072

RIPK3 CARD16 -0.32 7.28e^-08^

RIPK3 SAMHD1 -0.32 8.57e^-08^

RIPK3 IFI27 -0.29 1.82e^-06^

RIPK3 LILRA2 -0.25 2.27e^-05^

RIPK3 FCGR2A -0.35 2.74e^-09^

RIPK3 SIGLEC9 -0.34 1.17e^-08^

RIPK3 CD53 -0.31 2.66e^-07^

RIPK3 NCKAP1L -0.31 2.3e^-07^

RIPK3 SIGLEC7 -0.23 0.00014

RIPK3 SIGLEC8 -0.26 1.47e^-05^

RIPK3 RUNX3 -0.3 3.44e^-07^

RIPK3 CD37 -0.29 1.15e^-06^

RIPK3 SPI1 -0.34 1.49e^-08^

RIPK3 BTN3A1 -0.36 1.53e^-09^

RIPK3 IL2 -0.22 0.00031

RIPK3 ISG20 -0.28 4.14e^-06^

RIPK3 TNFRSF18 -0.21 0.00062

RIPK3 TREM1 -0.14 0.01986

RIPK3 CTLA4 0.16 0.00675

RIPK3 APOBEC3G -0.13 0.03549

RIPK3 UBD -0.13 0.03506

RIPK3 CXCL11 -0.18 0.00265

RIPK3 LY9 -0.23 0.00014

RIPK3 SLAMF7 -0.21 0.00054

RIPK3 CTSG 0.18 0.00266

RIPK3 BST2 -0.25 2.58e^-05^

RIPK3 IRF6 -0.22 0.0002

RIPK3 TP73 -0.24 7.83e^-05^

RIPK3 FFAR2 -0.21 0.00067

BIRC3 CAMK2A -0.17 0.00474

BIRC3 NLRP2 -0.19 0.00194

BIRC3 PROCR -0.22 0.00021

BIRC3 TRIM60 -0.24 7.38e^-05^

BIRC3 CD300E -0.15 0.0166

BIRC3 F2RL2 -0.23 0.0001

BIRC3 TET2 -0.23 0.00013

BIRC3 DEFA6 -0.29 1.51e^-06^

BIRC3 PRKCE -0.31 3.01e^-07^

BIRC3 TNFRSF13B -0.37 1.89e^-10^

BIRC3 GLI1 -0.22 0.00026

BIRC3 C5AR1 -0.18 0.00334

BIRC3 TRIM36 -0.29 8.36e^-07^

BIRC3 PPARG -0.29 1.3e^-06^

BIRC3 TNIP3 -0.23 9.79e^-05^

BIRC3 ACAP1 -0.17 0.005

BIRC3 LEP -0.32 4.81e^-08^

BIRC3 FLI1 -0.33 1.93e^-08^

BIRC3 LY86 -0.24 8.88e^-05^

BIRC3 ELANE -0.2 0.00102

BIRC3 PDCD1LG2 -0.17 0.00512

BIRC3 PRKDC 0.19 0.0013

BIRC3 HIF1AN -0.21 0.00038

BIRC3 PQBP1 -0.24 5.67e^-05^

BIRC3 VENTX 0.35 5.11e^-09^

BIRC3 LTB4R 0.29 1.41e^-06^

BIRC3 LAT 0.24 4.85e^-05^

BIRC3 F2RL3 0.12 0.0418

BIRC3 CTNNAL1 0.25 3.9e^-05^

BIRC3 KITLG 0.18 0.00264

BIRC3 CD1D 0.33 2.56e^-08^

BIRC3 VEGFA 0.19 0.00146

BIRC3 MAP3K14 0.48 2.46e^-17^

BIRC3 TRADD 0.35 2.36e^-09^

BIRC3 OAS1 0.52 4.94e^-20^

BIRC3 ANXA4 0.34 1.2e^-08^

BIRC3 TLR3 0.47 3.05e^-16^

BIRC3 PELI2 0.24 6.84e^-05^

BIRC3 TRIM9 0.39 3.41e^-11^

BIRC3 SELE 0.15 0.01634

BIRC3 C7 0.13 0.03332

BIRC3 CXCL12 0.21 0.00048

BIRC3 FCGR1A 0.53 2.46e^-21^

BIRC3 TLR6 0.75 1.53e^-50^

BIRC3 IFNG 0.58 3.13e^-26^

BIRC3 SIGLEC10 0.64 9.96e^-33^

BIRC3 SLAMF8 0.66 8.95e^-35^

BIRC3 PTPRC 0.74 1.08e^-48^

BIRC3 TRAT1 0.66 4.88e^-35^

BIRC3 WDR62 0.34 6.64e^-09^

BIRC3 CCNA2 0.53 2.9e^-21^

BIRC3 BIRC5 0.4 5.33e^-12^

BIRC3 PLK1 0.36 6.39e^-10^

BIRC3 C3 0.52 2.93e^-20^

BIRC3 UCHL1 0.41 1.53e^-12^

BIRC3 IFI6 0.22 0.00032

BIRC3 CARD16 0.39 1.53e^-11^

BIRC3 SAMHD1 0.61 5.29e^-29^

BIRC3 IFI27 0.27 4.56e^-06^

BIRC3 LILRA2 0.38 6.49e^-11^

BIRC3 FCGR2A 0.61 2.37e^-29^

BIRC3 SIGLEC9 0.67 3.7e^-37^

BIRC3 CD53 0.69 2.27e^-40^

BIRC3 NCKAP1L 0.72 2.84e^-44^

BIRC3 SIGLEC7 0.59 3.34e^-27^

BIRC3 SIGLEC8 0.58 7.32e^-26^

BIRC3 RUNX3 0.54 3.51e^-22^

BIRC3 CD37 0.53 2.6e^-21^

BIRC3 SPI1 0.55 5.89e^-23^

BIRC3 BTN3A1 0.57 9.99e^-25^

BIRC3 IL2 0.42 9.63e^-13^

BIRC3 ISG20 0.48 4.67e^-17^

BIRC3 TNFRSF18 0.32 1.06e^-07^

BIRC3 MMP12 0.19 0.00193

BIRC3 TREM1 0.28 3.05e^-06^

BIRC3 CLEC4E 0.18 0.00271

BIRC3 CTLA4 0.23 9.82e^-05^

BIRC3 APOBEC3G 0.32 6.43e^-08^

BIRC3 UBD 0.53 1.27e^-20^

BIRC3 CXCL11 0.56 4.68e^-24^

BIRC3 CXCL9 0.55 5.47e^-23^

BIRC3 CD27 0.45 9.4e^-15^

BIRC3 LY9 0.54 5.05e^-22^

BIRC3 SLAMF7 0.64 7.49e^-33^

BIRC3 IRF4 0.13 0.02839

BIRC3 BST2 0.32 1.08e^-07^

BIRC3 TP73 0.47 5.81e^-16^

BIRC3 FFAR2 0.19 0.00139

BIRC3 NLRP7 0.15 0.01643

NLRP6 ACAP1 0.28 3.03e^-06^

NLRP6 SLAMF8 0.3 5.87e^-07^

NLRP6 IRF4 0.56 1.57e^-23^

NLRP6 TNK1 0.34 9.78e^-09^

NLRP6 NOX4 0.53 1.82e^-21^

NLRP6 PRTN3 0.7 1.94e^-48^

NLRP6 SYP 0.59 4.52e^-27^

NLRP6 KIR3DL2 0.97 2.11e^-168^

BHLHE41 IL10 -0.14 0.0252

BHLHE41 CAMK2A -0.2 0.00105

BHLHE41 PROCR -0.19 0.00176

BHLHE41 TRIM60 -0.27 4.48e^-06^

BHLHE41 CD300E -0.16 0.00758

BHLHE41 F2RL2 -0.28 2.66e^-06^

BHLHE41 TET2 -0.22 0.0002

BHLHE41 DEFA6 -0.24 9.14e^-05^

BHLHE41 PRKCE -0.19 0.00161

BHLHE41 TNFRSF13B -0.32 6.1e^-08^

BHLHE41 GLI1 -0.14 0.01954

BHLHE41 TRIM36 -0.14 0.02517

BHLHE41 TNIP3 -0.18 0.00363

BHLHE41 LEP -0.17 0.00639

BHLHE41 FLI1 -0.26 9.89e^-06^

BHLHE41 LY86 -0.19 0.00133

BHLHE41 PQBP1 -0.21 0.00048

BHLHE41 VENTX 0.21 0.00042

BHLHE41 LTB4R 0.18 0.00258

BHLHE41 F2RL3 0.19 0.00143

BHLHE41 CTNNAL1 0.14 0.02534

BHLHE41 CD1D 0.21 0.00045

BHLHE41 VEGFA 0.29 1.01e^-06^

BHLHE41 MAP3K14 0.37 2.13e^-10^

BHLHE41 TRADD 0.3 6.89e^-07^

BHLHE41 OAS1 0.3 6.15e^-07^

BHLHE41 ANXA4 0.36 1.66e^-09^

BHLHE41 TLR3 0.4 7.23e^-12^

BHLHE41 PELI2 0.23 0.00012

BHLHE41 TRIM9 0.41 1.14e^-12^

BHLHE41 SELE 0.12 0.04132

BHLHE41 C7 0.13 0.0301

BHLHE41 CXCL12 0.23 0.00013

BHLHE41 TLR6 0.21 0.00063

BHLHE41 SIGLEC10 0.14 0.02392

BHLHE41 SLAMF8 0.17 0.00435

BHLHE41 PTPRC 0.2 0.00107

BHLHE41 CCNA2 0.16 0.00668

BHLHE41 PLK1 0.13 0.03783

BHLHE41 C3 0.34 5.56e^-09^

BHLHE41 SAMHD1 0.2 0.00095

BHLHE41 LILRA2 0.22 0.00028

BHLHE41 FCGR2A 0.29 1.21e^-06^

BHLHE41 SIGLEC9 0.28 3.59e^-06^

BHLHE41 CD53 0.22 0.00027

BHLHE41 NCKAP1L 0.23 0.00012

BHLHE41 SIGLEC7 0.22 0.00021

BHLHE41 SIGLEC8 0.22 0.00029

BHLHE41 RUNX3 0.23 0.00016

BHLHE41 CD37 0.14 0.02622

BHLHE41 SPI1 0.19 0.0019

BHLHE41 BTN3A1 0.21 0.00042

BHLHE41 IL2 0.2 0.00094

BHLHE41 TREM1 0.17 0.00554

BHLHE41 C4BPA 0.13 0.03132

BHLHE41 LY9 0.13 0.033

BHLHE41 TNK1 0.18 0.00227

BHLHE41 NOX4 0.14 0.01874

BHLHE41 PRTN3 0.13 0.0355

BHLHE41 BST2 0.16 0.00969

BHLHE41 TP73 0.18 0.00228

SCGB3A2 CAMK2A -0.12 0.04481

SCGB3A2 NLRP2 -0.13 0.02795

SCGB3A2 PROCR -0.15 0.0148

SCGB3A2 TRIM60 -0.16 0.00966

SCGB3A2 CD300E -0.15 0.01527

SCGB3A2 F2RL2 -0.13 0.03224

SCGB3A2 TET2 -0.18 0.00259

SCGB3A2 DEFA6 -0.19 0.00177

SCGB3A2 PRKCE -0.22 0.00028

SCGB3A2 TNFRSF13B -0.25 2.96e^-05^

SCGB3A2 GLI1 -0.18 0.00335

SCGB3A2 C5AR1 -0.18 0.0033

SCGB3A2 TRIM36 -0.22 0.00031

SCGB3A2 PPARG -0.23 0.00018

SCGB3A2 TNIP3 -0.19 0.0018

SCGB3A2 ACAP1 -0.18 0.00332

SCGB3A2 LEP -0.25 2.53e^-05^

SCGB3A2 FLI1 -0.23 0.00011

SCGB3A2 LY86 -0.2 0.00116

SCGB3A2 ELANE -0.16 0.00901

SCGB3A2 PDCD1LG2 -0.16 0.00924

SCGB3A2 HIF1AN -0.19 0.00197

SCGB3A2 PQBP1 -0.15 0.0152

SCGB3A2 VENTX 0.26 9.84e^-06^

SCGB3A2 LTB4R 0.32 1.07e^-07^

SCGB3A2 LAT 0.18 0.00368

SCGB3A2 CTNNAL1 0.17 0.00578

SCGB3A2 CD1D 0.24 5.65e^-05^

SCGB3A2 VEGFA 0.2 0.00108

SCGB3A2 MAP3K14 0.19 0.00132

SCGB3A2 TRADD 0.22 0.00033

SCGB3A2 OAS1 0.17 0.0052

SCGB3A2 ANXA4 0.22 0.00021

SCGB3A2 TLR3 0.19 0.00129

SCGB3A2 PELI2 0.2 0.00097

SCGB3A2 TRIM9 0.33 2.5e^-08^

SCGB3A2 SELE 0.25 4.04e^-05^

SCGB3A2 C7 0.18 0.0024

SCGB3A2 CXCL12 0.29 1.54e^-06^

SCGB3A2 TLR6 0.28 1.88e^-06^

SCGB3A2 SIGLEC10 0.13 0.03441

SCGB3A2 SLAMF8 0.16 0.00803

SCGB3A2 PTPRC 0.2 0.00071

SCGB3A2 CCNA2 0.2 0.00074

SCGB3A2 BIRC5 0.14 0.01969

SCGB3A2 PLK1 0.13 0.02718

SCGB3A2 C3 0.44 2.2e^-14^

SCGB3A2 CARD16 0.17 0.00647

SCGB3A2 SAMHD1 0.24 7.12e^-05^

SCGB3A2 IFI27 0.18 0.00311

SCGB3A2 LILRA2 0.22 0.00036

SCGB3A2 FCGR2A 0.35 4.72e^-09^

SCGB3A2 SIGLEC9 0.29 7.8e^-07^

SCGB3A2 CD53 0.23 0.00015

SCGB3A2 NCKAP1L 0.25 3.15e^-05^

SCGB3A2 SIGLEC7 0.19 0.00128

SCGB3A2 SIGLEC8 0.21 0.00058

SCGB3A2 RUNX3 0.15 0.01558

SCGB3A2 CD37 0.19 0.00179

SCGB3A2 SPI1 0.24 7.36e^-05^

SCGB3A2 BTN3A1 0.19 0.00197

SCGB3A2 IL2 0.16 0.00791

SCGB3A2 ISG20 0.15 0.01112

SCGB3A2 LY9 0.15 0.01422

SCGB3A2 BST2 0.19 0.00155

SCGB3A2 TP73 0.12 0.04638

SCGB3A2 FFAR2 0.12 0.04673

GSDMB IL10 -0.16 0.0089

GSDMB EFTUD2 -0.18 0.00232

GSDMB NLRP2 -0.24 5.94e^-05^

GSDMB PROCR -0.29 1.78e^-06^

GSDMB TRIM60 -0.29 1.39e^-06^

GSDMB CD300E -0.26 1.57e^-05^

GSDMB F2RL2 -0.17 0.00536

GSDMB TET2 -0.36 1.71e^-09^

GSDMB DEFA6 -0.36 1.43e^-09^

GSDMB PRKCE -0.41 3.75e^-12^

GSDMB TNFRSF13B -0.46 2.44e^-15^

GSDMB GLI1 -0.32 9.24e^-08^

GSDMB LGALS9 -0.19 0.00207

GSDMB C5AR1 -0.39 4.8e^-11^

GSDMB TRIM36 -0.4 5.39e^-12^

GSDMB PPARG -0.41 1.65e^-12^

GSDMB TNIP3 -0.35 3.38e^-09^

GSDMB ACAP1 -0.28 4.14e^-06^

GSDMB LEP -0.47 5.06e^-16^

GSDMB FLI1 -0.46 1.17e^-15^

GSDMB LY86 -0.39 1.54e^-11^

GSDMB ELANE -0.3 5.42e^-07^

GSDMB PDCD1LG2 -0.29 1.13e^-06^

GSDMB HIF1AN -0.35 4.99e^-09^

GSDMB PQBP1 -0.25 2.75e^-05^

GSDMB NLRP11 -0.12 0.04612

GSDMB VENTX 0.53 9.18e^-21^

GSDMB LTB4R 0.76 1.06e^-52^

GSDMB YJEFN3 0.75 3.49e^-51^

GSDMB LAT 0.89 7.22e^-95^

GSDMB F11 0.23 0.0001

GSDMB USP2 0.16 0.00819

GSDMB F2RL3 0.22 0.00028

GSDMB CTNNAL1 0.26 1.52e^-05^

GSDMB KITLG 0.17 0.00438

GSDMB CD1D 0.47 2.4e^-16^

GSDMB VEGFA 0.5 6.4e^-19^

GSDMB MAP3K14 0.53 1.04e^-20^

GSDMB TRADD 0.55 9.79e^-23^

GSDMB OAS1 0.41 2.4e^-12^

GSDMB ANXA4 0.35 2.95e^-09^

GSDMB TLR3 0.24 7.85e^-05^

GSDMB PELI2 0.28 5.35e^-11^

GSDMB TRIM9 0.32 9.69e^-08^

GSDMB SELE 0.15 0.0156

GSDMB C7 0.15 0.01272

GSDMB CXCL12 0.22 0.00026

GSDMB TLR6 0.43 1.68e^-13^

GSDMB IFNG 0.25 3.82e^-05^

GSDMB SIGLEC10 0.23 9.86e^-05^

GSDMB SLAMF8 0.35 3.96e^-09^

GSDMB PTPRC 0.32 9.36e^-08^

GSDMB TRAT1 0.37 2.61e^-10^

GSDMB WDR62 0.67 2.08e^-36^

GSDMB CCNA2 0.55 1.38e^-22^

GSDMB BIRC5 0.5 3.01e^-18^

GSDMB PLK1 0.51 1.92e^-19^

GSDMB C3 0.21 0.00066

GSDMB UCHL1 0.21 0.00058

GSDMB IFI6 0.3 6.19e^-07^

GSDMB CARD16 0.5 1.45e^-18^

GSDMB SAMHD1 0.29 1.23e^-06^

GSDMB IFI27 0.41 2.92e^-12^

GSDMB LILRA2 0.19 0.00164

GSDMB FCGR2A 0.34 9.11e^-09^

GSDMB SIGLEC9 0.36 9.09e^-10^

GSDMB CD53 0.36 9.29e^-10^

GSDMB NCKAP1L 0.34 5.47e^-09^

GSDMB SIGLEC7 0.18 0.00288

GSDMB SIGLEC8 0.23 0.00015

GSDMB RUNX3 0.48 3.03e^-17^

GSDMB CD37 0.5 1.39e^-18^

GSDMB SPI1 0.51 1.38e^-19^

GSDMB BTN3A1 0.49 5.99e^-18^

GSDMB IL2 0.43 1.97e^-13^

GSDMB ISG20 0.66 1.23e^-34^

GSDMB TNFRSF18 0.57 5.97e^-25^

GSDMB CTLA4 0.15 0.01605

GSDMB APOBEC3G 0.22 0.0002

GSDMB UBD 0.17 0.00504

GSDMB CXCL11 0.28 4.27e^-06^

GSDMB CXCL9 0.18 0.00299

GSDMB CD27 0.33 2.59e^-08^

GSDMB LY9 0.54 7e^-22^

GSDMB SLAMF7 0.46 2.48e^-15^

GSDMB NOX4 -0.18 0.0033

GSDMB SYP 0.34 1.15e^-08^

GSDMB LGALS4 0.18 0.00288

GSDMB BST2 0.25 4.02e^-05^

GSDMB IRF6 0.22 0.00036

GSDMB TP73 0.45 5.21e^-15^

GSDMB FFAR2 0.41 2.68e^-12^

GSDMB NC 0.25 3.75e^-05^

GSDMB NLRP7 0.45 9.18e^-15^

ZBP1 HIF1AN 0.18 0.00336

ZBP1 CALCA 0.31 2.6e^-07^

ZBP1 NLRP11 0.9 2.46e^-98^

ZBP1 PSTPIP1 1 4.7e^-319^

ZBP1 APOBEC3G 0.37 1.81e^-10^

TLR2 EFTUD2 -0.16 0.00925

TLR2 CAMK2A -0.24 5.57e^-05^

TLR2 NLRP2 -0.25 3.25e^-05^

TLR2 PROCR -0.29 1.44e^-06^

TLR2 TRIM60 -0.29 1.25e^-06^

TLR2 CD300E -0.17 0.00645

TLR2 F2RL2 -0.28 3.49e^-06^

TLR2 TET2 -0.31 2.29e^-07^

TLR2 DEFA6 -0.38 1.55e^-10^

TLR2 PRKCE -0.43 6.37e^-14^

TLR2 TNFRSF13B -0.49 1.88e^-17^

TLR2 GLI1 -0.34 1.36e^-08^

TLR2 C5AR1 -0.31 2.95e^-07^

TLR2 TRIM36 -0.41 1.14e^-12^

TLR2 PPARG -0.4 1.35e^-11^

TLR2 TNIP3 -0.31 1.59e^-07^

TLR2 ACAP1 -0.2 0.00123

TLR2 LEP -0.45 8.66e^-15^

TLR2 FLI1 -0.41 1.98e^-12^

TLR2 LY86 -0.28 2.11e^-06^

TLR2 ELANE -0.28 4.05e^-06^

TLR2 PDCD1LG2 -0.27 6.05e^-06^

TLR2 PRKDC 0.18 0.00257

TLR2 HIF1AN -0.36 1.23e^-09^

TLR2 PQBP1 -0.31 1.19e^-07^

TLR2 NLRP11 -0.13 0.02676

TLR2 VENTX 0.53 4.26e^-21^

TLR2 LTB4R 0.41 1.3e^-12^

TLR2 YJEFN3 0.16 0.00676

TLR2 LAT 0.35 2.36e^-09^

TLR2 USP2 0.13 0.02846

TLR2 F2RL3 0.18 0.00383

TLR2 CTNNAL1 0.37 2.54e^-10^

TLR2 KITLG 0.35 5.36e^-09^

TLR2 CD1D 0.53 4.71e^-21^

TLR2 VEGFA 0.32 7.53e^-08^

TLR2 MAP3K14 0.47 1.79e^-16^

TLR2 TRADD 0.47 1.65e^-16^

TLR2 OAS1 0.55 1.31e^-22^

TLR2 ANXA4 0.42 6.68e^-13^

TLR2 TLR3 0.55 1.14e^-22^

TLR2 PELI2 0.41 2.14e^-12^

TLR2 TRIM9 0.42 7.02e^-13^

TLR2 SELE 0.31 1.54e^-07^

TLR2 C7 0.26 1e^-05^

TLR2 CXCL12 0.3 3.77e^-11^

TLR2 FCGR1A 0.56 3.44e^-24^

TLR2 TLR6 0.9 1.15e^-98^

TLR2 IFNG 0.6 1.54e^-27^

TLR2 SIGLEC10 0.72 7.01e^-44^

TLR2 SLAMF8 0.75 7.97e^-51^

TLR2 PTPRC 0.86 1.03e^-80^

TLR2 TRAT1 0.72 3.46e^-44^

TLR2 WDR62 0.38 7.58e^-11^

TLR2 CCNA2 0.64 5.99e^-33^

TLR2 BIRC5 0.43 7.84e^-14^

TLR2 PLK1 0.39 3.25e^-11^

TLR2 C3 0.6 2.23e^-28^

TLR2 UCHL1 0.38 7.84e^-11^

TLR2 IFI6 0.24 5.19e^-05^

TLR2 CARD16 0.47 2.96e^-16^

TLR2 SAMHD1 0.73 1.37e^-45^

TLR2 IFI27 0.36 1.2e^-09^

TLR2 LILRA2 0.62 1.92e^-30^

TLR2 FCGR2A 0.81 1.33e^-64^

TLR2 SIGLEC9 0.84 5.34e^-72^

TLR2 CD53 0.83 3.26e^-71^

TLR2 NCKAP1L 0.87 1.19e^-84^

TLR2 SIGLEC7 0.74 1.67e^-47^

TLR2 SIGLEC8 0.75 3.26e^-50^

TLR2 RUNX3 0.63 4.51e^-31^

TLR2 CD37 0.7 4.5e^-41^

TLR2 SPI1 0.73 3.98e^-47^

TLR2 BTN3A1 0.68 1.22e^-38^

TLR2 IL2 0.45 3.33e^-15^

TLR2 ISG20 0.46 1.67e^-15^

TLR2 TNFRSF18 0.33 1.58e^-08^

TLR2 MMP12 0.16 0.00993

TLR2 TREM1 0.32 8.2e^-08^

TLR2 CLEC4E 0.23 0.00012

TLR2 CTLA4 0.25 3.06e^-05^

TLR2 APOBEC3G 0.32 1.14e^-07^

TLR2 UBD 0.52 2.07e^-20^

TLR2 CXCL11 0.55 3.75e^-23^

TLR2 CXCL9 0.55 1e^-22^

TLR2 CD27 0.49 5.98e^-18^

TLR2 LY9 0.62 5.7e^-30^

TLR2 SLAMF7 0.67 1.26e^-36^

TLR2 SYP 0.14 0.01738

TLR2 BST2 0.32 9.41e^-08^

TLR2 IRF6 0.18 0.00379

TLR2 TP73 0.41 3.75e^-12^

TLR2 FFAR2 0.21 0.00062

AIM2 PROCR -0.14 0.02541

AIM2 F2RL2 -0.13 0.02679

AIM2 TET2 -0.16 0.00808

AIM2 PRKCE -0.16 0.00706

AIM2 TNFRSF13B -0.17 0.00444

AIM2 TRIM36 -0.18 0.00324

AIM2 PPARG -0.12 0.04215

AIM2 LEP -0.19 0.00206

AIM2 FLI1 -0.13 0.03274

AIM2 PRKDC 0.14 0.0233

AIM2 HIF1AN -0.14 0.01729

AIM2 VENTX 0.15 0.0145

AIM2 LTB4R 0.18 0.00319

AIM2 LAT 0.2 0.00073

AIM2 CTNNAL1 0.22 0.00025

AIM2 KITLG 0.25 2.71e^-05^

AIM2 CD1D 0.21 0.00038

AIM2 MAP3K14 0.22 0.00029

AIM2 TRADD 0.16 0.0099

AIM2 OAS1 0.28 3.69e^-06^

AIM2 TLR3 0.14 0.02289

AIM2 CXCL12 0.14 0.02279

AIM2 FCGR1A 0.72 9.1e^-44^

AIM2 TLR6 0.66 5.78e^-35^

AIM2 IFNG 0.85 3.42e^-76^

AIM2 SIGLEC10 0.82 5.05e^-67^

AIM2 SLAMF8 0.79 9.93e^-59^

AIM2 PTPRC 0.69 1.47e^-39^

AIM2 TRAT1 0.75 1.15e^-49^

AIM2 WDR62 0.38 9.09e^-11^

AIM2 CCNA2 0.58 3.05e^-26^

AIM2 BIRC5 0.45 4.2e^-15^

AIM2 PLK1 0.39 3.92e^-11^

AIM2 C3 0.36 1.03e^-09^

AIM2 UCHL1 0.41 2.31e^-12^

AIM2 IFI6 0.13 0.02986

AIM2 CARD16 0.32 5.62e^-08^

AIM2 SAMHD1 0.53 4.05e^-21^

AIM2 IFI27 0.2 0.0012

AIM2 LILRA2 0.23 0.00014

AIM2 FCGR2A 0.51 2.47e^-19^

AIM2 SIGLEC9 0.6 8.06e^-28^

AIM2 CD53 0.69 1.96e^-39^

AIM2 NCKAP1L 0.68 1.81e^-37^

AIM2 SIGLEC7 0.6 2.26e^-27^

AIM2 SIGLEC8 0.56 3.42e^-24^

AIM2 RUNX3 0.43 1.14e^-13^

AIM2 CD37 0.55 5.41e^-23^

AIM2 SPI1 0.57 4.53e^-25^

AIM2 BTN3A1 0.41 1.32e^-12^

AIM2 IL2 0.24 8.54e^-05^

AIM2 ISG20 0.39 2.67e^-11^

AIM2 TNFRSF18 0.25 3.14e^-05^

AIM2 MMP12 0.24 7.49e^-05^

AIM2 TREM1 0.16 0.00776

AIM2 CLEC4E 0.31 1.69e^-07^

AIM2 CTLA4 0.35 3.78e^-09^

AIM2 APOBEC3G 0.39 3.38e^-11^

AIM2 UBD 0.44 5.3e^-14^

AIM2 CXCL11 0.57 9.67e^-25^

AIM2 CXCL9 0.76 1.34e^-52^

AIM2 CD27 0.6 3.12e^-28^

AIM2 LY9 0.56 9.05e^-24^

AIM2 SLAMF7 0.77 1.95e^-53^

AIM2 IRF4 0.13 0.0394

AIM2 BST2 0.17 0.00401

AIM2 TP73 0.36 7.47e^-10^

AIM2 NLRP7 0.14 0.01881

IL1B EFTUD2 0.2 0.00113

IL1B CAMK2A 0.31 1.99e^-07^

IL1B NLRP2 0.18 0.00251

IL1B PROCR 0.14 0.0194

IL1B TRIM60 0.14 0.0235

IL1B CD300E 0.34 8.76e^-09^

IL1B F2RL2 0.15 0.01217

IL1B TET2 0.22 0.00027

IL1B DEFA6 0.23 0.00015

IL1B PRKCE 0.25 2.56e^-05^

IL1B TNFRSF13B 0.3 5.11e^-07^

IL1B GLI1 0.28 1.88e^-06^

IL1B LGALS9 0.46 7.16e^-16^

IL1B C5AR1 0.48 7.19e^-17^

IL1B TRIM36 0.31 1.78e^-07^

IL1B PPARG 0.55 7.91e^-23^

IL1B TNIP3 0.47 1.33e^-16^

IL1B ACAP1 0.68 2.27e^-38^

IL1B LEP 0.51 3.6e^-19^

IL1B FLI1 0.47 2.5e^-16^

IL1B LY86 0.6 1.99e^-28^

IL1B ELANE 0.53 1.83e^-21^

IL1B PDCD1LG2 0.4 6.3e^-12^

IL1B PRKDC -0.17 0.00599

IL1B HIF1AN 0.17 0.0048

IL1B VENTX -0.21 0.00056

IL1B LTB4R -0.28 2.42e^-06^

IL1B YJEFN3 -0.17 0.00386

IL1B LAT -0.21 0.00064

IL1B F11 -0.14 0.01851

IL1B USP2 -0.17 0.00578

IL1B F2RL3 -0.24 9.1e^-05^

IL1B CTNNAL1 -0.27 9.01e^-06^

IL1B KITLG -0.24 5.47e^-05^

IL1B CD1D -0.29 9.13e^-07^

IL1B VEGFA -0.3 5.67e^-07^

IL1B MAP3K14 -0.28 4.14e^-06^

IL1B TRADD -0.32 6.3e^-08^

IL1B OAS1 -0.23 0.00015

IL1B ANXA4 -0.28 3.66e^-06^

IL1B TLR3 -0.25 2.79e^-05^

IL1B PELI2 -0.28 2.43e^-06^

IL1B TRIM9 -0.25 3e^-05^

IL1B C7 -0.16 0.00882

IL1B CXCL12 -0.23 0.00015

IL1B FCGR1A 0.15 0.01354

IL1B TLR6 -0.17 0.0053

IL1B SIGLEC10 -0.12 0.0399

IL1B PTPRC -0.19 0.00137

IL1B TRAT1 -0.17 0.0054

IL1B WDR62 -0.21 0.00039

IL1B CCNA2 -0.26 2.04e^-05^

IL1B BIRC5 -0.2 0.0007

IL1B PLK1 -0.17 0.00429

IL1B C3 -0.15 0.0129

IL1B CARD16 -0.25 3.08e^-05^

IL1B SAMHD1 -0.21 0.00042

IL1B IFI27 -0.22 0.00033

IL1B FCGR2A -0.22 0.00036

IL1B SIGLEC9 -0.21 0.00045

IL1B CD53 -0.21 0.00061

IL1B NCKAP1L -0.2 0.00085

IL1B SIGLEC8 -0.17 0.00623

IL1B RUNX3 -0.22 0.0003

IL1B CD37 -0.18 0.00303

IL1B SPI1 -0.23 0.00015

IL1B BTN3A1 -0.26 2.07e^-05^

IL1B IL2 -0.12 0.04196

IL1B ISG20 -0.23 0.00016

IL1B TNFRSF18 -0.17 0.00508

IL1B CTLA4 0.13 0.02821

IL1B CXCL11 -0.14 0.02174

IL1B LY9 -0.14 0.01811

IL1B SLAMF7 -0.16 0.00917

IL1B NOX4 0.21 0.00062

IL1B PRTN3 0.17 0.00536

IL1B KIR3DL2 0.2 0.00097

IL1B NLRP6 0.2 0.00124

IL1B CTSG 0.24 7.26e^-05^

IL1B BST2 -0.18 0.00369

IL1B IRF6 -0.2 0.00106

IL1B TP73 -0.18 0.00295

GSDMC CALCA 0.17 0.00612

GSDMC YJEFN3 0.23 0.00011

GSDMC LAT 0.17 0.0045

GSDMC CTNNAL1 0.38 1.31e^-10^

GSDMC KITLG 0.44 3.38e^-14^

GSDMC TLR3 -0.12 0.0426

GSDMC PELI2 0.2 0.0012

GSDMC WDR62 0.21 0.00039

GSDMC CCNA2 0.16 0.00889

GSDMC BIRC5 0.16 0.00676

GSDMC PLK1 0.12 0.04641

GSDMC UBD -0.13 0.03673

GSDMC SYP 0.14 0.02132

GSDMC IRF6 0.7 1.12e^-41^

GSDMC DCD 0.15 0.0153

GSDMC NLRP7 0.13 0.03914

BDNF IL10 -0.16 0.00775

BDNF EFTUD2 -0.19 0.00181

BDNF CAMK2A -0.26 1.25e^-05^

BDNF NLRP2 -0.25 2.74e^-05^

BDNF PROCR -0.3 7.06e^-07^

BDNF TRIM60 -0.3 6.15e^-07^

BDNF CD300E -0.26 2.03e^-05^

BDNF F2RL2 -0.19 0.00169

BDNF TET2 -0.36 1.33e^-09^

BDNF DEFA6 -0.37 2.24e^-10^

BDNF PRKCE -0.42 6.53e^-13^

BDNF TNFRSF13B -0.47 1.7e^-16^

BDNF GLI1 -0.34 6.37e^-09^

BDNF LGALS9 -0.28 3.52e^-06^

BDNF C5AR1 -0.43 9.3e^-14^

BDNF TRIM36 -0.42 1.03e^-12^

BDNF PPARG -0.43 1.53e^-13^

BDNF TNIP3 -0.39 3.65e^-11^

BDNF ACAP1 -0.35 2.38e^-09^

BDNF LEP -0.49 1.83e^-17^

BDNF FLI1 -0.46 2.52e^-15^

BDNF LY86 -0.42 5.64e^-13^

BDNF ELANE -0.31 1.3e^-07^

BDNF PDCD1LG2 -0.31 2.47e^-07^

BDNF HIF1AN -0.36 1.49e^-09^

BDNF PQBP1 -0.27 5.16e^-06^

BDNF NLRP11 -0.12 0.04926

BDNF VENTX 0.4 5.25e^-12^

BDNF LTB4R 0.41 1.68e^-12^

BDNF YJEFN3 0.28 1.87e^-06^

BDNF LAT 0.28 2.59e^-06^

BDNF F11 0.29 1.48e^-06^

BDNF USP2 0.18 0.00377

BDNF F2RL3 0.37 3.19e^-10^

BDNF CTNNAL1 0.38 1.32e^-10^

BDNF KITLG 0.36 9.78e^-10^

BDNF CD1D 0.55 1.42e^-22^

BDNF VEGFA 0.54 2.88e^-22^

BDNF MAP3K14 0.47 5.91e^-16^

BDNF TRADD 0.53 3.98e^-21^

BDNF OAS1 0.42 5.08e^-13^

BDNF ANXA4 0.47 5.18e^-16^

BDNF TLR3 0.5 2.8e^-18^

BDNF PELI2 0.47 2.64e^-16^

BDNF TRIM9 0.41 1.56e^-12^

BDNF SELE 0.2 0.00086

BDNF C7 0.13 0.03518

BDNF CXCL12 0.4 5.03e^-12^

BDNF TLR6 0.28 2.66e^-06^

BDNF SIGLEC10 0.16 0.0069

BDNF SLAMF8 0.14 0.02059

BDNF PTPRC 0.3 4.54e^-07^

BDNF TRAT1 0.25 4.38e^-05^

BDNF WDR62 0.3 4.03e^-07^

BDNF CCNA2 0.35 5.08e^-09^

BDNF BIRC5 0.26 1.38e^-05^

BDNF PLK1 0.18 0.00229

BDNF C3 0.16 0.0089

BDNF IFI6 0.23 0.00015

BDNF CARD16 0.43 7.76e-^14^

BDNF SAMHD1 0.33 1.75e^-08^

BDNF IFI27 0.23 0.0001

BDNF LILRA2 0.34 6.17e^-09^

BDNF FCGR2A 0.36 1.4e^-09^

BDNF SIGLEC9 0.3 3.29e^-07^

BDNF CD53 0.33 3.24e^-08^

BDNF NCKAP1L 0.3 6.01e^-07^

BDNF SIGLEC7 0.23 0.00018

BDNF SIGLEC8 0.23 0.00018

BDNF RUNX3 0.26 1.13e^-05^

BDNF CD37 0.32 6.98e^-08^

BDNF SPI1 0.32 6.21e^-08^

BDNF BTN3A1 0.48 3.75e^-17^

BDNF IL2 0.2 0.00092

BDNF ISG20 0.23 0.00013

BDNF TNFRSF18 0.24 5.73e^-05^

BDNF ARG1 0.16 0.00871

BDNF UBD 0.24 8.64e^-05^

BDNF CXCL11 0.27 5.45e^-06^

BDNF LY9 0.21 0.00039

BDNF SLAMF7 0.16 0.00759

BDNF NOX4 -0.18 0.00357

BDNF SYP 0.18 0.003

BDNF BST2 0.24 5.62e^-05^

BDNF IRF6 0.21 0.00042

BDNF TP73 0.22 0.0003

BDNF FFAR2 0.25 2.53e^-05^

P2RX7 EFTUD2 -0.25 4.32e^-05^

P2RX7 CAMK2A -0.32 5.45e^-08^

P2RX7 NLRP2 -0.34 7.5e^-09^

P2RX7 PROCR -0.4 4.68e^-12^

P2RX7 TRIM60 -0.4 4.12e^-12^

P2RX7 CD300E -0.36 1.26e^-09^

P2RX7 F2RL2 -0.38 1.04e^-10^

P2RX7 TET2 -0.48 3.28e^-17^

P2RX7 DEFA6 -0.51 3.78e^-19^

P2RX7 PRKCE -0.57 9.5e^-25^

P2RX7 TNFRSF13B -0.64 4.77e^-33^

P2RX7 GLI1 -0.46 1.52e^-15^

P2RX7 LGALS9 -0.28 2.32e^-06^

P2RX7 C5AR1 -0.49 4.09e^-18^

P2RX7 TRIM36 -0.56 3.06e^-24^

P2RX7 PPARG -0.59 1.87e^-26^

P2RX7 TNIP3 -0.51 5.35e^-19^

P2RX7 ACAP1 -0.46 1.33e^-15^

P2RX7 LEP -0.66 2.07e^-35^

P2RX7 FLI1 -0.62 1.53e^-30^

P2RX7 LY86 -0.53 1.26e^-20^

P2RX7 ELANE -0.43 2e^-13^

P2RX7 PDCD1LG2 -0.41 1.16e^-12^

P2RX7 PRKDC 0.14 0.018

P2RX7 HIF1AN -0.48 1.02e^-16^

P2RX7 PQBP1 -0.38 9.38e^-11^

P2RX7 NLRP11 -0.15 0.01187

P2RX7 VENTX 0.54 3.73e^-22^

P2RX7 LTB4R 0.61 3.16e^-29^

P2RX7 YJEFN3 0.3 6.07e^-07^

P2RX7 LAT 0.49 7.78e^-18^

P2RX7 F11 0.26 1.89e^-05^

P2RX7 USP2 0.22 0.00023

P2RX7 F2RL3 0.33 4.02e^-08^

P2RX7 CTNNAL1 0.45 8.33e^-15^

P2RX7 KITLG 0.4 1.32e^-11^

P2RX7 CD1D 0.67 3.81e^-36^

P2RX7 VEGFA 0.58 8.51e^-26^

P2RX7 MAP3K14 0.65 4.49e^-34^

P2RX7 TRADD 0.68 1.38e^-37^

P2RX7 OAS1 0.7 4.9e^-41^

P2RX7 ANXA4 0.61 6.19e^-29^

P2RX7 TLR3 0.73 5.86e^-46^

P2RX7 PELI2 0.59 1.23e^-26^

P2RX7 TRIM9 0.6 5.16e^-28^

P2RX7 SELE 0.37 2.69e^-10^

P2RX7 C7 0.28 4.01e^-06^

P2RX7 CXCL12 0.5 1.14e^-18^

P2RX7 FCGR1A 0.34 7.01e^-09^

P2RX7 TLR6 0.77 1.63e^-54^

P2RX7 IFNG 0.49 8.78e^-18^

P2RX7 SIGLEC10 0.58 2.95e^-25^

P2RX7 SLAMF8 0.6 2.92e^-28^

P2RX7 PTPRC 0.78 7.69e^-56^

P2RX7 TRAT1 0.64 1.77e^-32^

P2RX7 WDR62 0.48 2.91e^-17^

P2RX7 CCNA2 0.71 6.41e^-43^

P2RX7 BIRC5 0.49 1.18e^-17^

P2RX7 PLK1 0.41 3.01e^-12^

P2RX7 C3 0.56 1.92e^-23^

P2RX7 UCHL1 0.24 9.06e^-05^

P2RX7 IFI6 0.3 3.2e^-07^

P2RX7 CARD16 0.59 3.94e^-27^

P2RX7 SAMHD1 0.73 1.39e^-45^

P2RX7 IFI27 0.44 2.66e^-14^

P2RX7 LILRA2 0.63 1.24e^-31^

P2RX7 FCGR2A 0.78 1.39e^-55^

P2RX7 SIGLEC9 0.82 8.97e^-67^

P2RX7 CD53 0.79 7.15e^-59^

P2RX7 NCKAP1L 0.81 1.43e^-64^

P2RX7 SIGLEC7 0.69 4.77e^-39^

P2RX7 SIGLEC8 0.7 1.19e^-41^

P2RX7 RUNX3 0.65 3.45e^-34^

P2RX7 CD37 0.69 8.33e^-40^

P2RX7 SPI1 0.73 3.59e^-47^

P2RX7 BTN3A1 0.78 1.11e^-57^

P2RX7 IL2 0.42 9.95e^-13^

P2RX7 ISG20 0.53 9.59e^-21^

P2RX7 TNFRSF18 0.36 6.11e^-10^

P2RX7 MMP12 0.19 0.00188

P2RX7 TREM1 0.3 6.3e^-07^

P2RX7 CLEC4E 0.17 0.00412

P2RX7 APOBEC3G 0.31 1.48e^-07^

P2RX7 UBD 0.48 8.78e^-17^

P2RX7 CXCL11 0.55 1.39e^-22^

P2RX7 CXCL9 0.41 3.59e^-12^

P2RX7 CD27 0.37 2.28e^-10^

P2RX7 LY9 0.61 1.62e^-28^

P2RX7 SLAMF7 0.62 1.09e^-29^

P2RX7 NOX4 -0.23 0.00019

P2RX7 SYP 0.19 0.00222

P2RX7 LGALS4 0.17 0.0056

P2RX7 CTSG -0.12 0.04892

P2RX7 BST2 0.4 6.23e^-12^

P2RX7 IRF6 0.19 0.0014

P2RX7 TP73 0.56 4.57e^-24^

P2RX7 FFAR2 0.37 2.25e^-10^

P2RX7 NLRP7 0.23 0.00012

IL6 TRIM60 -0.12 0.04311

IL6 TET2 -0.15 0.01448

IL6 DEFA6 -0.15 0.01128

IL6 PRKCE -0.17 0.00406

IL6 TNFRSF13B -0.2 0.00111

IL6 GLI1 -0.14 0.02263

IL6 TRIM36 -0.17 0.00443

IL6 PPARG -0.18 0.00357

IL6 TNIP3 -0.15 0.0148

IL6 ACAP1 -0.13 0.03521

IL6 LEP -0.2 0.00124

IL6 FLI1 -0.19 0.00196

IL6 LY86 -0.15 0.01321

IL6 ELANE -0.13 0.03493

IL6 PDCD1LG2 -0.12 0.04068

IL6 HIF1AN -0.15 0.01103

IL6 VENTX 0.15 0.01347

IL6 LTB4R 0.24 7.17e^-05^

IL6 LAT 0.14 0.02209

IL6 CTNNAL1 0.19 0.00173

IL6 CD1D 0.17 0.00418

IL6 VEGFA 0.14 0.02007

IL6 MAP3K14 0.15 0.01169

IL6 TRADD 0.28 3.46e^-06^

IL6 OAS1 0.19 0.00149

IL6 TLR3 0.12 0.04

IL6 PELI2 0.14 0.02232

IL6 TRIM9 0.2 0.00121

IL6 SELE 0.21 0.00063

IL6 C7 0.3 6.57e^-07^

IL6 CXCL12 0.21 0.00068

IL6 FCGR1A 0.23 0.00013

IL6 TLR6 0.21 0.00038

IL6 IFNG 0.15 0.0125

IL6 SIGLEC10 0.22 0.00031

IL6 SLAMF8 0.26 1.63e^-05^

IL6 PTPRC 0.19 0.0021

IL6 TRAT1 0.16 0.00787

IL6 WDR62 0.17 0.0043

IL6 CCNA2 0.24 4.57e^-05^

IL6 BIRC5 0.21 0.00052

IL6 PLK1 0.17 0.00485

IL6 C3 0.24 6.28e^-05^

IL6 UCHL1 0.19 0.00194

IL6 IFI6 0.35 2.15e^-09^

IL6 CARD16 0.21 0.00053

IL6 SAMHD1 0.2 0.001

IL6 IFI27 0.17 0.00415

IL6 LILRA2 0.14 0.02353

IL6 FCGR2A 0.29 1.12e^-06^

IL6 SIGLEC9 0.28 2.92e^-06^

IL6 CD53 0.24 5.48e^-05^

IL6 NCKAP1L 0.21 0.00046

IL6 SIGLEC7 0.2 0.00087

IL6 SIGLEC8 0.13 0.03326

IL6 CD37 0.2 0.00091

IL6 SPI1 0.27 4.49e^-06^

IL6 BTN3A1 0.13 0.03219

IL6 IL2 0.26 1.93e^-05^

IL6 ISG20 0.21 0.00062

IL6 TNFRSF18 0.26 1.05e^-05^

IL6 TREM1 0.35 3.12e^-09^

IL6 CLEC4E 0.15 0.01296

IL6 UBD 0.12 0.04809

IL6 LY9 0.21 0.00049

IL6 SLAMF7 0.2 0.00109

IL6 BST2 0.15 0.01091

IL6 TP73 0.12 0.04103

IL6 FFAR2 0.37 3.82e^-10^

PYCARD IL10 -0.12 0.04191

PYCARD EFTUD2 -0.17 0.00534

PYCARD CAMK2A -0.19 0.00197

PYCARD NLRP2 -0.25 3.76e^-05^

PYCARD PROCR -0.29 9.6e^-07^

PYCARD TRIM60 -0.29 7.67e^-07^

PYCARD CD300E -0.2 3.77e^-06^

PYCARD F2RL2 -0.22 0.00022

PYCARD TET2 -0.41 1.43e^-12^

PYCARD DEFA6 -0.37 3.18e^-10^

PYCARD PRKCE -0.42 9.63e^-13^

PYCARD TNFRSF13B -0.47 3.49e^-16^

PYCARD GLI1 -0.33 2.34e^-08^

PYCARD LGALS9 -0.12 0.04776

PYCARD C5AR1 -0.38 1e^-10^

PYCARD TRIM36 -0.41 1.75e^-12^

PYCARD PPARG -0.43 1.66e^-13^

PYCARD TNIP3 -0.36 6.54e^-10^

PYCARD ACAP1 -0.3 3.13e^-07^

PYCARD LEP -0.48 4.65e^-17^

PYCARD FLI1 -0.47 2.27e^-16^

PYCARD LY86 -0.36 9.97e^-10^

PYCARD ELANE -0.31 1.79e^-07^

PYCARD PDCD1LG2 -0.3 3.61e^-07^

PYCARD HIF1AN -0.35 3.31e^-09^

PYCARD PQBP1 -0.25 3.6e^-05^

PYCARD NLRP11 -0.13 0.0313

PYCARD VENTX 0.49 8.78e^-18^

PYCARD LTB4R 0.51 1.72e^-19^

PYCARD YJEFN3 0.39 4.72e^-11^

PYCARD LAT 0.42 3.99e^-13^

PYCARD F11 0.32 6.7e^-08^

PYCARD USP2 0.3 6.1e^-07^

PYCARD F2RL3 0.22 0.0003

PYCARD CTNNAL1 0.34 1.3e^-08^

PYCARD KITLG 0.15 0.01085

PYCARD CD1D 0.47 4.68e^-16^

PYCARD VEGFA 0.24 8.65e^-05^

PYCARD MAP3K14 0.49 7.15e^-18^

PYCARD TRADD 0.64 3.5e^-33^

PYCARD OAS1 0.5 1.86e^-18^

PYCARD ANXA4 0.41 3.58e^-12^

PYCARD TLR3 0.33 4.26e^-08^

PYCARD PELI2 0.29 9.33e^-07^

PYCARD TRIM9 0.31 2.55e^-07^

PYCARD SELE 0.16 0.01047

PYCARD C7 0.4 4.7e^-12^

PYCARD CXCL12 0.36 6.97e^-10^

PYCARD FCGR1A 0.38 8.69e^-11^

PYCARD TLR6 0.41 1.78e^-12^

PYCARD IFNG 0.32 8.49e^-08^

PYCARD SIGLEC10 0.37 3.58e^-10^

PYCARD SLAMF8 0.48 4.56e^-17^

PYCARD PTPRC 0.44 5.07e^-14^

PYCARD TRAT1 0.47 3.94e^-16^

PYCARD WDR62 0.53 5.06e^-21^

PYCARD CCNA2 0.51 1.1e^-19^

PYCARD BIRC5 0.63 1.19e^-31^

PYCARD PLK1 0.52 2.69e^-20^

PYCARD C3 0.41 1.94e^-12^

PYCARD UCHL1 0.37 2.49e^-10^

PYCARD IFI6 0.39 3.85e^-11^

PYCARD CARD16 0.74 5.43e^-49^

PYCARD SAMHD1 0.66 7.66e^-36^

PYCARD IFI27 0.7 5.19e^-42^

PYCARD LILRA2 0.47 5.1e^-16^

PYCARD FCGR2A 0.49 1.37e^-17^

PYCARD SIGLEC9 0.51 1.36e^-19^

PYCARD CD53 0.52 5.54e^-20^

PYCARD NCKAP1L 0.51 2.83e^-19^

PYCARD SIGLEC7 0.39 2.28e^-11^

PYCARD SIGLEC8 0.45 8.27e^-15^

PYCARD RUNX3 0.58 9.45e^-26^

PYCARD CD37 0.71 2.82e^-43^

PYCARD SPI1 0.67 1.4e^-36^

PYCARD BTN3A1 0.43 1.56e^-13^

PYCARD IL2 0.42 3.3e^-13^

PYCARD ISG20 0.62 2.23e^-30^

PYCARD TNFRSF18 0.58 1.53e^-25^

PYCARD MMP12 0.14 0.02101

PYCARD TREM1 0.23 0.00017

PYCARD CLEC4E 0.14 0.02037

PYCARD APOBEC3G 0.28 2.66e^-06^

PYCARD UBD 0.32 9.09e^-08^

PYCARD CXCL11 0.32 8.82e^-08^

PYCARD CXCL9 0.27 5.68e^-06^

PYCARD CD27 0.46 2.07e^-15^

PYCARD LY9 0.55 1.67e^-22^

PYCARD SLAMF7 0.48 3.94e^-17^

PYCARD NOX4 -0.13 0.02744

PYCARD SYP 0.31 1.5e^-07^

PYCARD LGALS4 0.34 6.54e^-09^

PYCARD CTSG 0.24 7.62e^-05^

PYCARD BST2 0.66 1.63e^-35^

PYCARD WNT9B 0.48 4.92e^-17^

PYCARD TP73 0.41 1.74e^-12^

PYCARD FFAR2 0.3 3.6e^-07^

PYCARD NLRP7 0.2 0.00084

CASP1 EFTUD2 -0.26 1.47e^-05^

CASP1 CAMK2A -0.43 1.78e^-13^

CASP1 NLRP2 -0.37 5.43e^-10^

CASP1 PROCR -0.43 8.56e^-14^

CASP1 TRIM60 -0.43 8.07e^-14^

CASP1 CD300E -0.34 1.06e^-08^

CASP1 F2RL2 -0.41 3.45e^-12^

CASP1 TET2 -0.53 2.03e^-21^

CASP1 DEFA6 -0.54 3e^-22^

CASP1 PRKCE -0.61 3.6e^-29^

CASP1 TNFRSF13B -0.69 1.87e^-39^

CASP1 GLI1 -0.5 3.41e^-18^

CASP1 LGALS9 -0.27 4.85e^-06^

CASP1 C5AR1 -0.55 1.66e^-22^

CASP1 TRIM36 -0.61 1.73e^-28^

CASP1 PPARG -0.63 7.76e^-31^

CASP1 TNIP3 -0.54 2.73e^-22^

CASP1 ACAP1 -0.48 8.8e^-17^

CASP1 LEP -0.71 4.92e^-42^

CASP1 FLI1 -0.66 7.23e^-36^

CASP1 LY86 -0.54 3.52e^-22^

CASP1 ELANE -0.46 2.06e^-15^

CASP1 PDCD1LG2 -0.44 2e^-14^

CASP1 HIF1AN -0.51 2.47e^-19^

CASP1 PQBP1 -0.4 6.05e^-12^

CASP1 NLRP11 -0.18 0.00269

CASP1 VENTX 0.59 1.87e^-26^

CASP1 LTB4R 0.61 2.53e^-29^

CASP1 YJEFN3 0.27 4.99e^-06^

CASP1 LAT 0.43 1.94e^-13^

CASP1 F11 0.26 1.19e^-05^

CASP1 USP2 0.19 0.00148

CASP1 F2RL3 0.37 4.87e^-10^

CASP1 CTNNAL1 0.55 1.18e^-22^

CASP1 KITLG 0.42 3.37e^-13^

CASP1 CD1D 0.79 6.77e^-59^

CASP1 VEGFA 0.58 6.84e^-26^

CASP1 MAP3K14 0.71 3.14e^-42^

CASP1 TRADD 0.78 2.67e^-57^

CASP1 OAS1 0.81 1.83e^-65^

CASP1 ANXA4 0.69 7.2e^-40^

CASP1 TLR3 0.77 3.57e^-55^

CASP1 PELI2 0.56 1.69e^-23^

CASP1 TRIM9 0.61 6.92e^-29^

CASP1 SELE 0.36 6.49e^-10^

CASP1 C7 0.34 5.97e^-09^

CASP1 CXCL12 0.57 1.01e^-24^

CASP1 FCGR1A 0.4 1.2e^-11^

CASP1 TLR6 0.76 8.1e^-53^

CASP1 IFNG 0.53 6.79e^-21^

CASP1 SIGLEC10 0.6 4.02e^-28^

CASP1 SLAMF8 0.67 3.63e^-37^

CASP1 PTPRC 0.84 2.7e^-72^

CASP1 TRAT1 0.75 1.26e^-50^

CASP1 WDR62 0.43 1.87e^-13^

CASP1 CCNA2 0.7 6.8e^-41^

CASP1 PLK1 0.3 2.14e^-10^

CASP1 BIRC5 0.48 3.39e^-17^

CASP1 C3 0.62 9.11e^-30^

CASP1 UCHL1 0.32 6.48e^-08^

CASP1 IFI6 0.41 1.39e^-12^

CASP1 CARD16 0.79 4.93e^-60^

CASP1 SAMHD1 0.83 7.57e^-70^

CASP1 IFI27 0.63 5.92e^-32^

CASP1 LILRA2 0.71 1.11e^-43^

CASP1 FCGR2A 0.83 1.58e^-69^

CASP1 SIGLEC9 0.84 1.03e^-73^

CASP1 CD53 0.87 2.36e^-86^

CASP1 NCKAP1L 0.85 1.4e^-78^

CASP1 SIGLEC7 0.72 8.12e^-44^

CASP1 SIGLEC8 0.73 4.98e^-46^

CASP1 RUNX3 0.75 1.19e^-50^

CASP1 CD37 0.82 3.91e^-66^

CASP1 SPI1 0.84 9.61e^-73^

CASP1 BTN3A1 0.86 4.31e^-80^

CASP1 IL2 0.55 8.77e^-23^

CASP1 ISG20 0.65 1.47e^-34^

CASP1 TNFRSF18 0.52 8.84e^-20^

CASP1 TREM1 0.25 3.21e^-05^

CASP1 APOBEC3G 0.36 1.02e^-09^

CASP1 UBD 0.6 4.74e^-28^

CASP1 CXCL11 0.64 2.21e^-32^

CASP1 CXCL9 0.47 2.96e^-16^

CASP1 CD27 0.53 3.86e^-21^

CASP1 LY9 0.7 1.79e^-41^

CASP1 SLAMF7 0.69 2.78e^-39^

CASP1 NOX4 -0.23 0.00015

CASP1 SYP 0.19 0.00178

CASP1 LGALS4 0.17 0.00636

CASP1 BST2 0.56 6.05e^-24^

CASP1 WNT9B 0.14 0.01792

CASP1 IRF6 0.26 1.87e^-05^

CASP1 TP73 0.47 5.41e^-16^

CASP1 FFAR2 0.32 1.11e^-07^

CASP1 NLRP7 0.12 0.04209

P < 0.05 was considered as statistically significant.
