# Supplementary material for: Generation and Analysis of Pyroptosis-Based and Immune-Based Signatures for Kidney Renal Clear Cell Carcinoma Patients, and Cell Experiment
Source: Front Genet. 2022 Feb 24;13:809794. doi: 10.3389/fgene.2022.809794 (PMC8908022; doi:10.3389/fgene.2022.809794)
Supplement: Supplementary file 7 [file DataSheet2.DOCX]

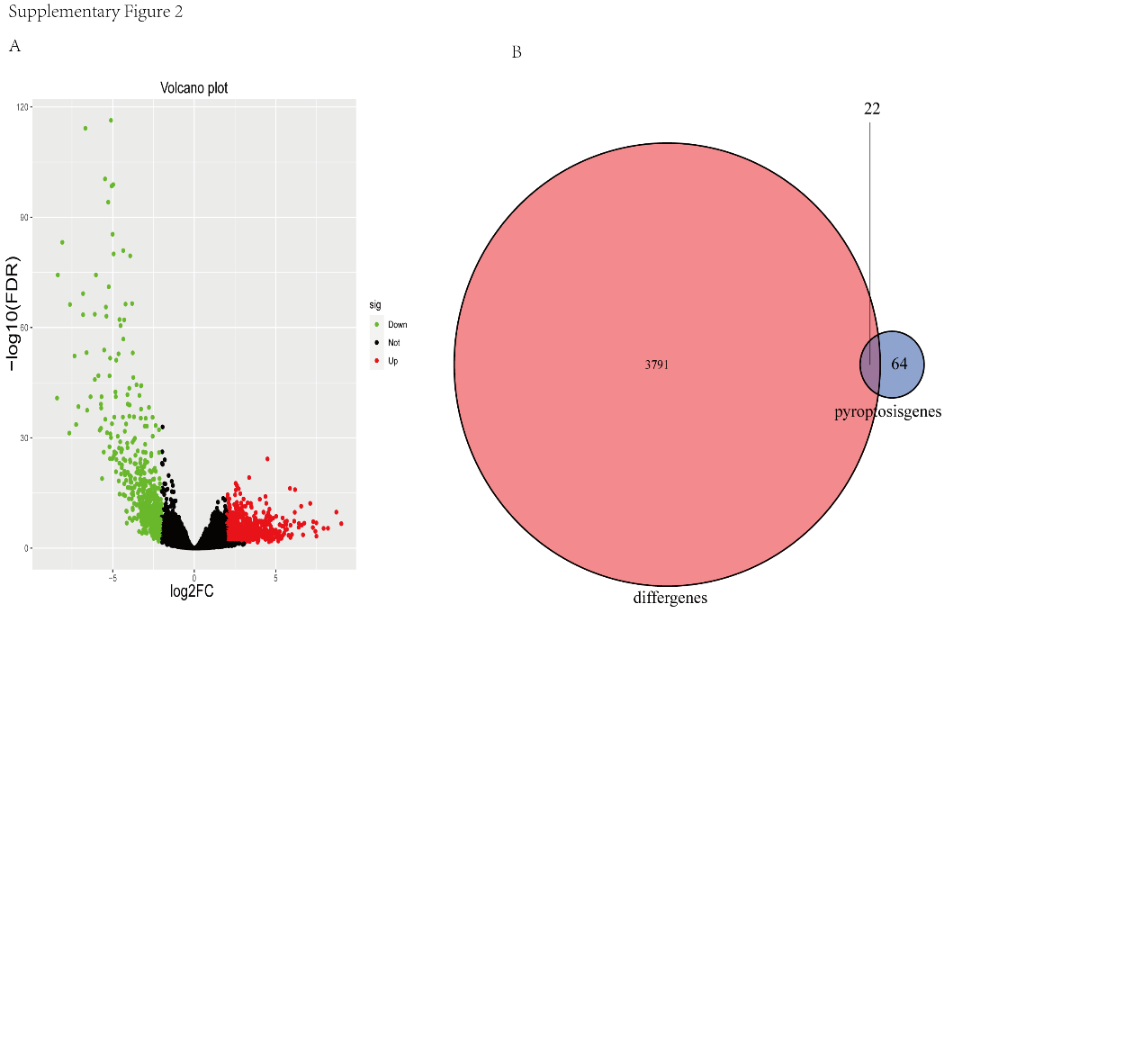


Supplementary Figure 2: Getting DEPAGs. (A) Downregulated, no differentially expressed and upregulated genes of KIRC samples in the training dataset were shown in the volcano plot, which were represented by green, black and red dots, respectively. (B) The Venn diagram shown the intersection of differentially expressed genes with pyroptosis-associated genes.
