# Supplementary material for: Generation and Analysis of Pyroptosis-Based and Immune-Based Signatures for Kidney Renal Clear Cell Carcinoma Patients, and Cell Experiment
Source: Front Genet. 2022 Feb 24;13:809794. doi: 10.3389/fgene.2022.809794 (PMC8908022; doi:10.3389/fgene.2022.809794)
Supplement: Supplementary file 8 [file Table4.DOCX]

Supplementary Table 4: primer sequences

gene primer sequence

GAPDH F: GGAAGGTGAAGGTCGGAGTCA

R: GTCATTGATGGCAACAATATCCACT

AIM2 F: TATTTGGGCATGCTCTCCTGA

R: ACAACTTTGGGATCAGCCTCCT

CASP5 F: TGTTAGCTATGGCTGAAGACAGT

R: TGTCCAGCCACGTTGTTCTT

DFNB59 F: GTTTCACTCTATGGAAGGCGAG

R: GCAATGGAATCTGATCCAGCAA

GSDMC F: AGACAGAGGGGCTCTACAGG

R: CCAGGATGCTCCTTACCAGC

ZBP1 F: AACATGCAGCTACAATTCCAGA

R: AGTCTCGGTTCACATCTTTTGC

CD27 F: AACTGGGCACAGAAAGGAGC

R: TTGCGAACGAGAAGACCAGAG

FCGR1A F: CTGCTCCTTTGGGTTCCAGT

R: TCCTCTTGGAACACGCTGAC

NLRP11 F: AGCAAGATGGCAGAATCGGA

R: CCTCCTGATTGCGGTTTCGT

PRTN3 F: GGCATCTGCTTCGGAGACT

R: CCAGTCCACGTAGAGGGCTA

PSTPIP1 F: TCACTGAGCTCCACTCCTTC

R: TGAAGTCCCTGCACCAAAAGG

TET2 F: CAAGATGGCTGCCCTTTAGGA

R: AATGTTTGCCAGCCTCGTTC

YJEFN3 F: AGCGGCATTTCCTCAGGG

R: ACGGGAACGCCTTGGTCA
