# Supplementary figures and images for: Generation and Analysis of Pyroptosis-Based and Immune-Based Signatures for Kidney Renal Clear Cell Carcinoma Patients, and Cell Experiment
Source: Front Genet. 2022 Feb 24;13:809794. doi: 10.3389/fgene.2022.809794 (PMC8908022; doi:10.3389/fgene.2022.809794)

Supplementary Figure 1


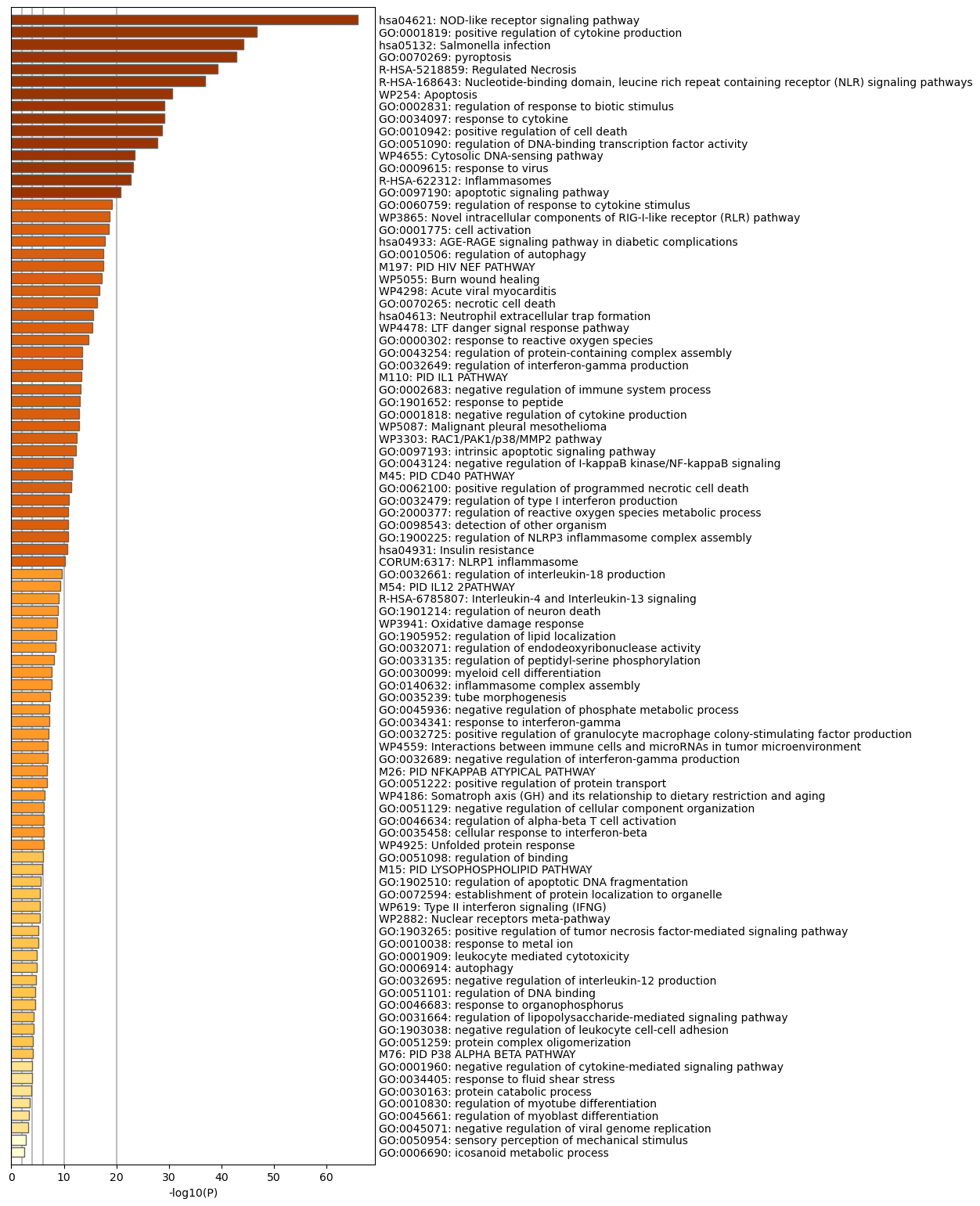


Supplementary Figure 1: GO and KEGG analyses for PAGs.

Supplement: Supplementary file 9 [file DataSheet1.DOCX]
